# Prediction models for mortality in patients with acute on chronic liver failure: systematic review and critical appraisal

**DOI:** 10.3389/fmed.2026.1829188

**Published:** 2026-06-16

**Authors:** Tiantian Song, Jiaqi Zhao, Bei Jiang, Xinyang Wang, Zuokun Li, Qiuyun Pang

**Affiliations:** Department of Intensive Care Unit, The Second Hospital Affiliated Dalian Medical University, Dalian, China

**Keywords:** acute on chronic liver failure, mortality, prediction model, risk score, systematic review

## Abstract

**Background:**

Acute-on-chronic liver failure (ACLF) is a severe syndrome with rapid progression and high short-to-medium-term mortality. Accurate prognostic risk stratification is essential for guiding clinical decisions and optimizing treatment. While numerous prediction models for ACLF have been developed, their performance and clinical applicability remain unclear, warranting a systematic evaluation to guide evidence-based model selection.

**Methods:**

PubMed, Web of Science, Cochrane Library, and Embase were systematically searched from database inception to December 31, 2025. Data extraction and methodological assessment were conducted using the CHARMS. Risk of bias was evaluated using the PROBAST. A meta-analysis of c-statistics was performed using R software (version 4.4.2).

**Results:**

A total of 9,447 studies were identified, with 185 ultimately included, covering 241 external validation cohorts evaluating 75 distinct prognostic models. Approximately 99.51% of analysis units were judged to have a high risk of bias, primarily due to insufficient numbers of outcome events, failure to account for data complexity, and inappropriate assessment of model performance. A total of 24 models met the criteria for meta-analysis at least at one time point, with c-statistics ranging from 0.58 to 0.84. Overall, model discrimination declined with longer prediction horizons, increasing the estimation uncertainty. ACLF-specific models (e.g., CLIF-C ACLF, COSSH ACLF, COSSH ACLF II) showed relatively better discrimination than general models. Among these, the CLIF-C ACLF showed a certain degree of stability across subgroups, though further validation is needed.

**Conclusion:**

The overall risk of bias in the included external validation studies was high, with most lacking calibration reports. Therefore, the current evidence primarily supports relative comparisons of model discrimination, but is insufficient to justify their use as precise probability-based tools for direct clinical decision-making. Most prediction models demonstrated moderate to good discrimination, though their performance declined with longer prediction horizons. COSSH ACLF, COSSH ACLF II, and CLIF-C ACLF showed relatively better discrimination than general models in the available evidence, though this advantage needs further confirmation in higher-quality studies. Future research should focus on well-designed, multicenter validation studies, with systematic evaluation of calibration and long-term predictive performance, to further strengthen the evidence base for ACLF prognostic prediction models.

## Introduction

Acute-on-chronic liver failure (ACLF) is a severe hepatic syndrome distinct from chronic liver failure, characterized by rapid clinical deterioration, frequent development of hepatic and/or extrahepatic multiple organ failure, and exceedingly high mortality (1). The short- to medium-term (1–6 months) mortality of patients with ACLF may reach 50–90% (2). Current management strategies primarily focus on the treatment of precipitating events and complications, organ support for organ failure, and liver transplantation (LT) (3). Increasing evidence suggests that therapeutic decision-making in ACLF should be aligned with individualized prognostic assessment (4). For patients with a relatively favorable prognosis, sustained and aggressive treatment should be provided without hesitation, often serving as a bridge to LT. In contrast, for patients with poor prognostic expectations—particularly in the context of substantial economic burden and limited healthcare resources—palliative care may represent a more appropriate option. The 2023 clinical practice guidelines on ACLF issued by the European Association for the Study of the Liver explicitly recommend systematic prognostic risk assessment to guide and optimize therapeutic strategies for patients with ACLF (5).

To date, multiple prognostic prediction models have been developed to estimate outcomes in patients with ACLF. These include the Model for End-Stage Liver Disease (MELD) (6) and its derivative model (iMELD) (7), originally developed for patients with cirrhosis, as well as the Sequential Organ Failure Assessment (SOFA) score and its derivative models, including CLIF-SOFA (8), CLIF-C OFs, and CLIF-C ACLF (9). These models are constructed based on various combinations of clinical variables, laboratory parameters, and statistical algorithms. Although they have undergone external validation in numerous studies, direct head-to-head comparisons of all models within the same cohort remain scarce. Furthermore, limitations in sample size across individual studies may contribute to inconsistent findings and reduced statistical power. Therefore, integrating evidence from all available external validation studies of the same model is essential to comprehensively evaluate its real-world performance in large populations.

In this study, we aimed to systematically identify all developed and externally validated prognostic prediction models for patients with ACLF and to comprehensively evaluate their performance through meta-analysis. By clarifying the strengths and limitations of different models, we seek to provide more robust and comprehensive evidence to support clinicians in selecting appropriate prognostic prediction tools, ultimately improving the clinical management and outcomes of patients with ACLF.

## Methods

### Study design and reporting framework

This systematic review was designed in accordance with the Checklist for Critical Appraisal and Data Extraction for Systematic Reviews of Prediction Modeling Studies (CHARMS) (10) and the Preferred Reporting Items for Systematic Reviews and Meta-Analyses (PRISMA) (11) statement. The protocol was registered in PROSPERO (registration number: CRD42017069247). However, two modifications were made during the course of the study: first, the literature search end date was extended from July 1, 2024, to December 31, 2025, to ensure the timeliness of the evidence; second, due to limitations in the data reported by the included studies, the subgroup analyses were not fully conducted as originally planned. Ultimately, subgroup analyses were performed based only on cirrhosis status (cirrhotic vs. non-cirrhotic), etiology (chronic hepatitis B vs. others), and definitions (APASL, EASL, and COSSH).

The scope of the review, search strategy, and eligibility criteria were developed according to the PICOTS framework (12, 13). The detailed PICOTS specification was as follows:

(1) Population (P): Patients diagnosed with acute-on-chronic liver failure (ACLF).
(2) Index model (I): Any prognostic prediction model designed to estimate clinical outcomes in patients with ACLF or to identify those at high risk of poor prognosis.
(3) Comparator (C): Not applicable.
(4) Outcome (O): All-cause mortality at any time point during the clinical course of ACLF.
(5) Timing (T): Predictors measured at any stage during the clinical course of ACLF, with outcomes including short-term or long-term mortality; no restriction was imposed on the prediction time horizon.
(6) Setting (S): Hospitalized patients.

### Search strategy

Four electronic databases—PubMed, Web of Science, Cochrane Library, and Embase—were systematically searched from database inception to December 31, 2025. A comprehensive search strategy combining controlled vocabulary terms and free-text keywords was employed to identify studies evaluating prognostic prediction models for ACLF. Search terms included combinations of “acute-on-chronic liver failure,” “mortality,” “prognosis,” “risk assessment,” “risk score,” “prediction model,” and “risk factor.” The detailed search strategy is provided in Supplementary Material 1.

In addition, reference lists of all included studies were manually screened to identify potentially eligible articles not captured in the electronic search.

### Eligibility criteria

All retrieved records were imported into NoteExpress software for deduplication and screening. Two reviewers (Song T, Jiang B) independently screened titles, abstracts, and full texts to determine eligibility. Discrepancies were resolved through discussion or adjudication by the third reviewer (Zhao J).

### Inclusion criteria

(1) External validation studies: Only studies that externally validated existing prognostic prediction models for ACLF using independent datasets were included.
(2) Definition of prediction model: In accordance with the Transparent Reporting of a Multivariable Prediction Model for Individual Prognosis or Diagnosis (TRIPOD) statement (14), eligible models were required to be multivariable models incorporating at least two clinical predictors.
(3) Model type: Both individualized prediction models (IPMs) and risk stratification models (RSMs) were eligible, provided they were capable of predicting mortality risk in patients with ACLF.
(4) Performance reporting: Studies were required to report the c-statistic with corresponding 95% confidence intervals (CIs), or provide sufficient data to calculate the c-statistic and its 95% CI.
(5) Publication type: Only peer-reviewed articles published in English were considered.

### Exclusion criteria

(1) Studies with a sample size of fewer than 100 participants were excluded to enhance precision and reduce the risk of bias.
(2) Non-original research articles, including systematic reviews, meta-analyses, narrative reviews, guidelines, commentaries, letters, conference abstracts, and reports, were excluded.
(3) Animal studies, basic experimental research, and studies involving non-human samples were excluded.
(4) Studies without accessible full texts or with insufficient data were excluded.

### Data extraction

Data extraction was independently performed by two reviewers (Song T, Wang X) using the CHARMS checklist (10). Discrepancies were resolved by consensus or consultation with the third reviewer (Pang Q).

Extracted data included: first author, publication year, study design, study setting, data source, population characteristics (age, sex, etiology, proportion of cirrhosis), predicted outcome, number of events, sample size, model name, modeling method, model presentation format, final predictors included in the model, and performance measures.

When necessary, corresponding authors were contacted to obtain missing data. If multiple models were validated within a single study, data were extracted separately for each model. If a model’s performance was evaluated at multiple time points, data were extracted separately for each prediction horizon. Therefore, in this meta-analysis, we defined a single analysis unit as the validation data of one prediction model in one validation cohort at one time point (i.e., a model-cohort-time point combination). When a model was externally validated across multiple cohorts within a study, only non-overlapping populations were included in the meta-analysis.

### Risk of bias assessment

Risk of bias was independently assessed by two reviewers (Jiang B, Zhao J) using the Prediction Model Risk of Bias Assessment Tool (PROBAST) (13). Disagreements were resolved by the third reviewer (Wang X).

PROBAST evaluates risk of bias in studies that develop, validate, or update diagnostic or prognostic prediction models. It comprises four domains: participants, predictors, outcome, and analysis, and includes 20 signaling questions. Each signaling question is rated as “Yes” or “Probably Yes,” “No” or “Probably No” or “No Information”.

If all signaling questions within a domain were rated as “Yes” or “Probably Yes,” the domain was considered to have low risk of bias. If at least one signaling question was rated as “No” or “Probably No,” the domain was judged to have high risk of bias. If one or more signaling questions were rated as “No Information” without sufficient evidence to assign high risk, the domain was rated as unclear risk.

It is recommended that each prediction model in each study under evaluation, as well as each relevant clinical outcome, be assessed once. Typically, a single article may include one or more study cohorts and perform external validation for one or more prediction models on each relevant outcome separately. Therefore, we treated each independent external validation (i.e., each analytical unit) as a separate unit of evaluation, rather than assessing the risk of bias at the article level.

### Statistical analysis

For each prediction model, a meta-analysis of predictive performance (c-statistic) was conducted if the model had been externally validated in at least three independent datasets.

When the 95% CI or standard error (SE) of the c-statistic was not reported, these values were estimated using established formulas based on the number of events and total sample size (15).

Given the expected heterogeneity across studies in terms of population characteristics, study design, and data sources, a random-effects model was used to pool c-statistics (16). Statistical heterogeneity was assessed using Cochran’s Q test and the *I*^2^ statistic, with *p* < 0.10 for the Q test or *I^2^* > 50% indicating significant heterogeneity (17).

Publication bias was assessed using Egger’s test and funnel plots for prediction models only when at least 10 validation cohorts were included, to avoid reduced statistical power due to small sample size.

To evaluate generalizability and applicability across different clinical contexts, subgroup analyses were conducted according to:

(1) Cirrhosis status (cirrhotic vs. non-cirrhotic)
(2) Etiology (chronic hepatitis B vs. other etiologies)
(3) Definitions (APASL/EASL/COSSH)

For studies with incomplete key data, corresponding authors were contacted twice via email to request additional information.

All statistical analyses were performed using R software (version 4.4.2). Meta-analyses of prognostic prediction models were conducted using the R package “metamisc”.

## Results

### Study characteristics

**Figure 1:**
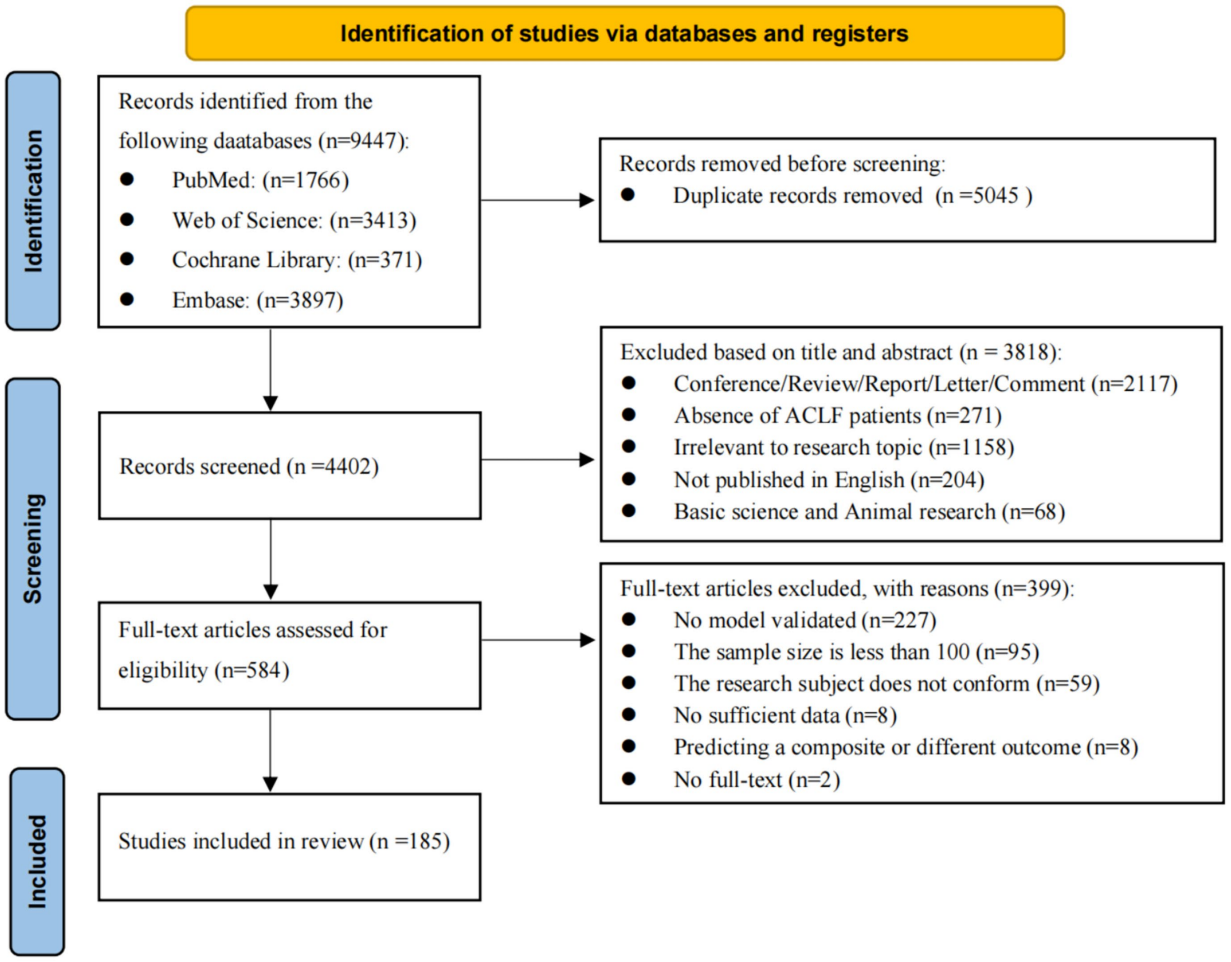
PRISMA flow diagram of study selection process.

As shown in Figure 1, a total of 9,447 records were identified through database searching. After removal of 5,045 duplicate records, 3,818 studies were excluded based on title and abstract screening because they did not meet the inclusion criteria. A total of 584 full-text articles were assessed for eligibility. Ultimately, 185 articles met the predefined eligibility criteria and were included in the systematic review. These studies reported external validation of 75 distinct prognostic prediction models for patients with acute-on-chronic liver failure (ACLF).

This review included 185 studies published from database inception to 2025, encompassing 241 validation cohorts. A total of 75 models were externally validated, and 1,417 analysis units were extracted. The sample size of the validation cohorts ranged from 100 to 4,275 participants. Based on study settings, 75.52% (182/241) of the validation cohorts were single-center cohort. Regarding study design, 26.97% (65/241) of the validation cohorts used a prospective design, 65.14% (157/241) used a retrospective design, and in 7.88% (19/241), the study design could not be determined from the original reports. In terms of geographic distribution, 39.42% (95/241) of the validation cohorts were from China, followed by the United States (4.98%, 12/241) and India (3.32%, 8/241). Chronic hepatitis B was the most common etiology of acute-on-chronic liver failure (ACLF). In more than half of the validation cohorts (160/241), the study population consisted of patients with hepatitis B virus–related ACLF (HBV-ACLF). In 25.73% (62/241) of the validation cohorts, the study population consisted of patients with a history of liver cirrhosis.

Across the outcome time points reported in the included studies, 3-month mortality was the most frequently used time point, with 65 models being validated at this time point, accounting for 86.67% (65/75) of all models. The second most common time point was 1-month mortality, representing 69.33% (52/75). In addition, some studies reported other outcome time points, including 7 days, 2 months, 6 months, 1 year, and 5 years, as well as during hospitalization, during ICU stay, and during follow-up. Detailed characteristics of the included studies are presented in Supplementary Material 2.

### Basic information of the models

Across the 241 external validation cohorts included in this study, a total of 75 distinct prognostic prediction models were evaluated. The earliest applied model was the Child–Turcotte–Pugh (CTP) score, originally proposed in 1973, reflecting the decades-long evolution of prognostic modeling in liver disease.

In recent years, model development has demonstrated a marked upward trend, with 33.33% of the models (25/75) constructed within the past 5 years. According to the country of original development, China contributed the largest number of models, accounting for 52% (39/75), followed by the United States at 17.33% (13/75), while the remaining models were developed across various other countries.

With respect to target populations, 53.33% of the models (40/75) were specifically designed to predict prognosis in patients with acute-on-chronic liver failure (ACLF). The remaining models were not originally developed for ACLF populations; their initial target populations included patients with other liver conditions or broader clinical populations. However, these models were subsequently applied and externally validated in ACLF cohorts, demonstrating potential transportability and clinical extrapolation value in this context.

Regarding model development methods, Cox proportional hazards regression accounted for 42.67% (32/75), while logistic regression accounted for 29.33% (22/75). In addition, several models were developed based on expert consensus or clinical experience rather than traditional statistical modeling. Although not grounded in conventional statistical approaches, these models still retain practical utility in specific clinical scenarios.

The presentation formats of the models were heterogeneous, including regression-based equations, point-based scoring systems, and nomograms. These formats differ in terms of information visualization and clinical operability, potentially influencing their usability in routine practice.

Detailed characteristics of all included models are provided in Supplementary Material 3.

### Risk of bias assessment

A total of 1,417 risk-of-bias assessments were performed using the PROBAST tool in this study. Among these, only 7 (0.49%) were rated as low risk of bias, while the remaining 1,410 (99.51%) were classified as high risk of bias. These findings suggest that the vast majority of external validation studies have substantial methodological limitations, with a generally high risk of bias.

**Figure 2:**
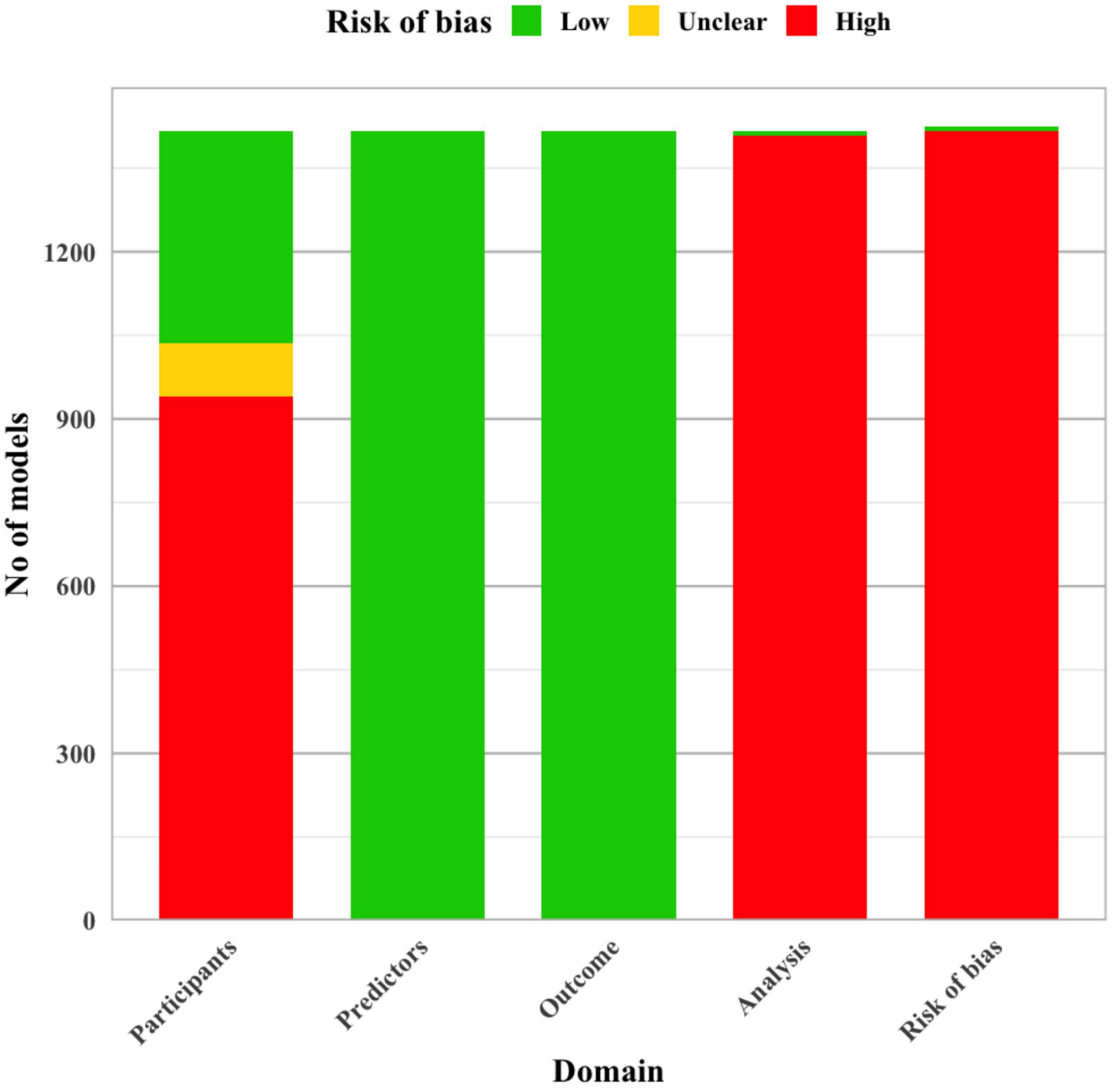
Distribution of risk of bias.

Figure 2 presents the distribution of risk of bias across the four PROBAST domains. Owing to the retrospective cohort design, the participant’s domain was rated as high risk in 940 assessments (66.34%). The predictors and outcomes domains demonstrated relatively lower risk of bias and were generally methodologically robust. However, the analysis domain showed an exceptionally high risk of bias, with 1,409 analysis units (99.44%) rated as high risk. This was primarily attributable to insufficient numbers of outcome events (n < 100), failure to account for data complexity (e.g., censoring and competing risks), and inappropriate evaluation of model performance. In particular, the lack of calibration assessment was a major contributor to high risk in the analysis domain, with 1,273 external validation analyses (89.84%) not reporting any evaluation of model calibration performance.

### Data synthesis

In this systematic review, 51 models (approximately 68%, 51/75) had fewer than three external validation cohorts at each outcome time point. To ensure the robustness and reliability of pooled performance estimates, these models were not included in the quantitative meta-analysis and were instead summarized using a qualitative description.

Across the external validation of these models, a total of 50 independent validation cohorts were involved. All validation cohorts reported population characteristics, sample size, number of events, the c-statistic with its 95% confidence interval, as well as the predicted outcome measures. The evaluated outcomes spanned multiple clinically relevant time points, including 7-day mortality, 1-month mortality, 2-month mortality, 3-month mortality, 6-month mortality, 1-year mortality, 5-year mortality, in-hospital mortality, intensive care unit (ICU) mortality, and mortality during follow-up.

Across these 50 validation cohorts, the sample size ranged from 100 to 980 patients, and all cohorts reported c-statistics of the models with corresponding 95% confidence intervals, with c-statistic values ranging from 0.157 to 0.980.

Overall, these validation results showed substantial variability in discrimination. While some models demonstrated good discrimination, others exhibited limited predictive ability. Future studies should accumulate more independent external validation data to further clarify the generalizability and clinical applicability of these models.

## Results of meta-analysis of model validation cohorts

### Results stratified by model

**Figure 3:**
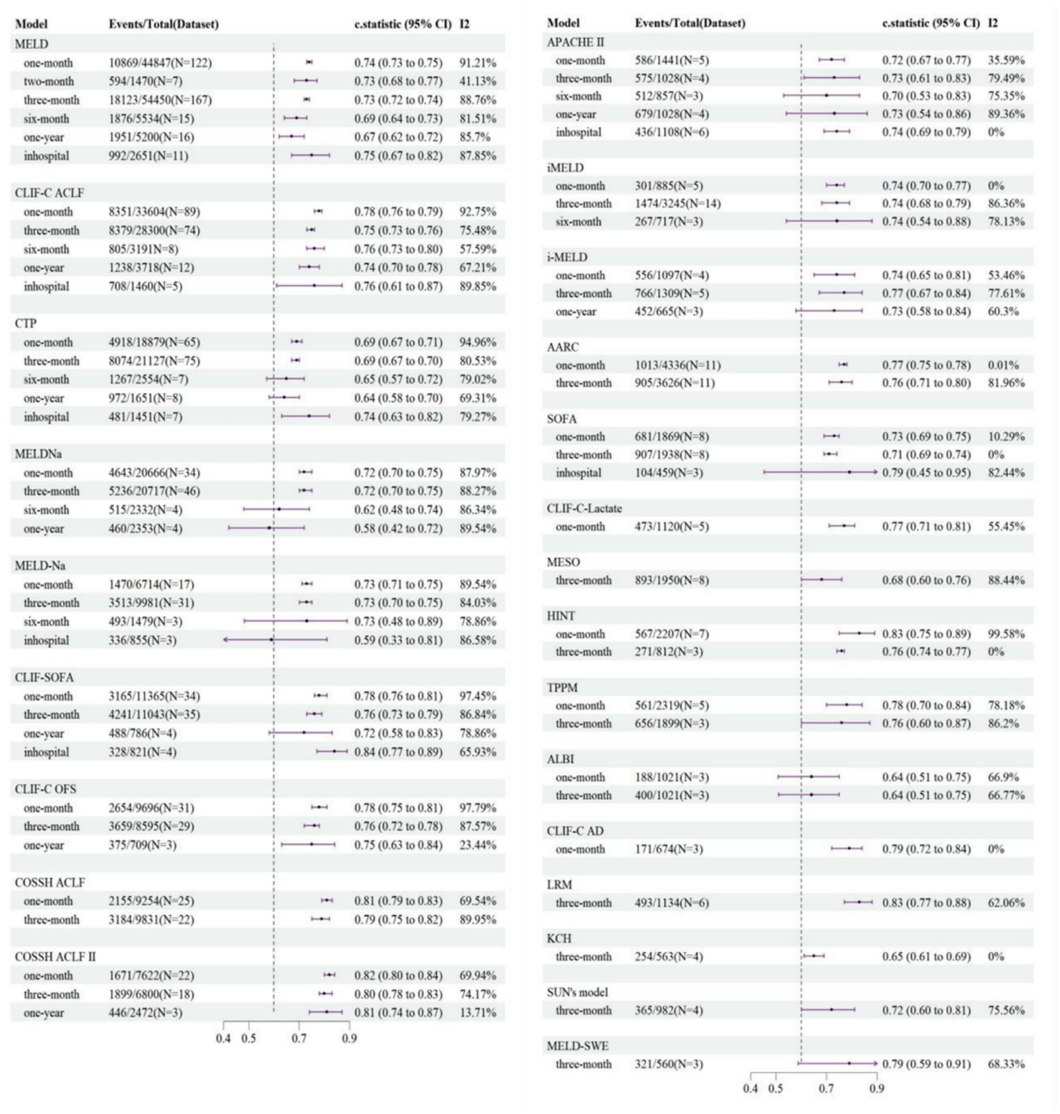
Meta-analysis of the prediction model (stratified by model).

As shown in Figure 3, the discriminative performance varied across the 24 prediction models. The pooled c-statistics ranged from 0.58 (for MELD-Na: 95% CI, 0.42–0.72) to 0.84 (for CLIF-SOFA: 95% CI, 0.77–0.89). Forest plots of pooled c-statistics for the 24 models at different time points are shown in Supplementary Material 6.

From a temporal perspective, model discrimination generally declined with increasing prediction horizons. Moreover, for intermediate- and long-term outcomes (6-month and 1-year mortality), the pooled estimates were characterized by wider confidence intervals, indicating greater statistical uncertainty and reduced precision.

In this study, the models were further stratified according to their development population into those specifically developed for ACLF patients and general models derived from non-ACLF populations. Among the general models, the most frequently externally validated models included CTP, MELD, CLIF-SOFA, and CLIF-C OFS, with an overall c-statistic ranging from 0.64 to 0.79.

Notably, the CTP score demonstrated relatively poor discriminative performance (pooled c-statistic: 0.64–0.69), with a more pronounced decline in long-term prediction and limited estimation precision.

In comparison, MELD, CLIF-SOFA, and CLIF-C OFS showed moderate discriminative performance in short-term prediction. (pooled c-statistics: 0.73–0.78). However, their performance decreased at intermediate- and long-term ontcomes (pooled c-statistics: 0.67–0.75). Notably, the 95% confidence intervals for CLIF-SOFA and CLIF-C OFS widened substantially at longer follow-up periods, suggesting reduced precision in long-term prediction.

In contrast, models specifically developed for patients with ACLF (ACLF-specific models) demonstrated overall superior discriminative performance (pooled c-statistic: 0.72–0.82). Among these, CLIF-C ACLF, COSSH ACLF, and COSSH ACLF II were supported by a larger number of external validation cohorts and exhibited relatively smaller declines in discriminative ability over time (pooled c-statistic: 0.74–0.82), indicating greater temporal stability. COSSH ACLF II and COSSH ACLF achieved the highest discrimination at 1 month and 3 months (pooled c-statistic > 0.79). However, due to limited long-term outcomes, their performance for extended follow-up periods requires further investigation.

### Results stratified by time horizon

When comparing models at the same outcome time point, substantial heterogeneity in discriminative performance was also observed across different prediction models. The HINT model demonstrated high discriminative ability for 1-month mortality (pooled c-statistic: 0.83, 95% CI: 0.75–0.89), and the LRM model showed similarly strong performance for 3-month mortality prediction (pooled c-statistic: 0.83, 95% CI: 0.77–0.88); however, both exhibited relatively wide confidence intervals, indicating limited precision of estimation.

**Figure 4:**
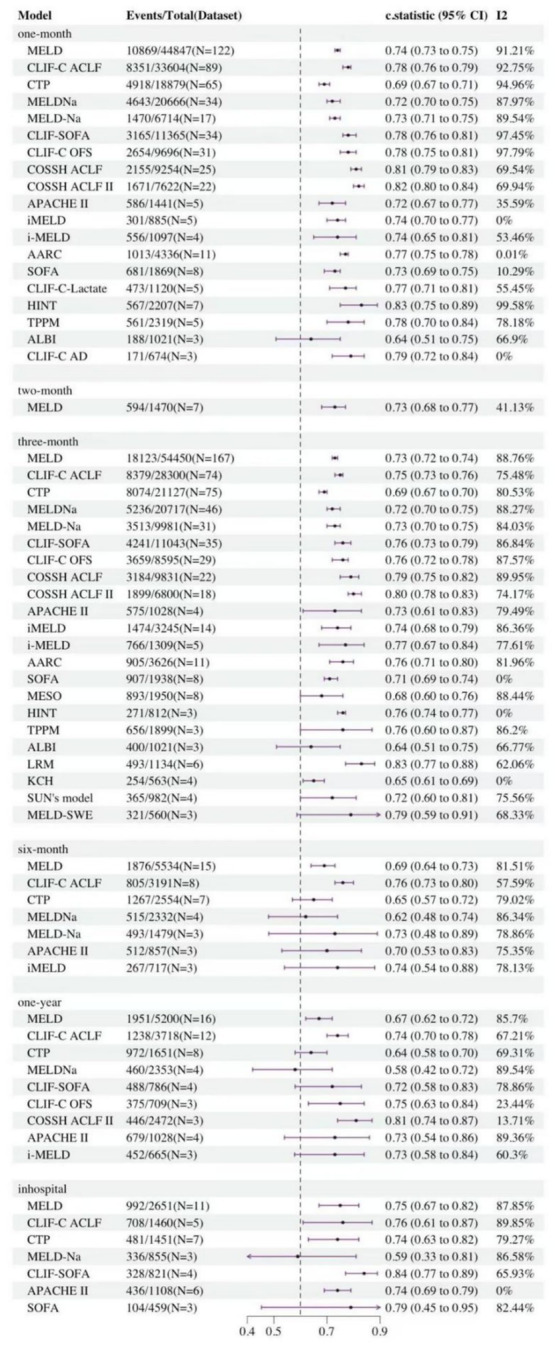
Meta-analysis of the prediction model (stratified by time horizon).

In contrast, the COSSH ACLF II, COSSH ACLF, CLIF-C OFS, CLIF-SOFA, CLIF-C ACLF, and AARC models showed slightly lower discriminative ability in short-term prediction (pooled c-statistic: 0.75–0.82), but with narrower confidence intervals, suggesting relatively greater precision and consistency of estimation. For medium- to long-term follow-up (6-month and 1-year outcomes), the CLIF-C ACLF and CLIF-C OFS models demonstrated relatively better discriminative performance (pooled c-statistic: 0.74–0.76), with CLIF-C ACLF model showing relatively higher precision and predictive reliability (Figure 4).

### Subgroup analysis

Across subgroup populations, the results of model performance were generally consistent with the main analysis, showing a decline in discriminative ability with increasing time horizon and suggesting potential limitations in the robustness of long-term predictions. Notably, the CLIF-C ACLF model demonstrated relatively stable discriminative performance across all subgroups, with minimal fluctuations, indicating comparatively good generalizability.

In the HBV-related population, the HINT and LRM models achieved the best performance at 1-month and 3-month mortality, respectively (pooled c-statistic > 0.83), although both showed limited precision in estimation. In contrast, the COSSH ACLF II, COSSH ACLF, CLIF-C ACLF, CLIF-SOFA, and CLIF-C OFS models demonstrated relatively high discriminative ability (pooled c-statistic > 0.76) and estimation precision in short-term prediction, with COSSH ACLF II and COSSH ACLF achieving (pooled c-statistics exceeding 0.80; Supplementary Figure 1). However, in non-HBV etiological subgroups, the predictive performance of these models for short-term mortality generally declined slightly, accompanied by reduced estimation precision (Supplementary Figure 2).

In the cirrhosis subgroup, the overall predictive patterns of the MELD and CLIF-C ACLF models were broadly consistent with those observed in the main analysis. Both models demonstrated moderate discriminative ability for short-term mortality prediction (pooled c-statistic: 0.70–0.75), while exhibiting relatively high estimation precision and good stability. In contrast, the CLIF-SOFA, CLIF-C OFS, iMELD, and CLIF-C-Lactate models showed pooled c-statistics ranging from 0.74 to 0.78 for 1-month and 3-month outcomes, indicating higher discriminative performance; however, their wider confidence intervals suggested greater uncertainty and relatively lower stability (Supplementary Figure 3).

In the non-cirrhosis subgroup, the COSSH ACLF II, COSSH ACLF, CLIF-C ACLF, CLIF-SOFA, CLIF-C OFS, and AARC models all demonstrated relatively high discriminative ability for short-term mortality prediction (pooled c-statistic > 0.76), with comparatively good estimation precision (Supplementary Figure 4).

In the APASL-defined subgroup, the HINT and LRM models demonstrated the best performance at 1-month and 3-month mortality, respectively (pooled c-statistic > 0.83), although both showed limited precision. In contrast, the COSSH ACLF II, COSSH ACLF, CLIF-C ACLF, CLIF-SOFA, CLIF-C OFS, and AARC models exhibited relatively high discriminative ability for short-term mortality prediction (pooled c-statistic > 0.74) and generally robust estimates, with COSSH-ACLF II and COSSH-ACLF achieving c-statistics exceeding 0.80 (Supplementary Figure 5).

In the EASL-defined subgroup, the CLIF-C-Lactate and CLIF-SOFA models showed the best performance at 1-month and 3-month outcomes, respectively (pooled c-statistic > 0.77), although with limited precision. The CLIF-C ACLF model demonstrated relatively high discriminative ability (pooled c-statistic > 0.73) for short-term mortality prediction, together with stable and robust estimates (Supplementary Figure 6).

In the COSSH-defined subgroup, the COSSH ACLF II, COSSH ACLF, and CLIF-C ACLF models consistently showed relatively high discriminative performance for short-term prediction (pooled c-statistic > 0.77), accompanied by good estimation precision (Supplementary Figure 7).

To further systematically summarize the characteristics of all prediction models and their clinical applicability across different populations, this study comprehensively described the number of external validation studies, prediction time horizons, pooled c-statistics, and suggested clinical application scenarios (Supplementary Material 5).

### Publication bias

In the assessment of publication bias, funnel plots and Egger’s tests were performed only for prediction models with at least 10 validation cohorts. The results showed that the effect sizes of the included studies were approximately symmetrically distributed in the funnel plots. Egger’s test also revealed no significant publication bias (*p* > 0.05).

The funnel plots for the CLIF-C ACLF model at each time point appeared relatively symmetrically distributed, and Egger’s test revealed no significant publication bias (*p* > 0.05; Figure 5). Results for the remaining models are presented in the Supplementary Figures 8–28.

**Figure 5:**
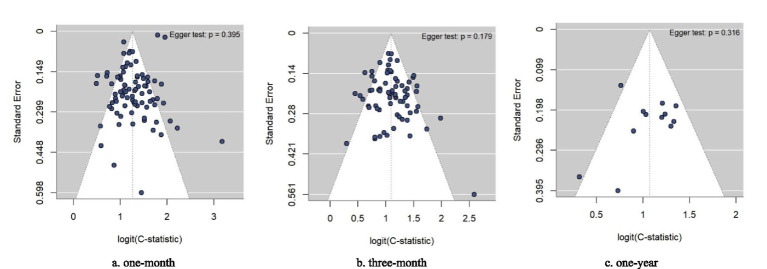
Funnel plot of the CLIF-C ACLF model.

## Discussion

In this systematic review and meta-analysis, we integrated 185 studies encompassing 241 external validation cohorts and 1,417 independent external validation for 75 prediction models of ACLF. To our knowledge, this represents the first systematic synthesis of external validation evidence for ACLF prognostic models. Our findings indicate that, despite the relatively large number of developed models, the research landscape remains markedly unbalanced. The majority of studies have focused on model development, while evidence from external validation remains limited. Only 24 models had three or more independent external validations, sufficient to support quantitative meta-analysis. This “development-heavy, validation-light” pattern is a common issue in prediction model research (18). The rapid increase in model numbers has not been matched by accumulation of high-quality, reproducible external validation evidence, thereby limiting reliable assessment of true performance and generalizability, and leaving clinical applicability uncertain (19). Accordingly, we emphasize the need for future studies to prioritize rigorous and extensive external validation.

Through systematic integration of external validation data for 24 prognostic prediction models, we comprehensively compared their discriminative performance. Pooled c-statistics ranged from 0.58 to 0.84, confirming notable performance differences among models. General models such as MELD, CLIF-SOFA, and CLIF-C OFs demonstrated moderate discriminative ability (pooled c-statistic: 0.73–0.78), suggesting continued utility for risk stratification in ACLF patients. In contrast, models specifically developed for ACLF populations—CLIF-C ACLF, COSSH ACLF, and COSSH ACLF II—showed relatively higher discrimination. The series of COSSH ACLF model achieved c-statistics exceeding 0.79 for short-term outcomes, whereas CLIF-C ACLF model maintained relatively stable moderate-to-high discriminative ability across multiple follow-up time points. These differences likely reflect both the alignment of variable selection with the multi-organ failure and systemic inflammatory characteristics of ACLF, and the methodological rigor employed in model construction. Beyond model-related factors, inconsistency in ACLF diagnostic definitions may also be an important source of heterogeneity in the overall findings. Patients identified by different definitions may differ in disease stage, acute precipitating events, cirrhosis status, and severity of organ failure (20). Therefore, when interpreting the overall pooled performance of different prediction models and the differences between models, the potential impact of heterogeneity in ACLF definitions on the stability, comparability, and generalizability of the results should be fully considered.

However, although differences in discrimination were observed, the magnitude of variation in c-statistics among models was limited, and most remained within the moderate discrimination range. Therefore, conclusions regarding overall predictive performance should be interpreted cautiously. We also observed that discriminative ability does not always align with estimation precision. Some models demonstrated high c-statistics at specific time points but wide 95% confidence intervals, indicating limited precision of the estimates. Conversely, models with slightly lower c-statistics often had narrower confidence intervals and more consistent estimates. Similar findings have been reported in meta-analyses of prediction models for other diseases (21). Thus, model evaluation and clinical selection should not rely solely on point estimates but should consider precision and consistency (22).

Beyond discrimination, calibration—reflecting agreement between predicted and observed risk—is another critical metric for assessing prediction models. Almost all external validation studies overlooked this key aspect (14). Without reliable calibration data, clinicians may remain unaware if a model systematically overestimates or underestimates actual mortality risk, potentially leading to misclassification, overtreatment, or undertreatment.

Further analyses indicated that most models achieved moderate or better discrimination for short-term outcomes, but predictive performance generally declined for long-term endpoints, accompanied by increased estimation uncertainty. This trend aligns with observations from prior literature (23). The decline in performance is unlikely to be solely attributable to sample differences, but rather reflects inherent limitations in the construction strategies of most current prediction models. Most included models were built using baseline or single-time-point clinical variables, essentially representing “static prediction.” However, ACLF is a highly dynamic clinical syndrome, with organ function, inflammatory burden, and treatment responses capable of significant fluctuations in early stages. Over time, baseline information loses explanatory power for long-term outcomes (24–26). Therefore, the observed decline in discriminative ability with extended prediction horizons is theoretically interpretable.

Notably, recent studies have begun exploring prediction strategies based on time-dependent variables or longitudinal data updating to enable dynamic risk assessment. By integrating repeatedly measured clinical indicators during follow-up, these approaches hold promise for improving stability in intermediate- and long-term prediction. Future research should emphasize capturing dynamic changes in disease trajectory during model development to enhance the predictive performance and clinical utility of ACLF prognostic models.

Subgroup analyses generally mirrored the trends observed in the primary analysis, although model performance varied across different patient subgroups. In the HBV-ACLF population, the series of COSSH ACLF models (COSSH ACLF II and COSSH ACLF) demonstrated relatively good (pooled c-statistic > 0.80). However, when applied to non-HBV populations, the performance of these models declined. This performance reduction is primarily attributable to the fact that the COSSH ACLF models were specifically developed in HBV-ACLF cohorts, with variable selection and weighting optimized for clinical features associated with HBV-related acute exacerbations. They thus represent a highly specialized tool targeting a specific pathophysiological mechanism. In contrast, the CLIF-C ACLF model exhibited relatively good robustness across both HBV and non-HBV populations, reflected by narrower 95% confidence intervals and smaller fluctuations in performance. This likely stems from its development using the multicenter, multi-etiology CANONIC cohort, making it more generalizable to ACLF patients with diverse etiologies.

In the cirrhosis subgroup, the MELD and CLIF-C ACLF models demonstrated relatively moderate but stable discriminative performance for short-term mortality prediction. In contrast, several models (including CLIF-SOFA, CLIF-C OFS, iMELD, and CLIF-C-Lactate) showed slightly higher discriminative ability but relatively lower stability. These findings suggest that their application in clinical practice should be interpreted cautiously and considered from multiple perspectives.

In the non-cirrhosis subgroup, most models (including COSSH ACLF II, COSSH ACLF, CLIF-C ACLF, CLIF-SOFA, CLIF-C OFS, and AARC) demonstrated relatively high discriminative ability for short-term mortality prediction (pooled c-statistic > 0.76). These models may have potential clinical utility in practice. However, some models still require further validation for their performance in long-term prognostic prediction.

Subgroup analyses based on different ACLF diagnostic criteria revealed marked heterogeneity in model performance across subgroups and follow-up time points, suggesting that predictive accuracy is both definition-dependent and time-dependent.

In the APASL-defined subgroup, the HINT and LRM models demonstrated the highest discriminative ability for 1-month and 3-month short-term mortality prediction (pooled c-statistic > 0.83), indicating strong sensitivity for early prognostic stratification in this population. However, both models showed limitations in overall predictive precision, which may be attributable to relatively simplified variable structures or insufficient integration of multi-organ failure information (27). In contrast, the COSSH ACLF II, COSSH ACLF, CLIF-C ACLF, CLIF-SOFA, CLIF-C OFS, and AARC models exhibited relatively stable discriminative performance for short-term mortality prediction (pooled c-statistic > 0.74). Among these, COSSH-ACLF II and COSSH-ACLF performed best (pooled c-statistic > 0.80), suggesting advantages in integrating hepatic function, systemic inflammation, and organ failure, and indicating their potential suitability for risk stratification under the APASL framework.

In the EASL-defined subgroup, the CLIF-C-Lactate and CLIF-SOFA models demonstrated the best performance for 1-month and 3-month mortality prediction, respectively (pooled c-statistic > 0.77), although overall precision remained limited, suggesting insufficient predictive stability. In contrast, the CLIF-C ACLF model exhibited relatively more consistent discriminative ability and greater stability in short-term mortality prediction (pooled c-statistic > 0.73), indicating that its organ failure–based integrated scoring structure may be relatively better adapted to the EASL-defined population (28).

In the COSSH-defined subgroup, the COSSH ACLF II, COSSH ACLF, and CLIF-C ACLF models all showed relatively high and stable predictive performance (pooled c-statistic > 0.77), suggesting that COSSH models derived from Chinese population characteristics may have relatively better applicability within this definitional framework.

Meanwhile, the CLIF-C ACLF model maintained relatively good external performance across definitions, indicating that it may have favorable generalizability across different ACLF classification systems.

### Limitations

The included studies were generally subject to a high risk of bias, mainly attributable to the characteristics of time-to-event data and limitations in sample size, as well as the lack of corresponding calibration metrics. Nearly 90% of the studies did not report calibration metrics, suggesting that current research places greater emphasis on risk stratification ability while overlooking the accuracy of predicted probabilities. In the absence of sufficient calibration information, ranking models solely based on the c-statistic may not fully reflect their clinical utility. Overall, this study systematically synthesized existing evidence and compared the discriminative ability of multiple ACLF prognostic prediction models across different subgroups; however, due to limitations in study design, sample size, and population-related bias, the pooled results should be interpreted with caution.

In addition, most of the included studies involved high mortality rates and exhibited clear time-to-event characteristics. If appropriate survival analysis methods were not applied or if censoring and competing risks were not adequately addressed, model performance may have been overestimated.

Furthermore, the majority of included studies originated from Asian cohorts, with HBV-related ACLF as the predominant etiology. This introduces potential demographic bias, as differences in etiology spectrum, healthcare resources, and treatment strategies may exist compared with European or North American populations. Therefore, the external generalizability of these findings across different geographic regions and etiological backgrounds requires further validation in large, multicenter, and cross-regional cohorts.

Finally, this study only searched English-language databases and did not include Chinese databases. Although English is the standard language for biomedical publications and internationally indexed studies are generally of higher methodological rigor, the exclusion of Chinese-language literature may introduce language bias and potentially overestimate the discriminative performance of existing models in Chinese HBV-ACLF populations, given that most validation cohorts in this review originated from China and were predominantly HBV-related.

## Conclusion

The overall risk of bias among the included external validation studies was high, and calibration was not reported in the vast majority of studies. Therefore, the current evidence primarily supports relative comparisons of model discrimination for ACLF prognostic prediction models, but is insufficient to justify their use as precise probability-based tools for direct clinical decision-making. Within this context, 75 prognostic models were identified for risk stratification in patients with ACLF. Most models demonstrated moderate to good discrimination in short-term prediction; however, their performance tended to decline with longer prediction horizons. Compared with general models developed in non-ACLF populations, COSSH-ACLF, COSSH-ACLF II, and CLIF-C ACLF showed relatively better discrimination in the available evidence, although this apparent advantage requires further validation in higher-quality studies. Future research should focus on well-designed, multicenter external validation studies, with systematic evaluation of calibration and long-term predictive performance, to further strengthen the evidence base for ACLF prognostic prediction models.

## Data Availability

The original contributions presented in the study are included in the article/Supplementary material, further inquiries can be directed to the corresponding author/s.
