# Supplementary material for: Prediction models for mortality in patients with acute on chronic liver failure: systematic review and critical appraisal
Source: Front Med (Lausanne). 2026 Jun 16;13:1829188. doi: 10.3389/fmed.2026.1829188 (PMC13314772; doi:10.3389/fmed.2026.1829188)
Supplement: Supplementary file 3 [file Table_3.DOCX]

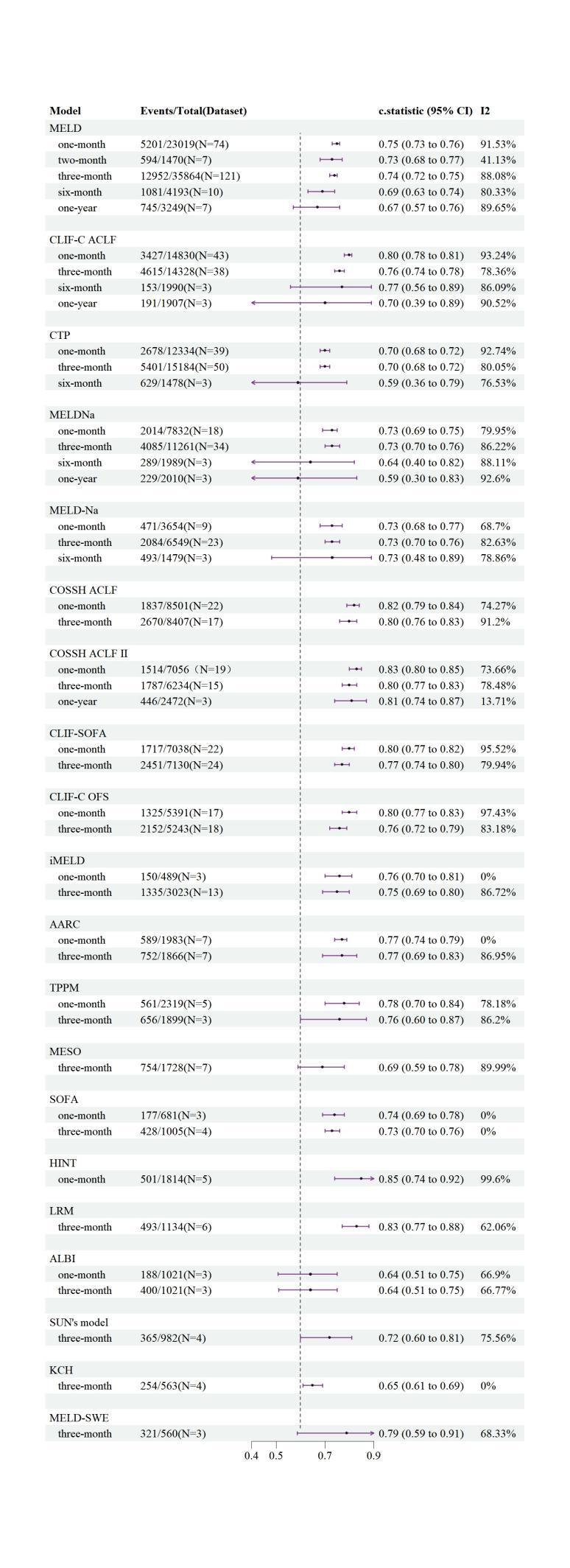


Figure 1. Forest plot of meta-analysis for prognostic prediction models in hepatitis B virus-related acute-on-chronic liver failure (HBV-ACLF) patients.


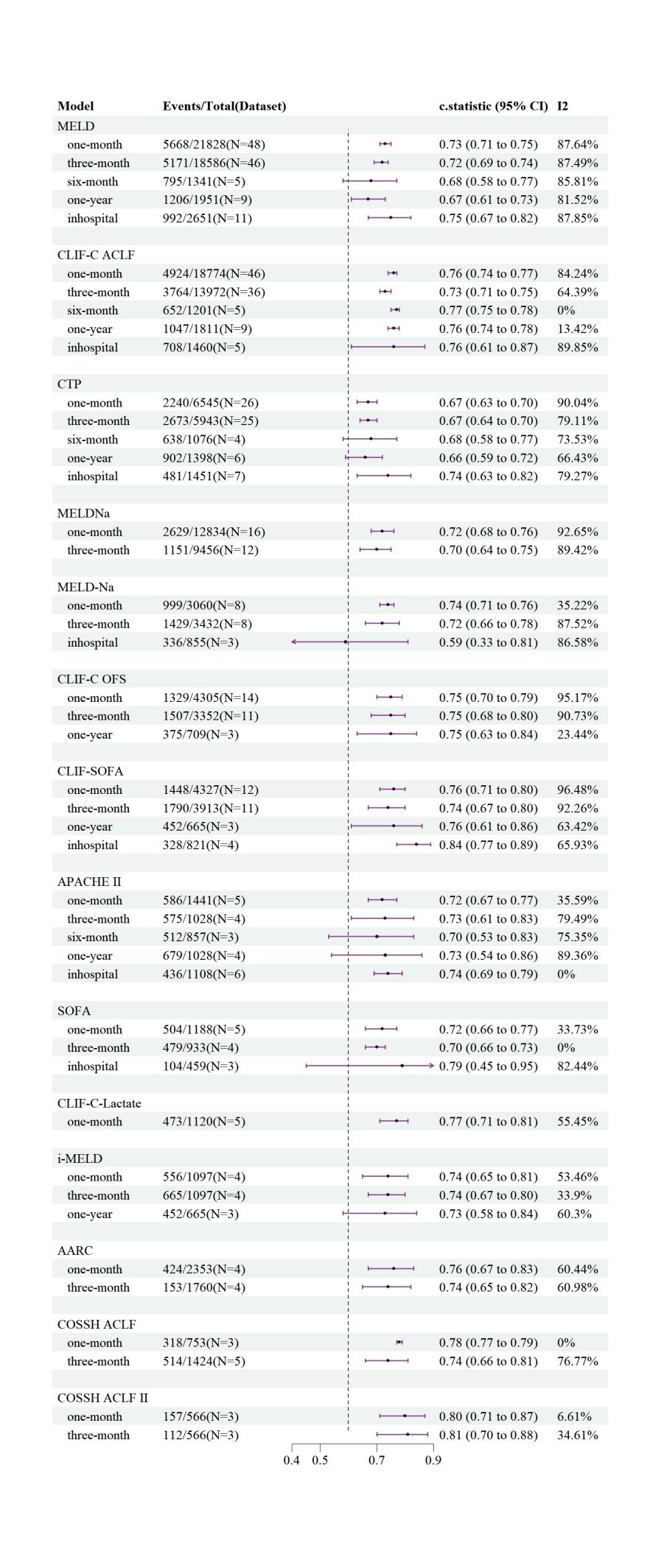


Figure 2. Forest plot of meta-analysis for prognostic prediction models in acute-on-chronic liver failure patients with non-hepatitis B virus etiologies (non-HBV ACLF).


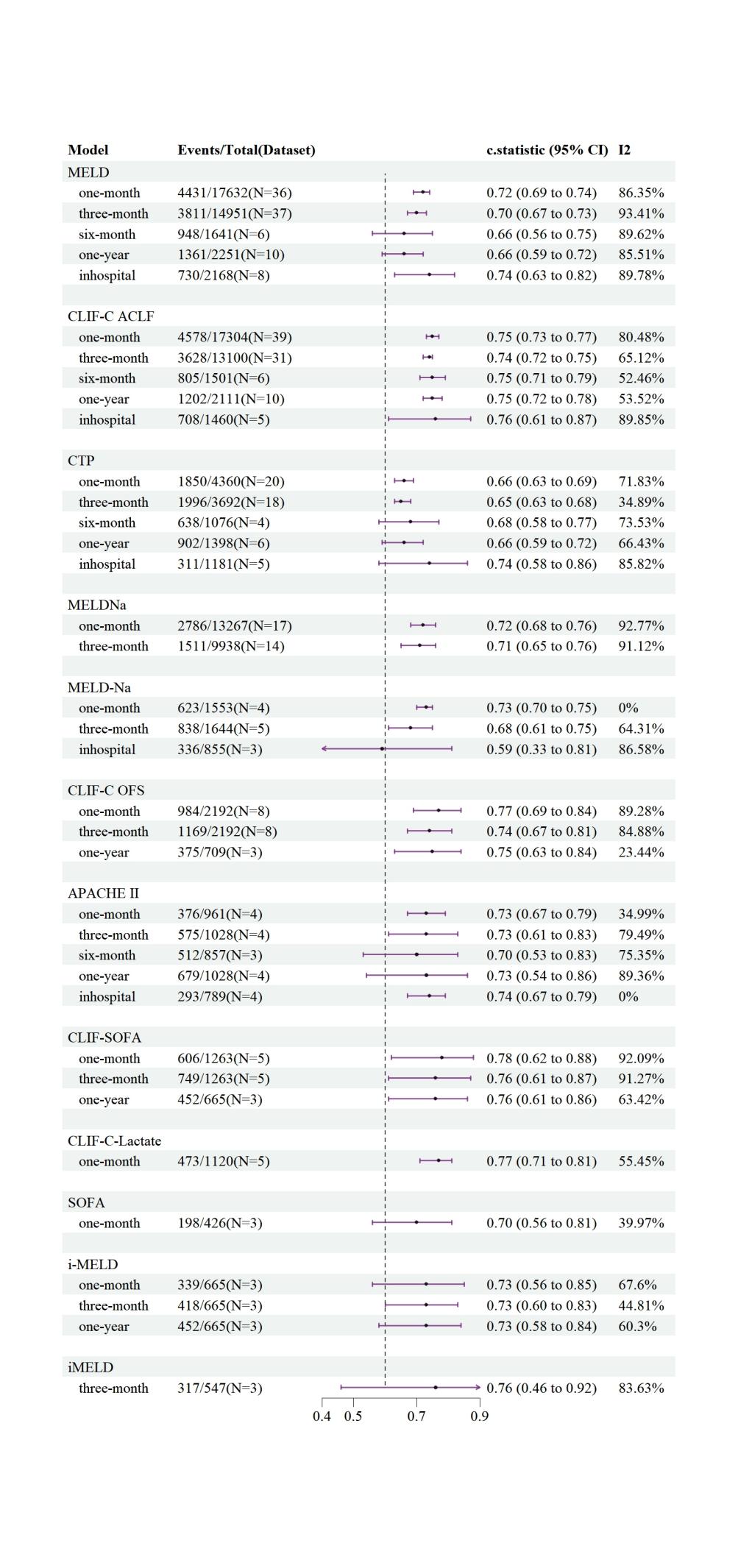


Figure 3. Forest plot of meta-analysis for prognostic prediction models in acute-on-chronic liver failure patients with underlying cirrhosis.


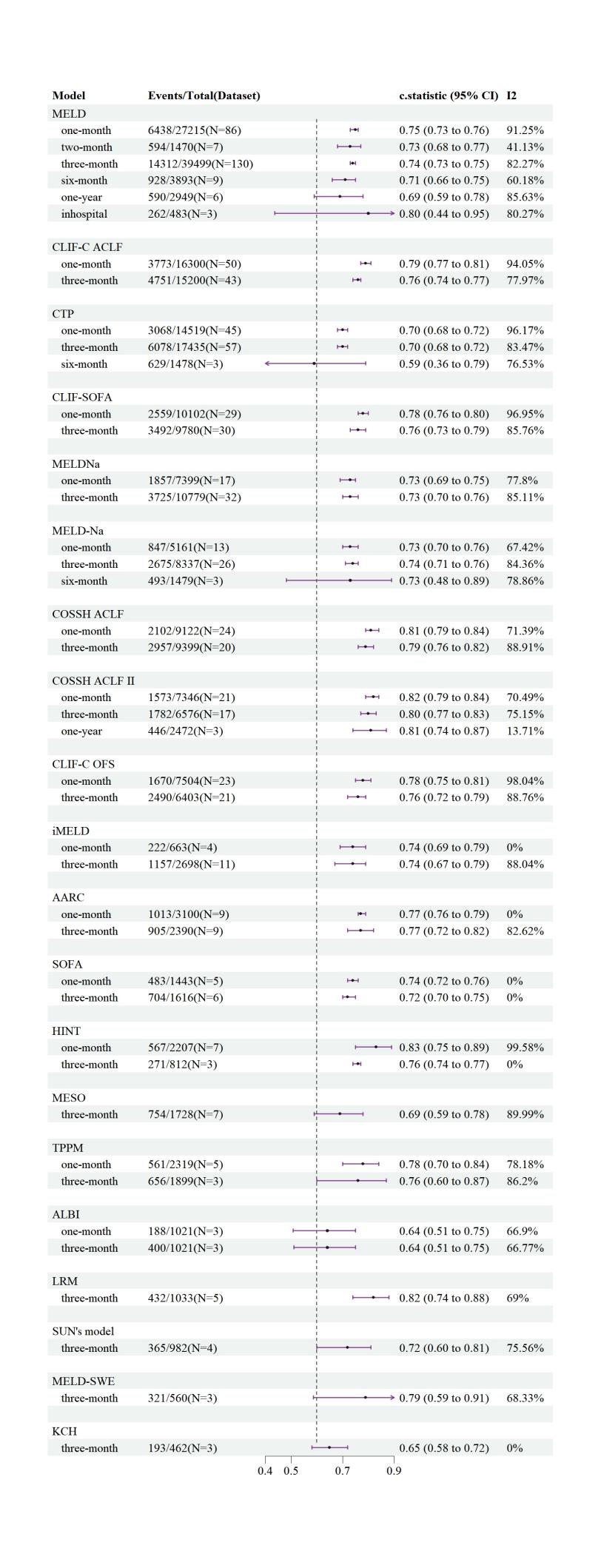


Figure 4. Forest plot of meta-analysis for prognostic prediction models in acute-on-chronic liver failure patients without underlying cirrhosis.


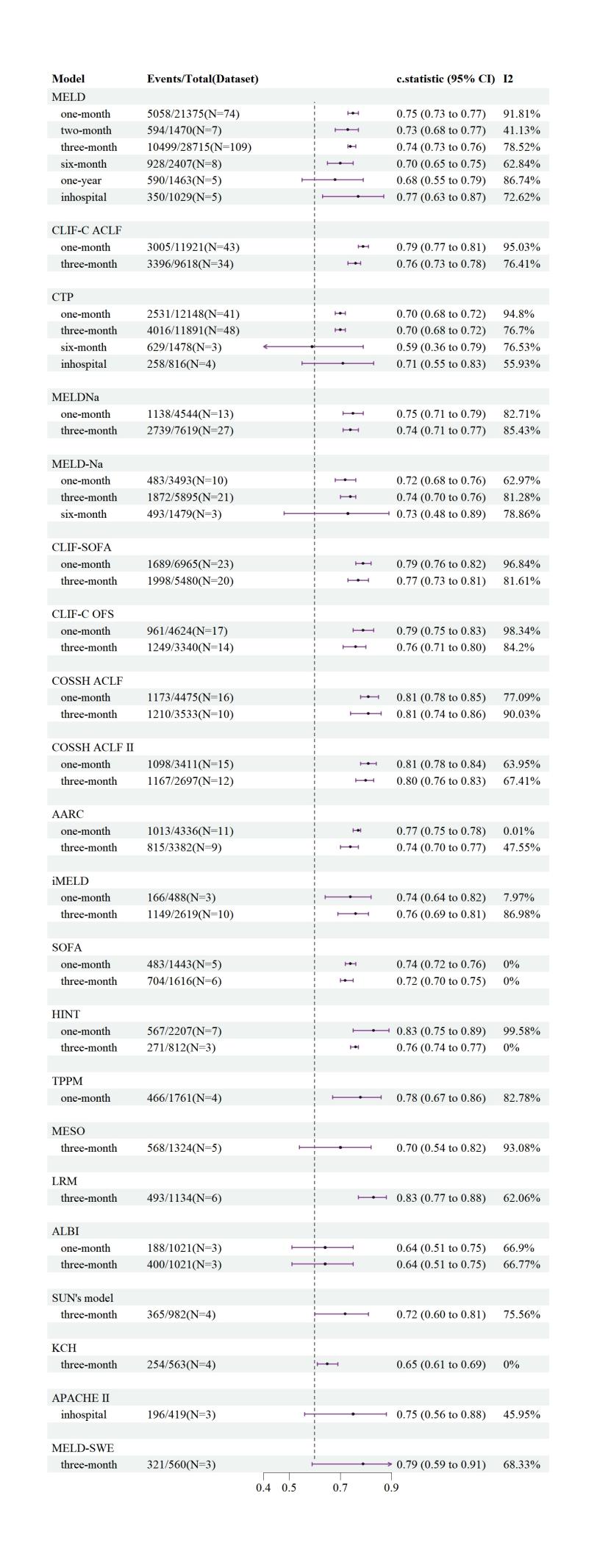


Figure 5. Forest plot of meta-analysis for prognostic prediction models in acute-on-chronic liver failure patients defined by APASL criteria.


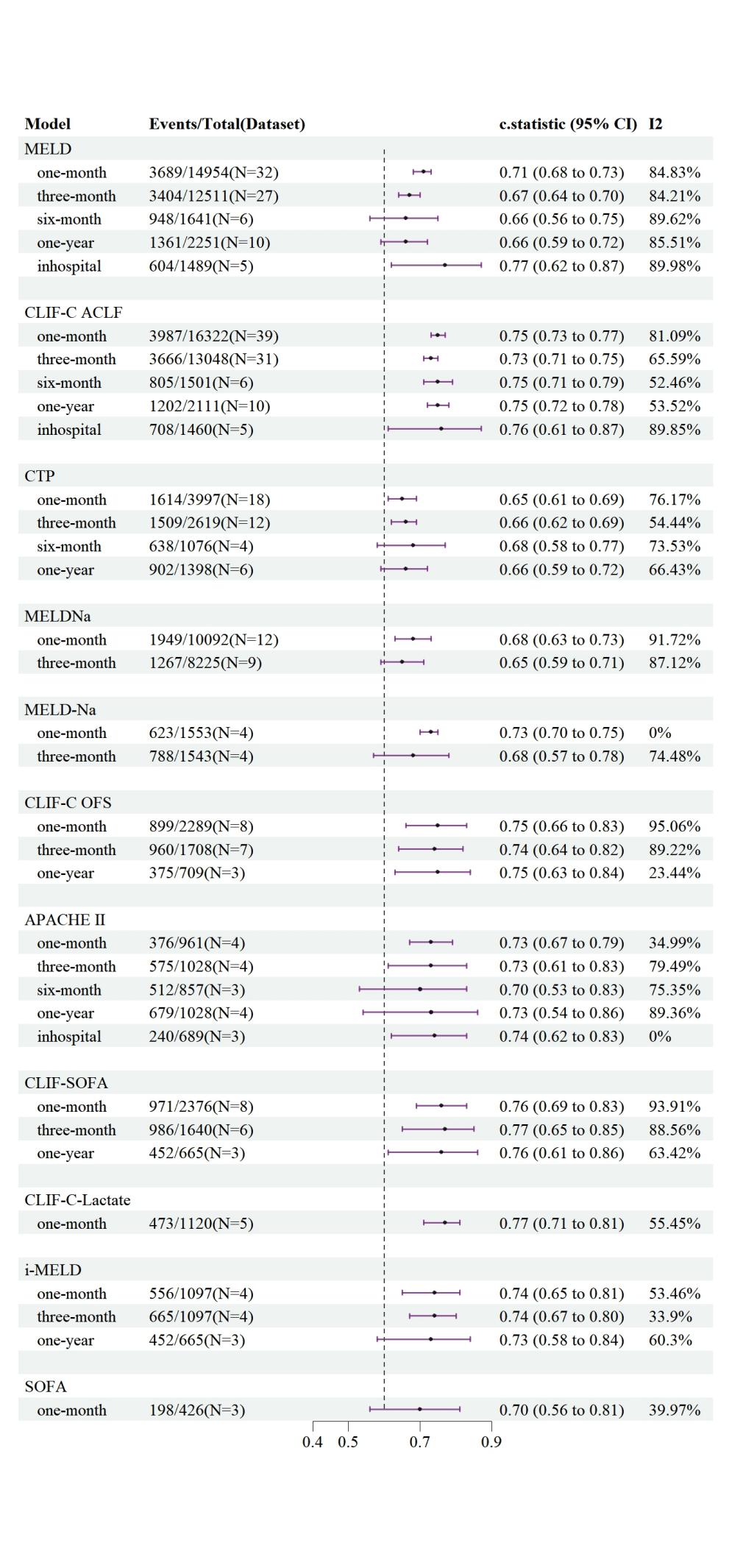


Figure 6. Forest plot of meta-analysis for prognostic prediction models in acute-on-chronic liver failure patients defined by EASL criteria.


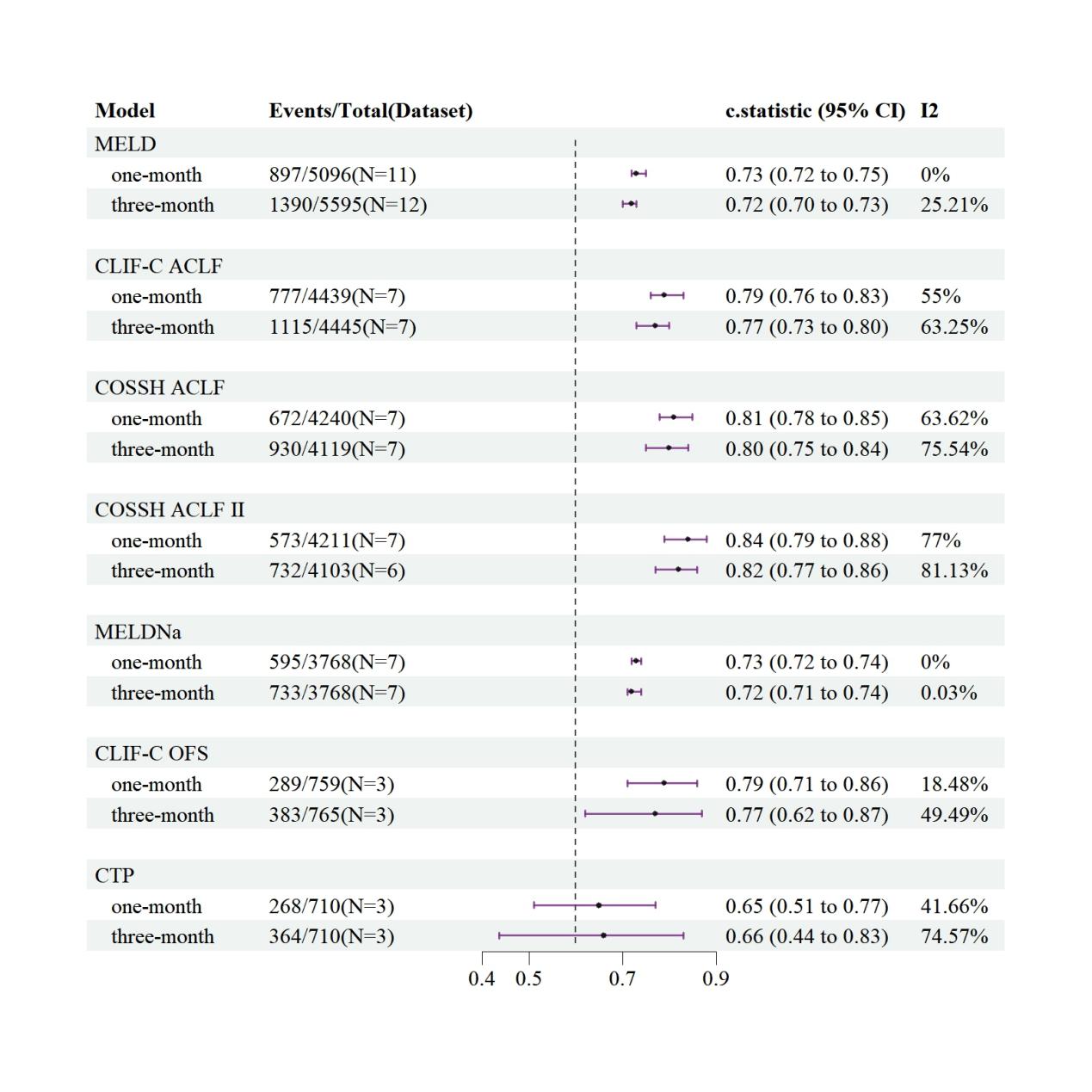


Figure 7. Forest plot of meta-analysis for prognostic prediction models in acute-on-chronic liver failure patients defined by COSSH criteria.


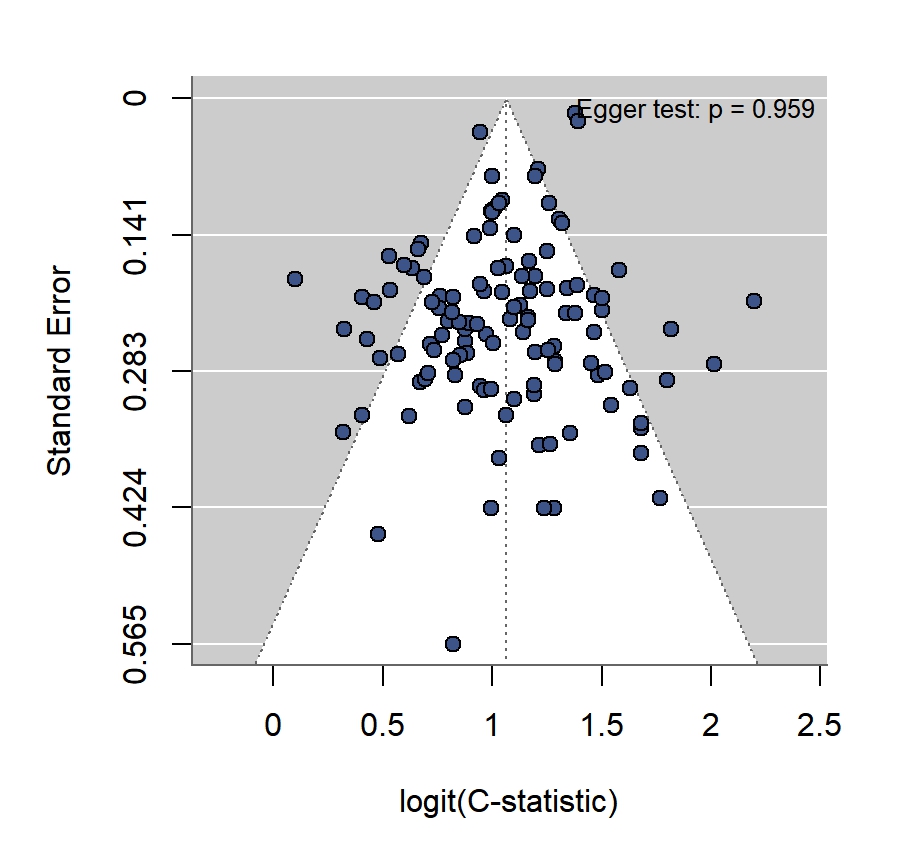


Figure 8. Funnel plot of the MELD model (one-month).


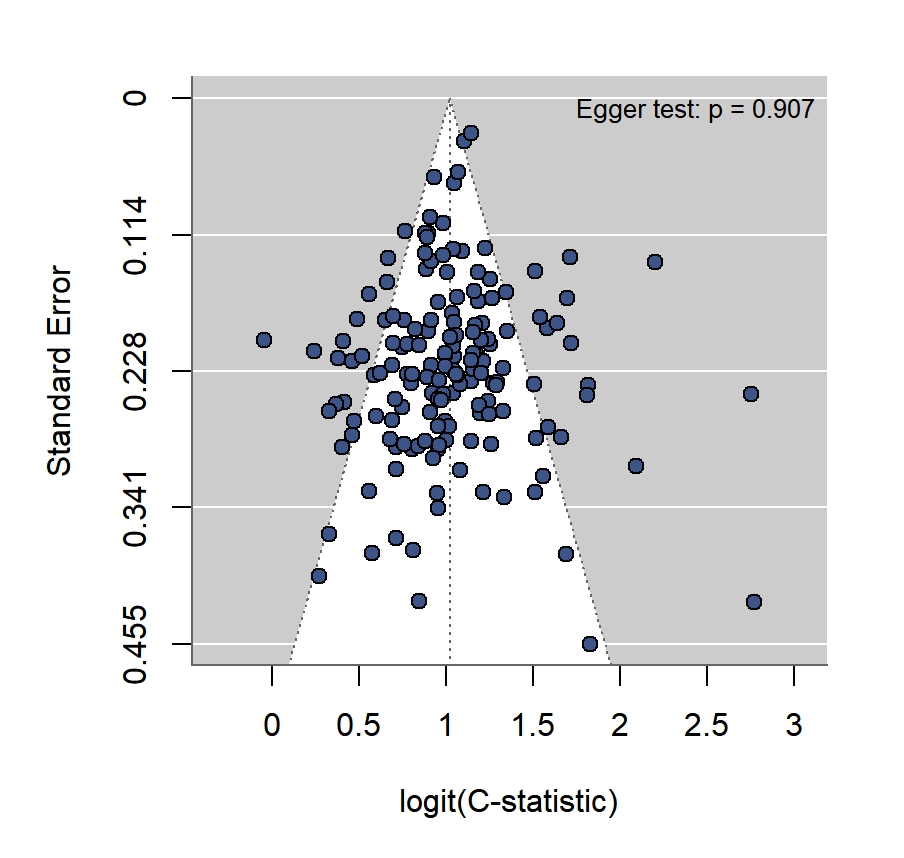


Figure 9. Funnel plot of the MELD model (three-month).


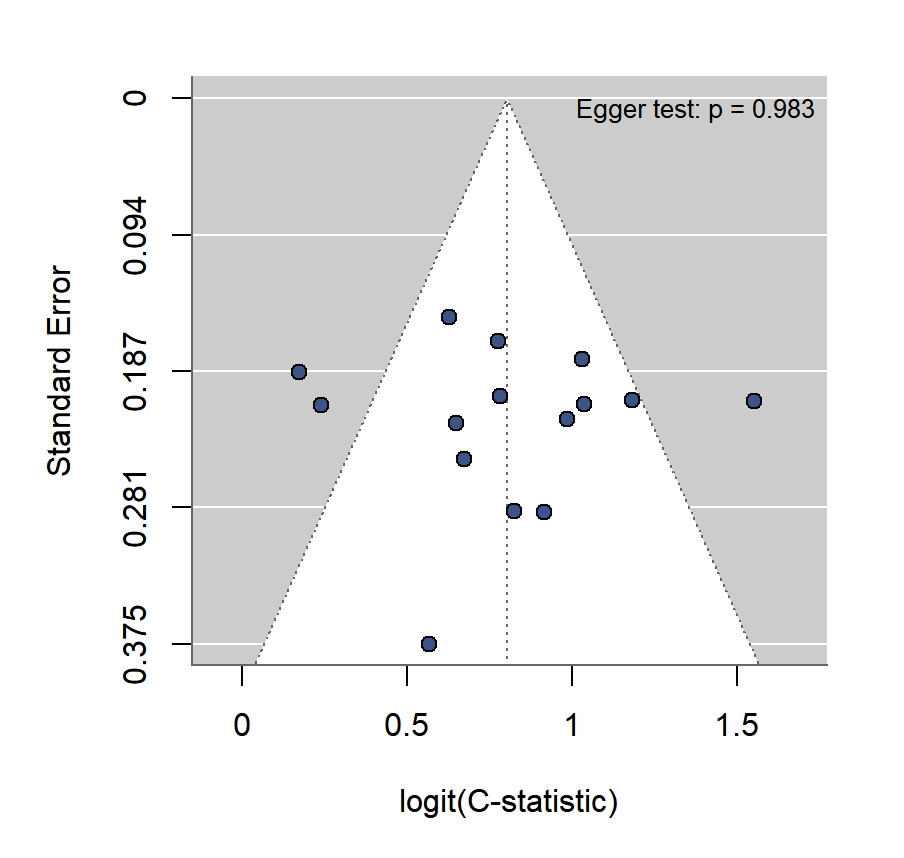


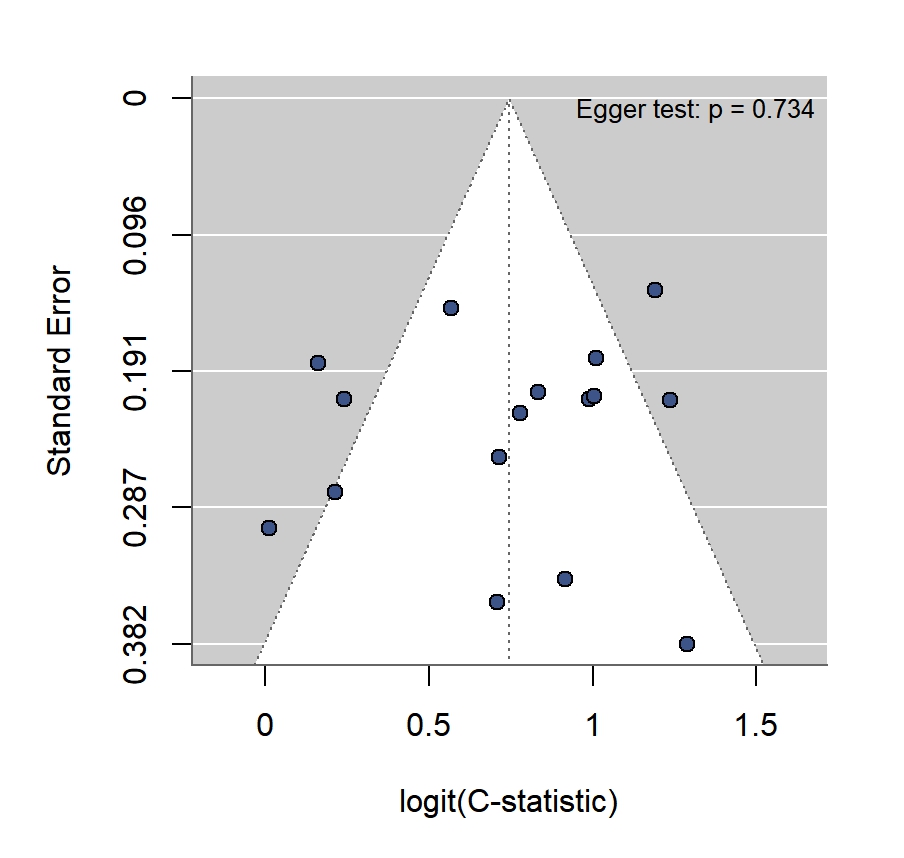
 Figure 10. Funnel plot of the MELD model (six-month).

Figure 11. Funnel plot of the MELD model (one-year).


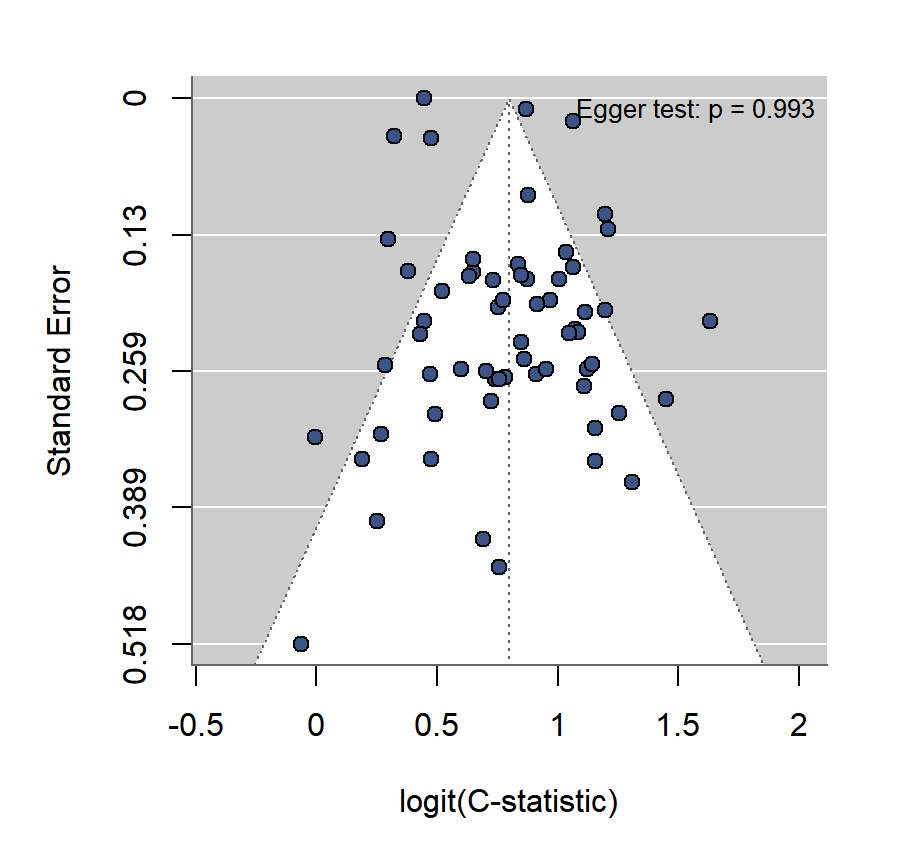


Figure 12. Funnel plot of the CTP model (one-month).


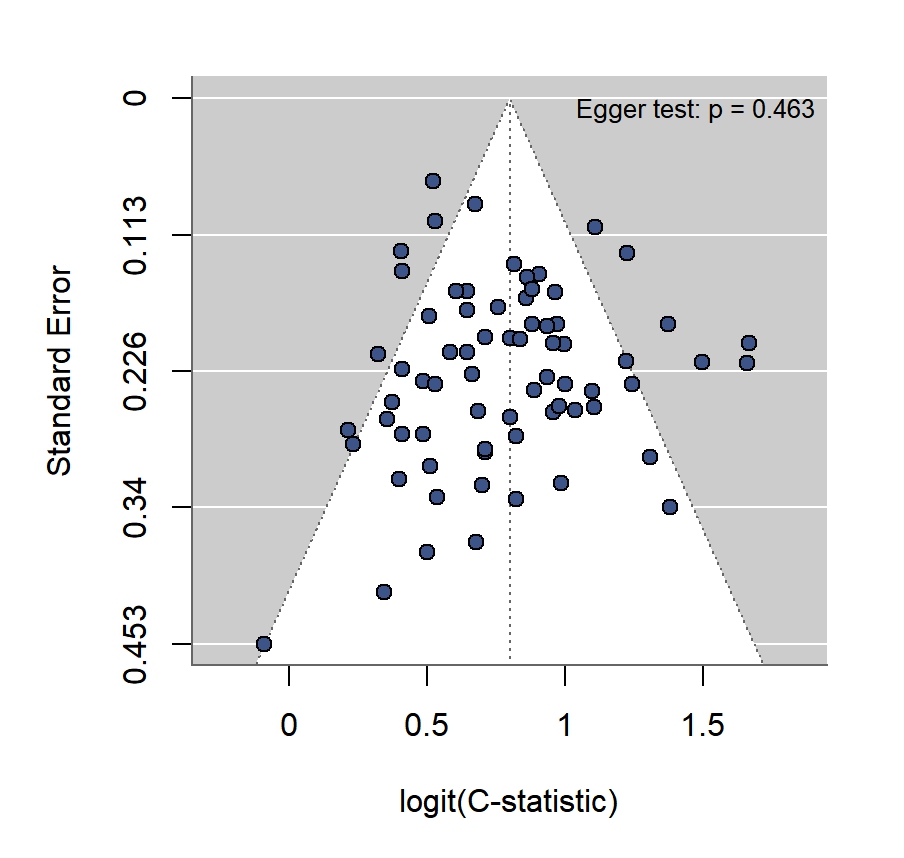


Figure 13. Funnel plot of the CTP model (three-month).


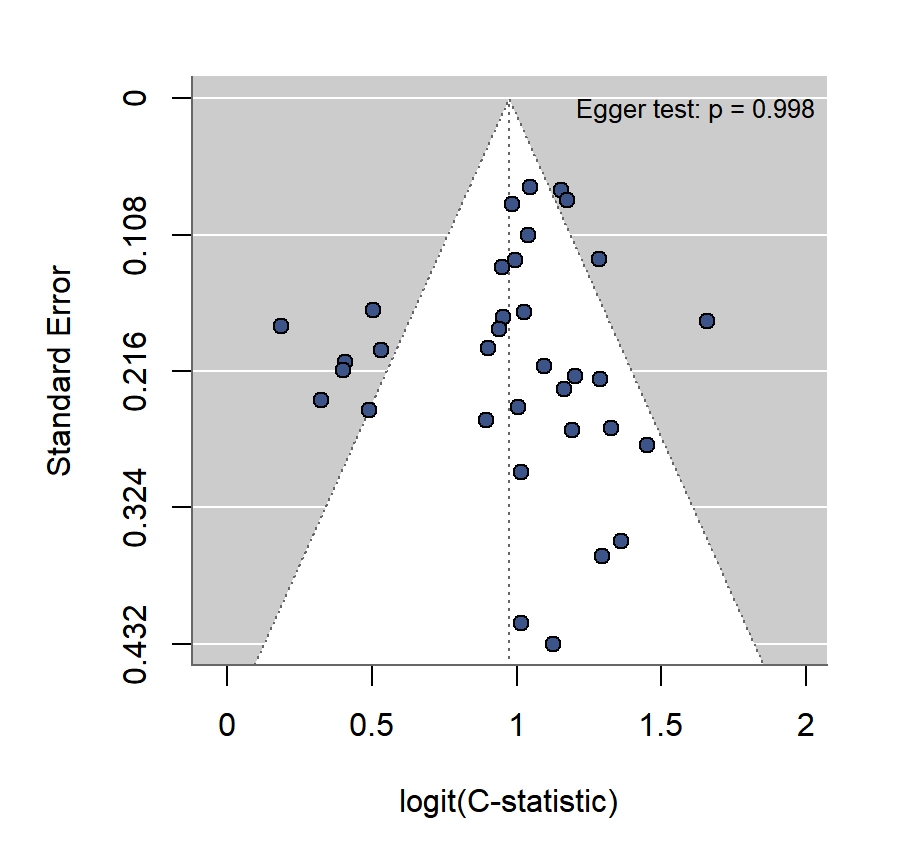


Figure 14. Funnel plot of the MELDNa model (one-month).


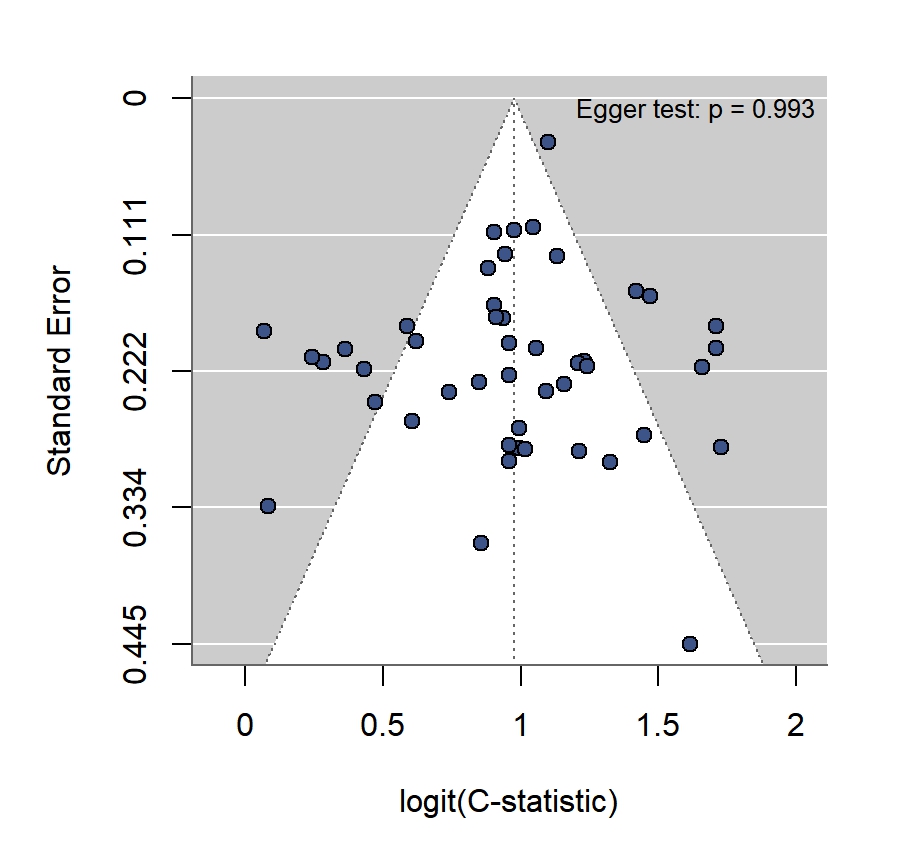


Figure 15. Funnel plot of the MELDNa model (three-month).


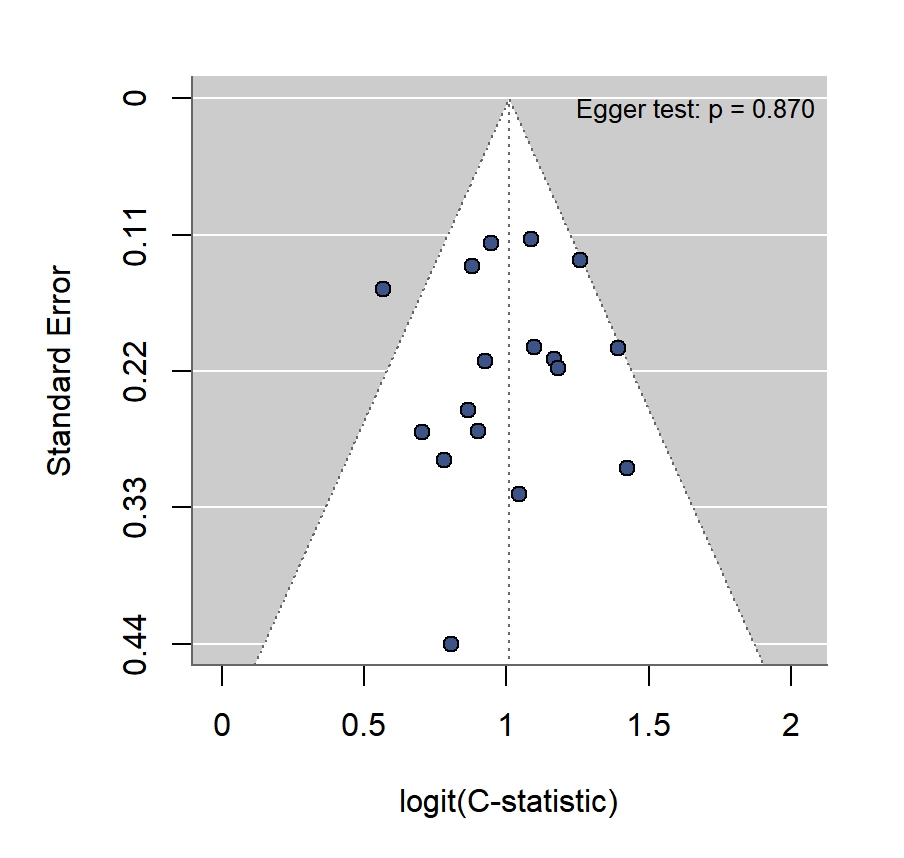


Figure 16. Funnel plot of the MELD-Na model (one-month).


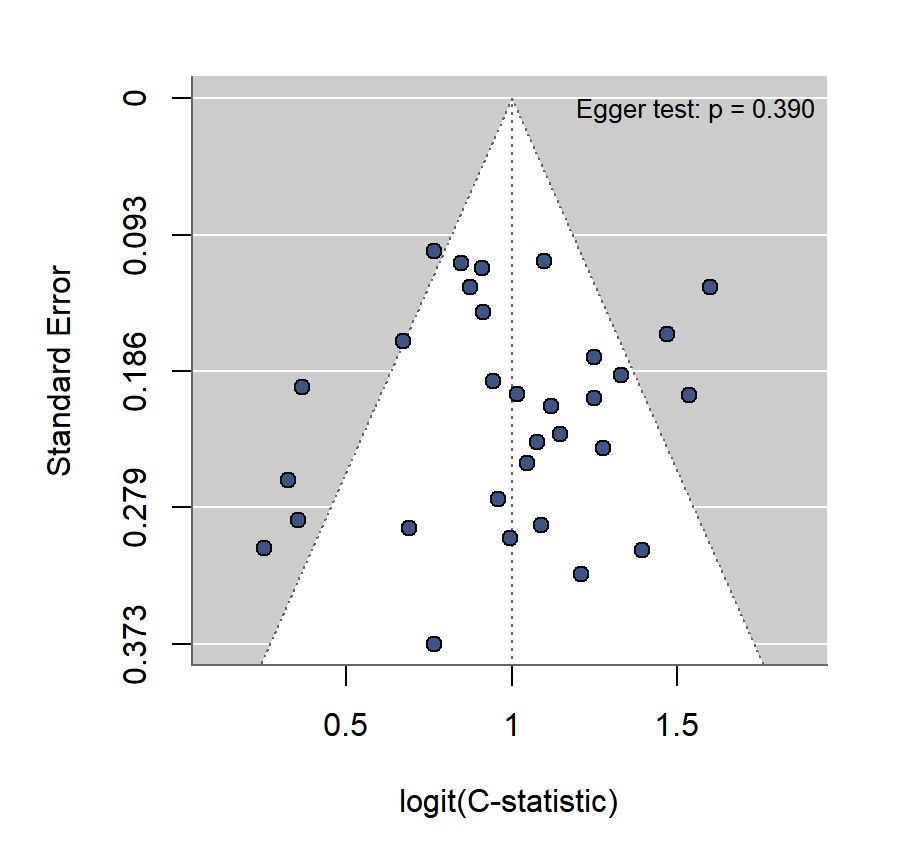


Figure 17. Funnel plot of the MELD-Na model (three-month).


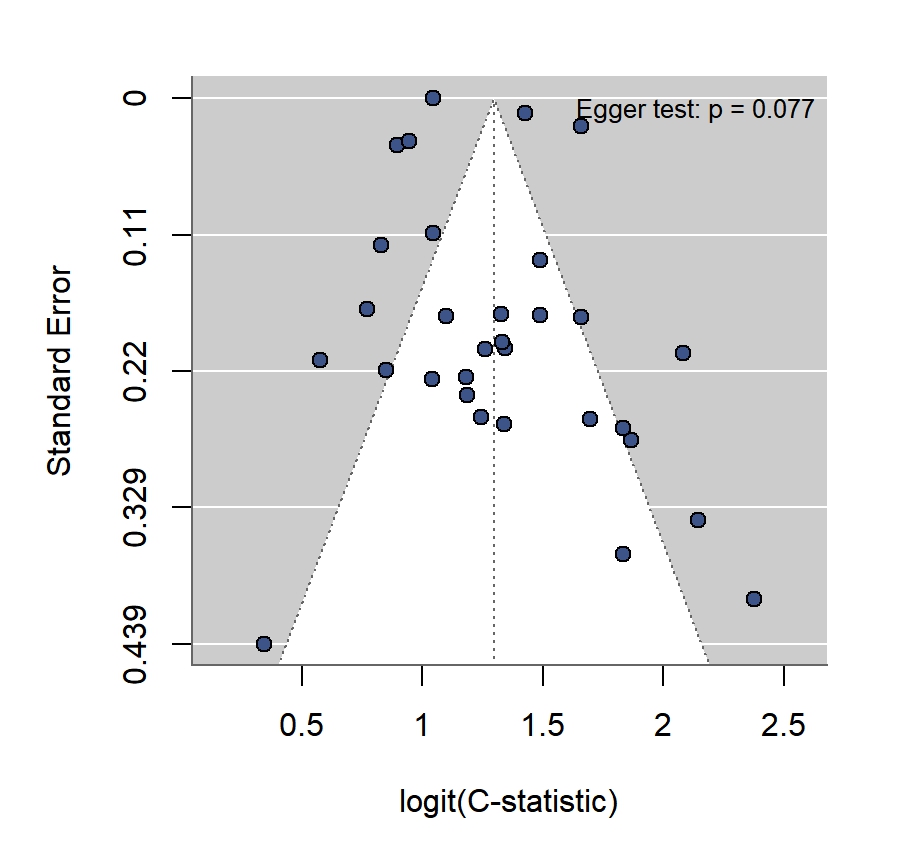


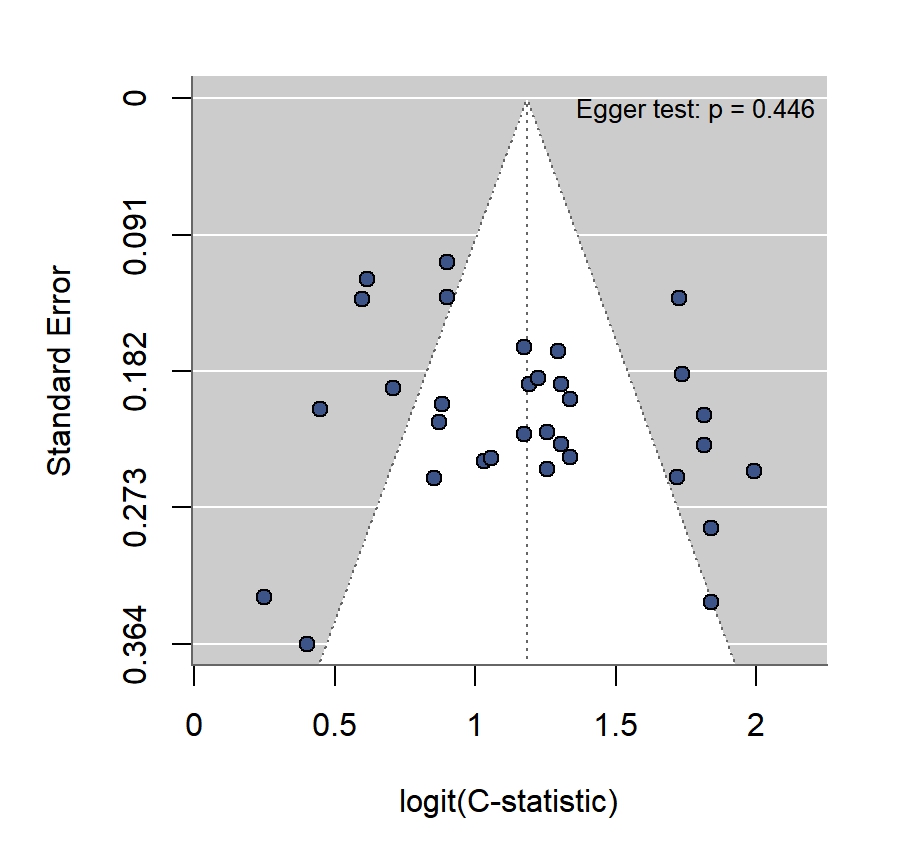
Figure 18. Funnel plot of the CLIF-SOFA model (one-month).

Figure 19. Funnel plot of the CLIF-SOFA model (three-month).


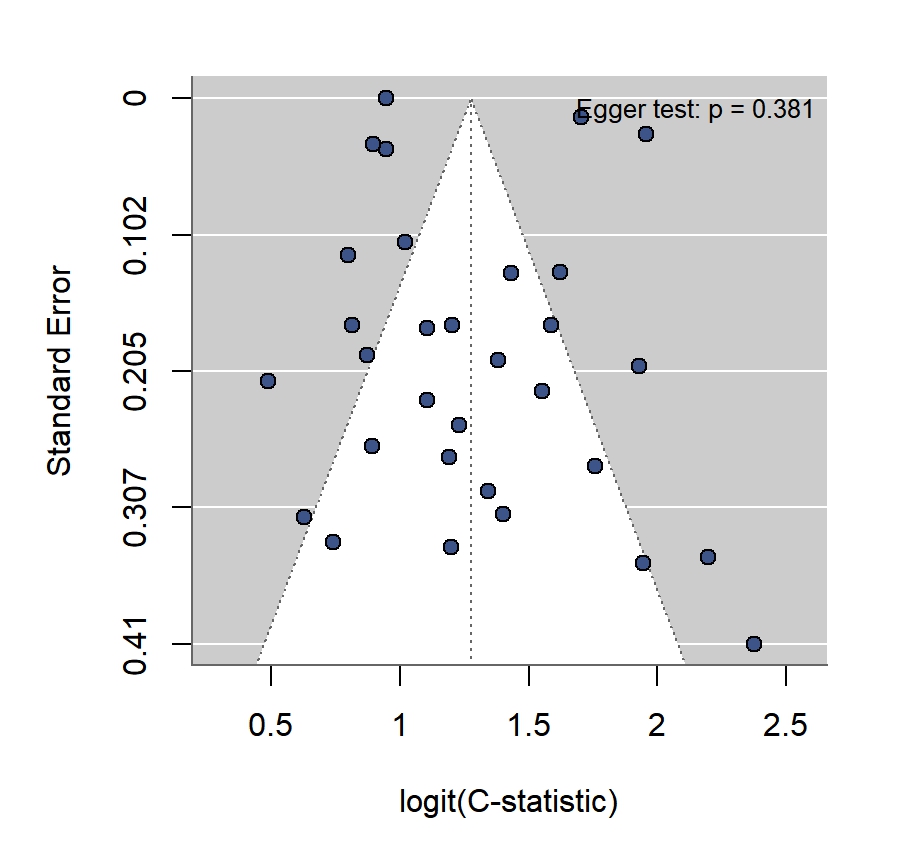


Figure 20. Funnel plot of the CLIF-C OFS model (one-month).


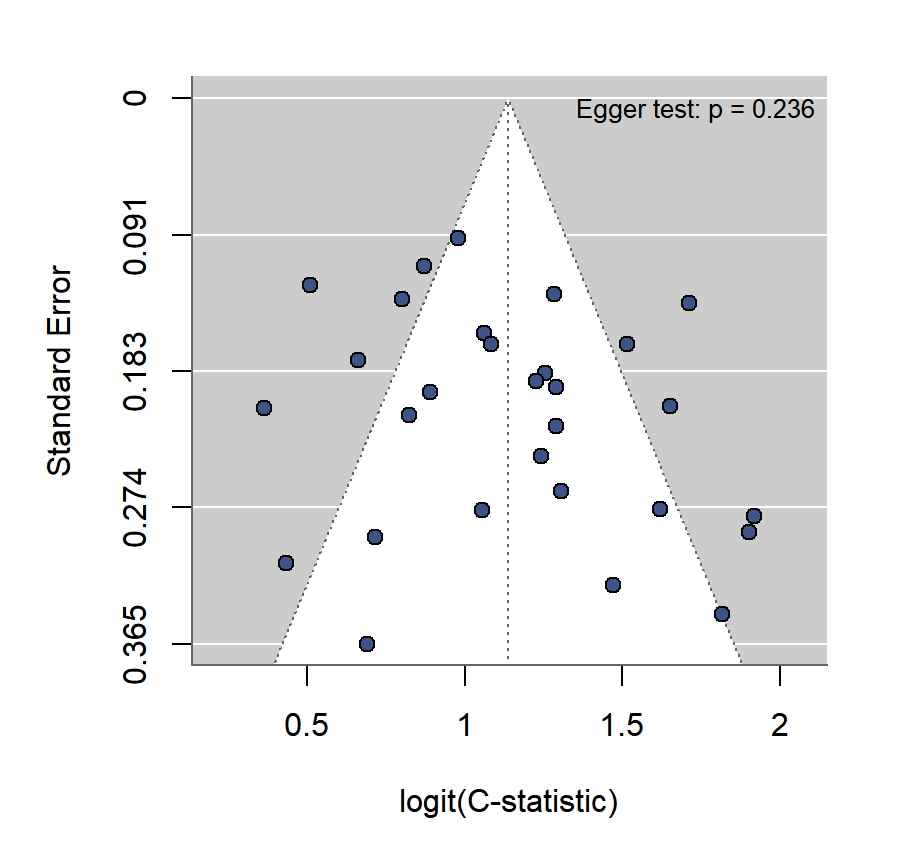


Figure 21. Funnel plot of the CLIF-C OFS model (three-month).


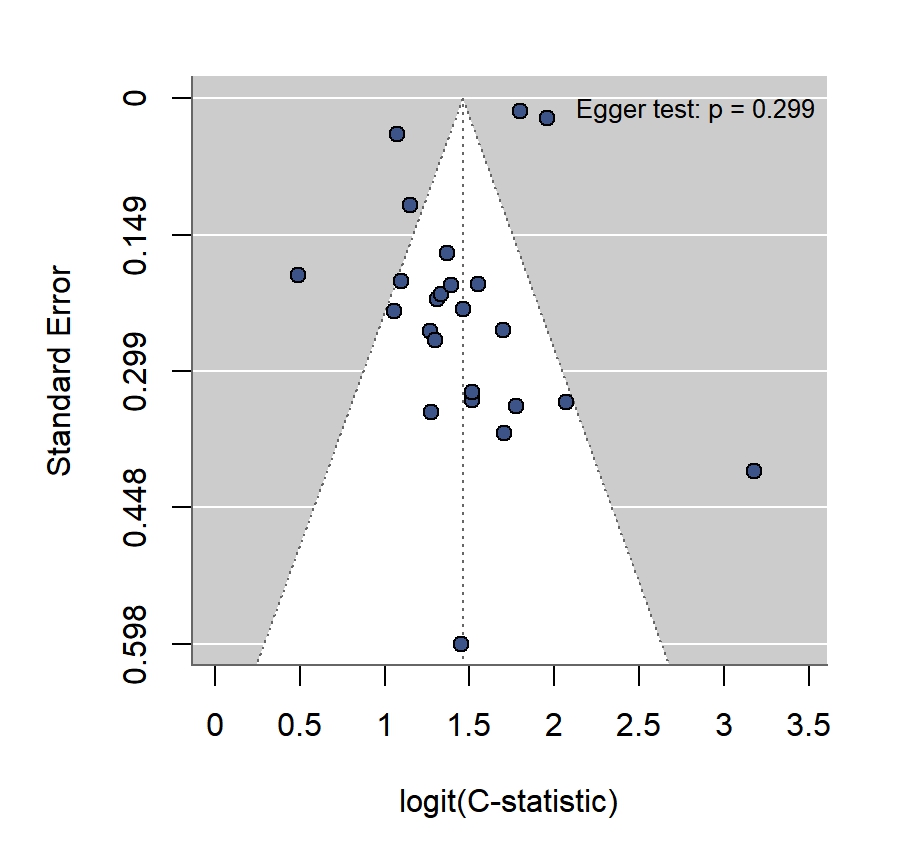


Figure 22. Funnel plot of the COSSH ACLF model (one-month).


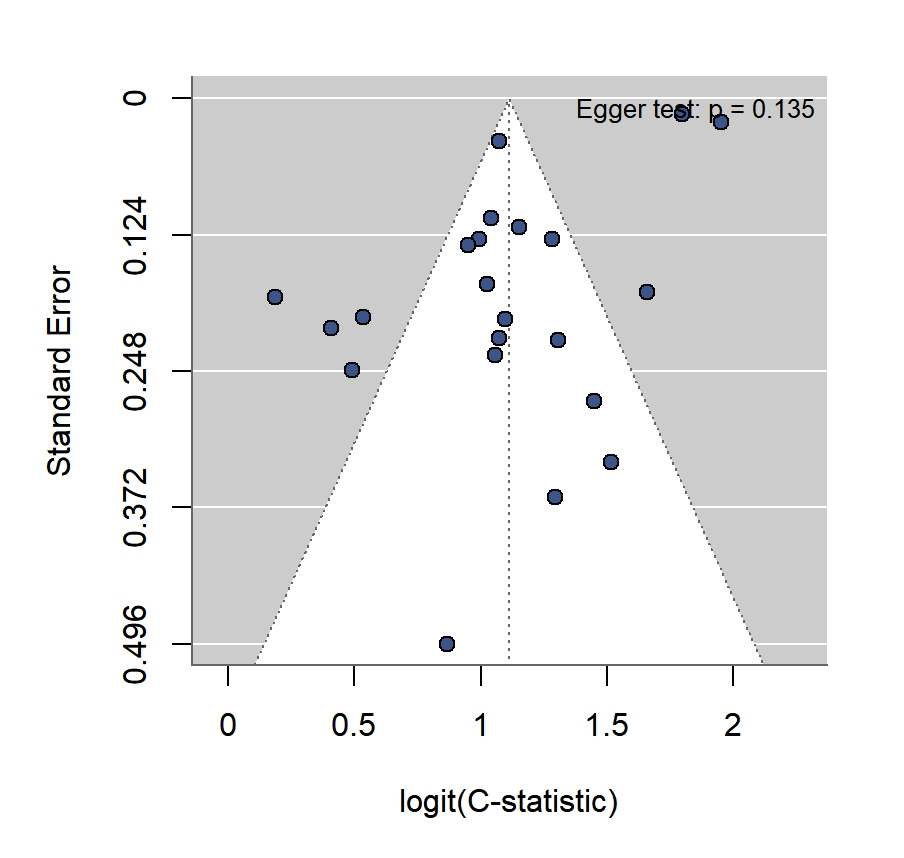


Figure 23. Funnel plot of the COSSH ACLF model (three-month).


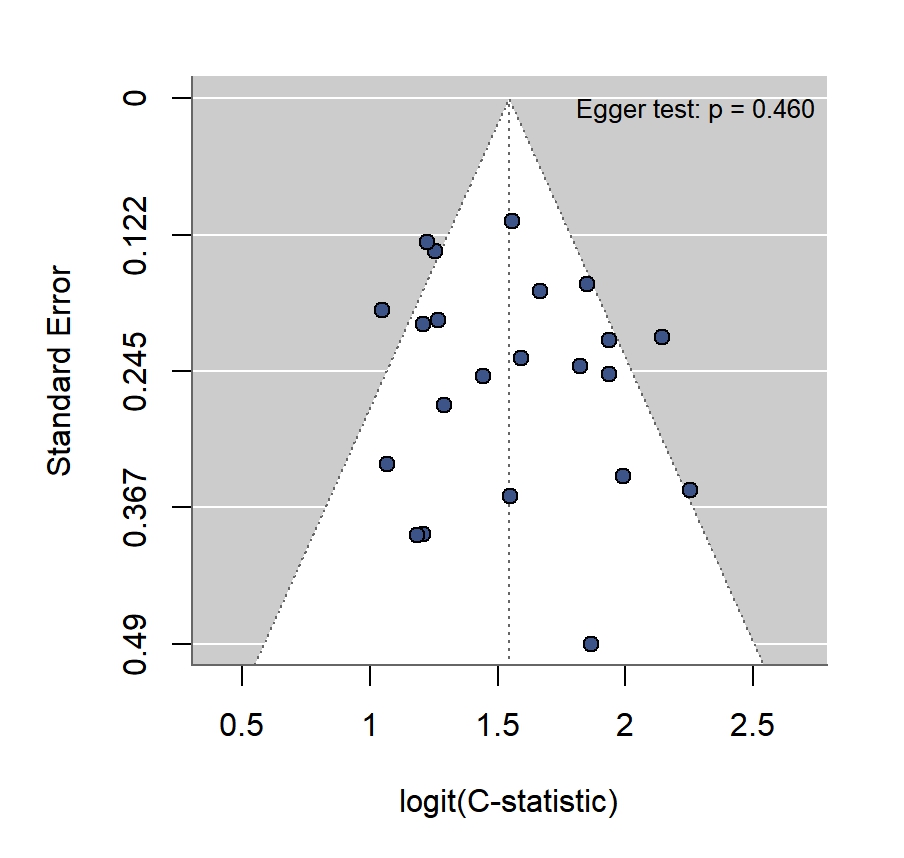


Figure 24. Funnel plot of the COSSH ACLF Ⅱ model (one-month).


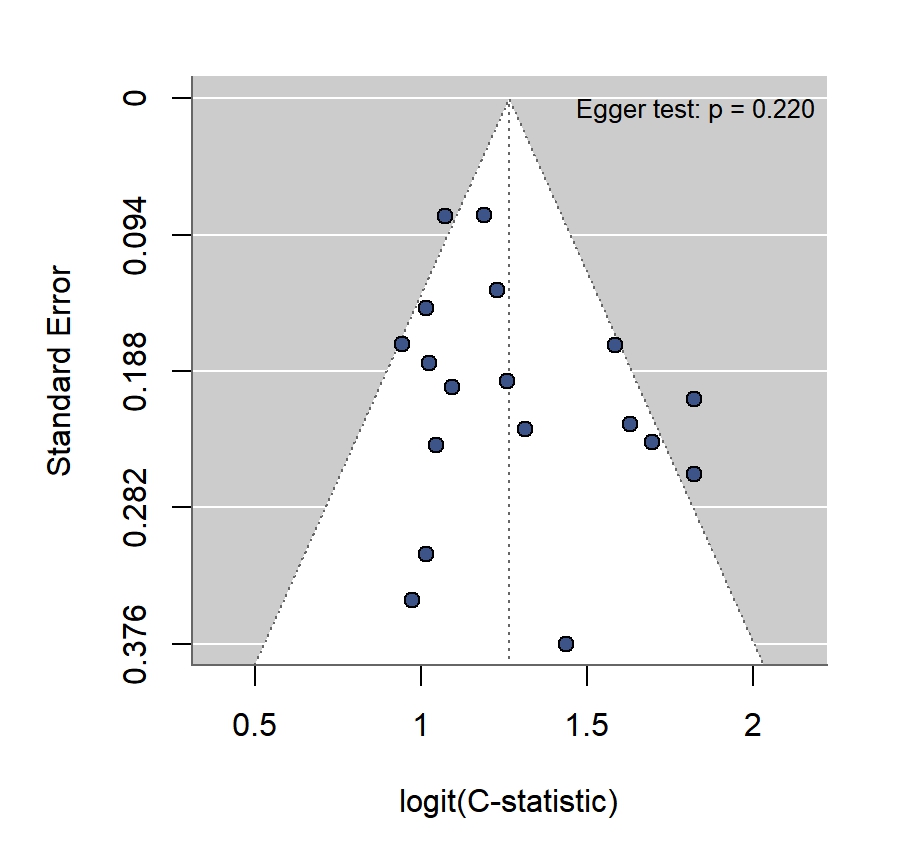


Figure 25. Funnel plot of the COSSH ACLF Ⅱ model (three-month).


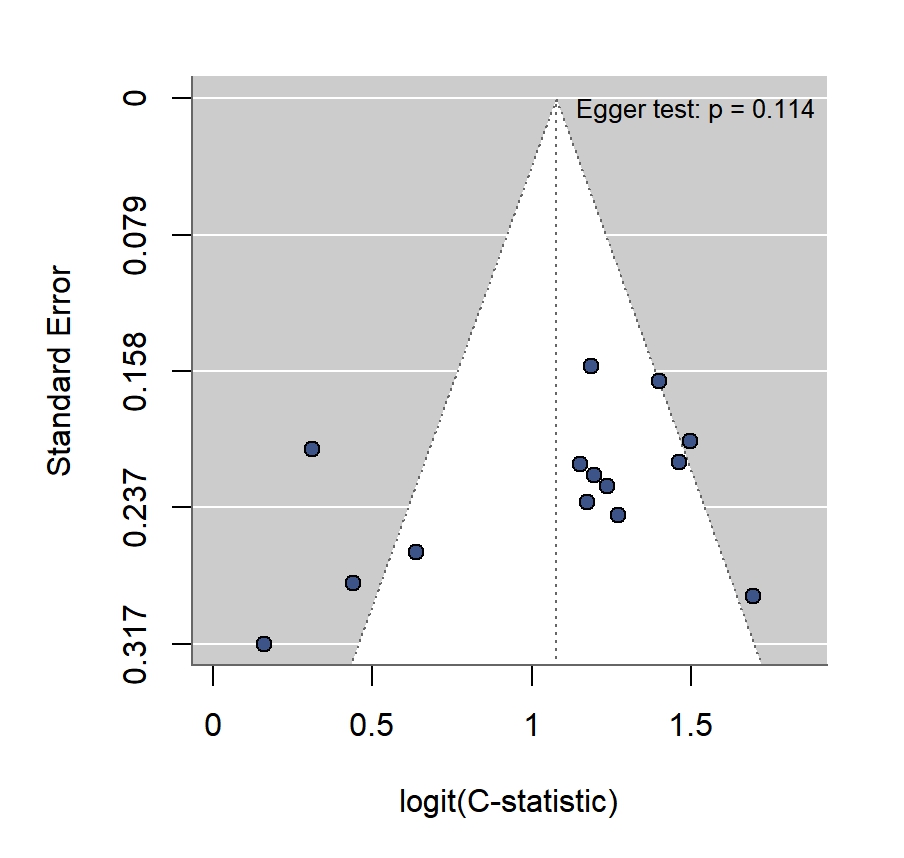


Figure 26. Funnel plot of the iMELD model (three-month).


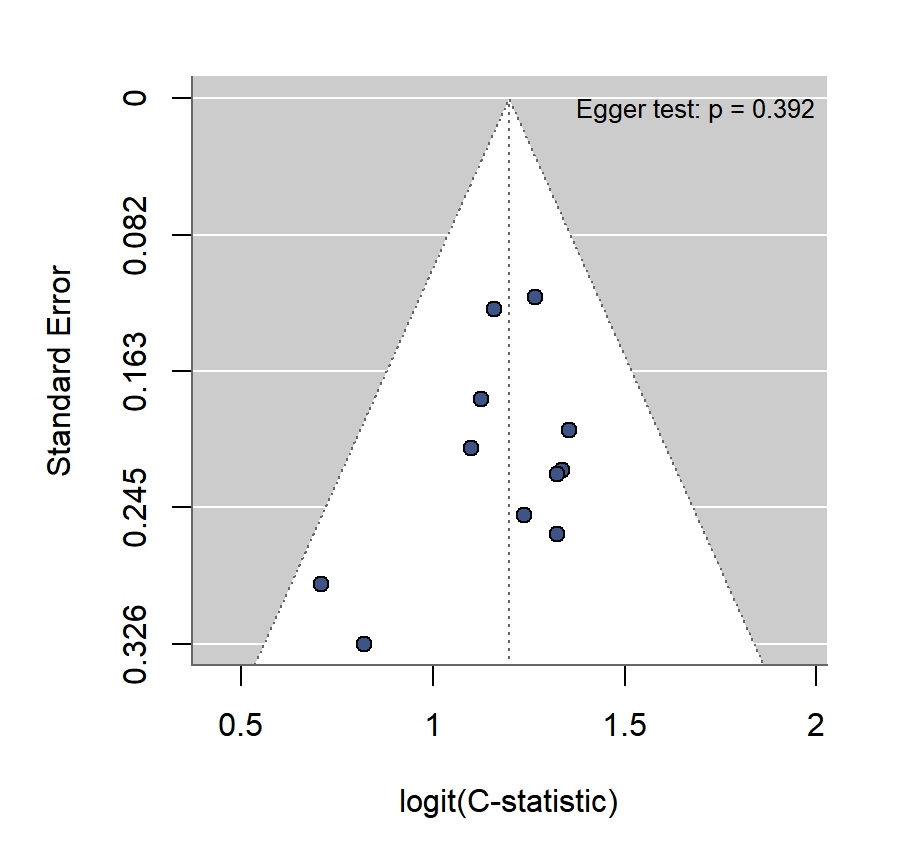


Figure 27. Funnel plot of the AARC model (one-month).


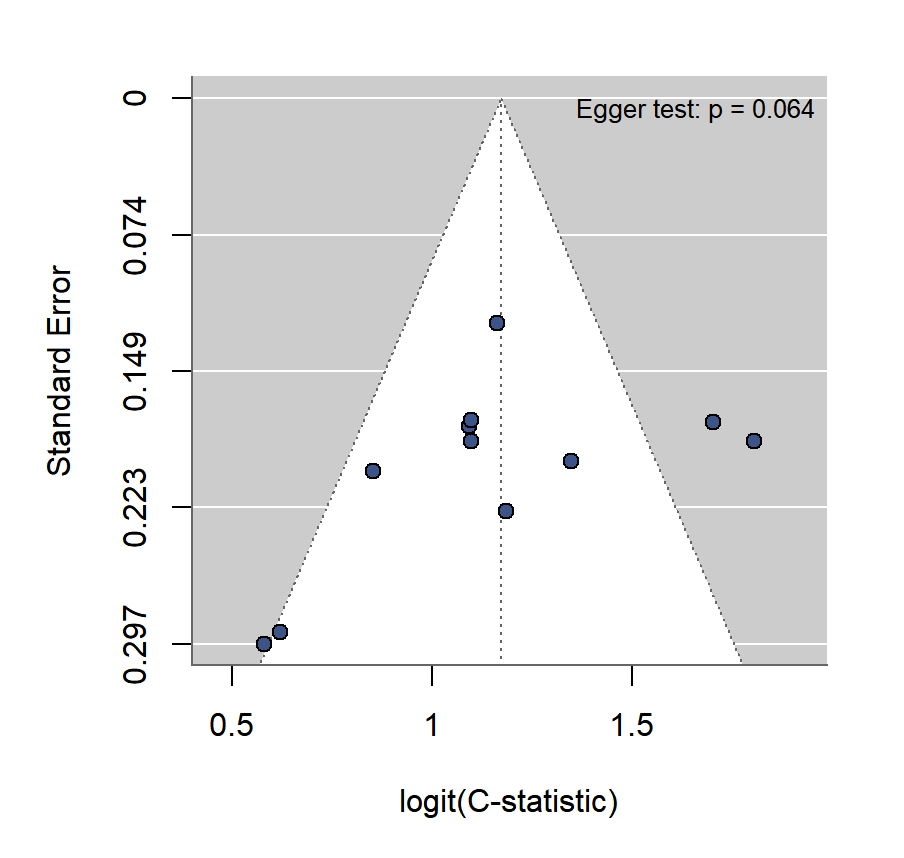


Figure 28 Funnel plot of the AARC model(three-month)
