# Supplementary material for: Prediction models for mortality in patients with acute on chronic liver failure: systematic review and critical appraisal
Source: Front Med (Lausanne). 2026 Jun 16;13:1829188. doi: 10.3389/fmed.2026.1829188 (PMC13314772; doi:10.3389/fmed.2026.1829188)
Supplement: Supplementary file 5 [file Table_5.DOCX]

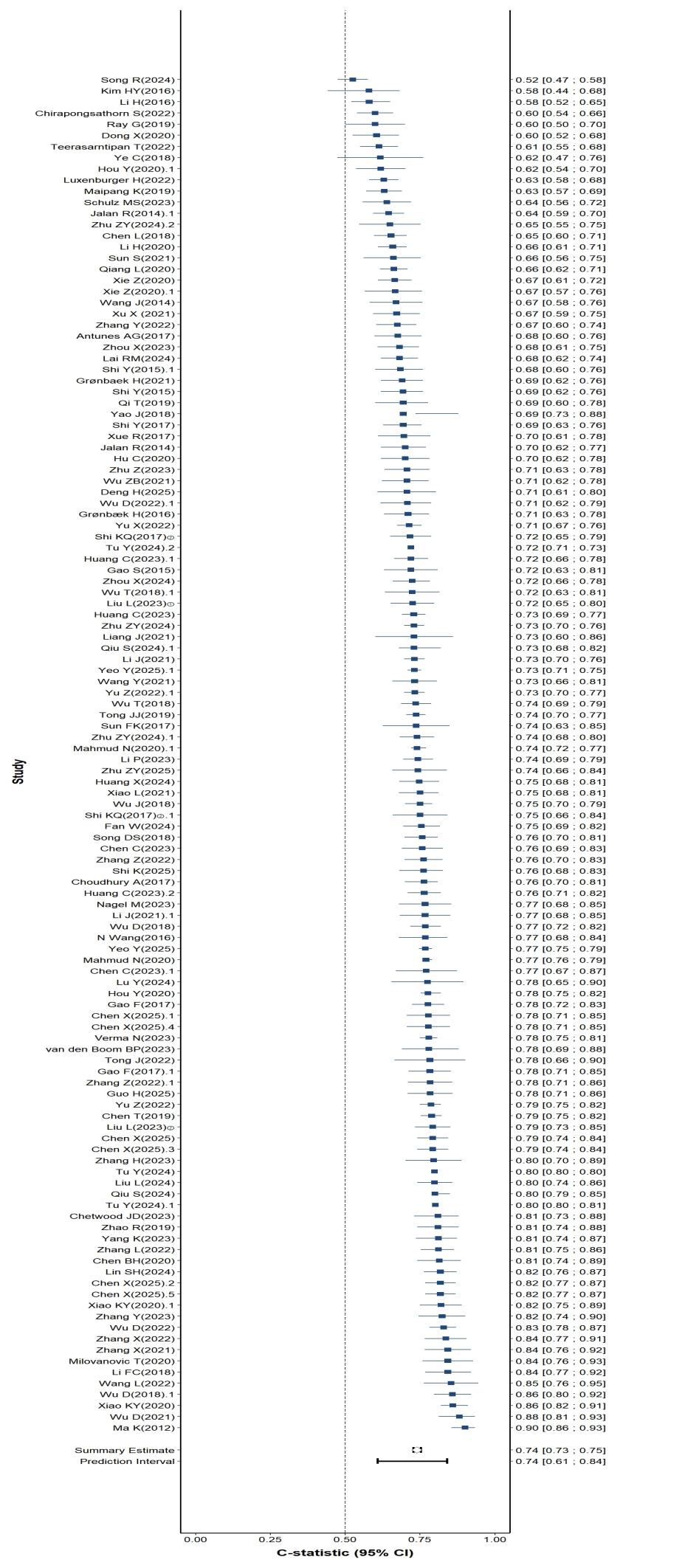


Note: Q= 1164.18 (df = 121, p <0.0001); I²=91.21%; τ² = 0.0934.

Figure 1. Forest plot of the MELD score for 1-month mortality.


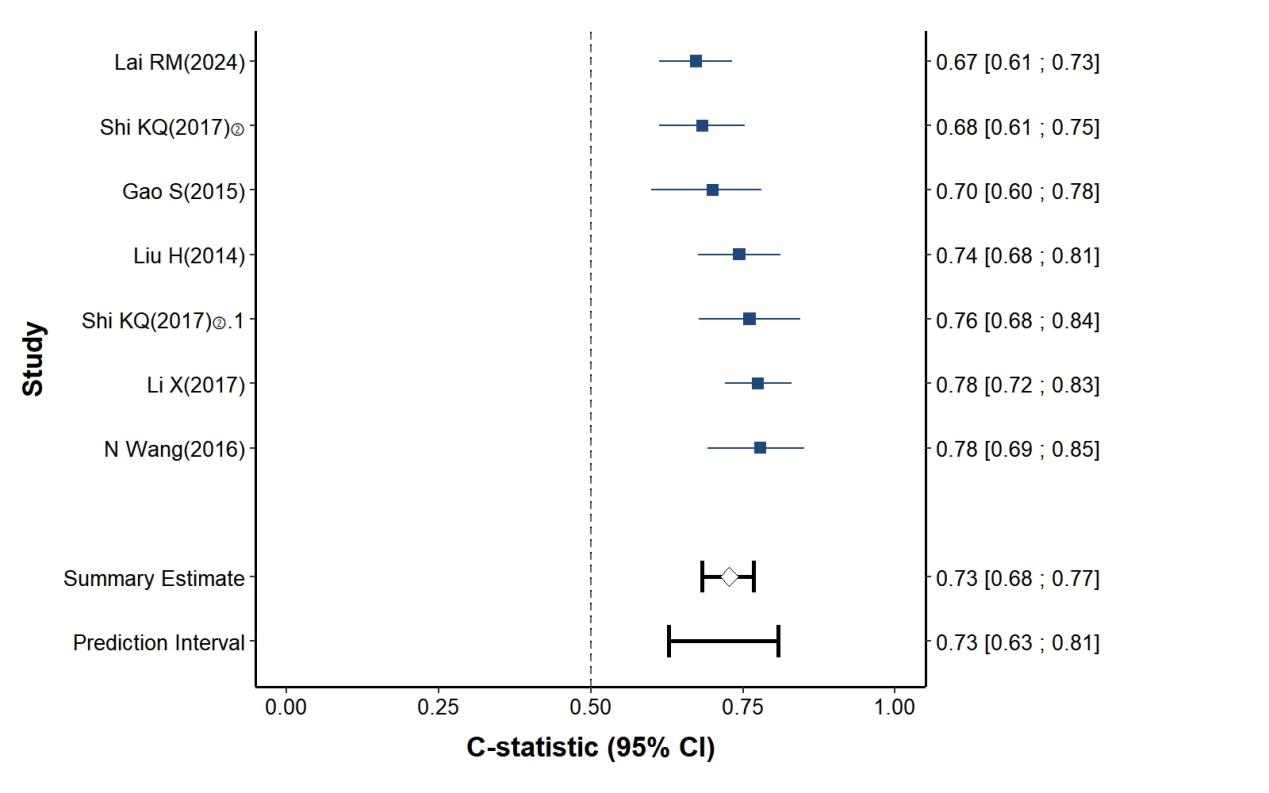


Note: Q = 9.92 (df =6, p =0.1281); I²=41.13%; τ²=0.024.

Figure 2. Forest plot of the MELD score for 2-month mortality.


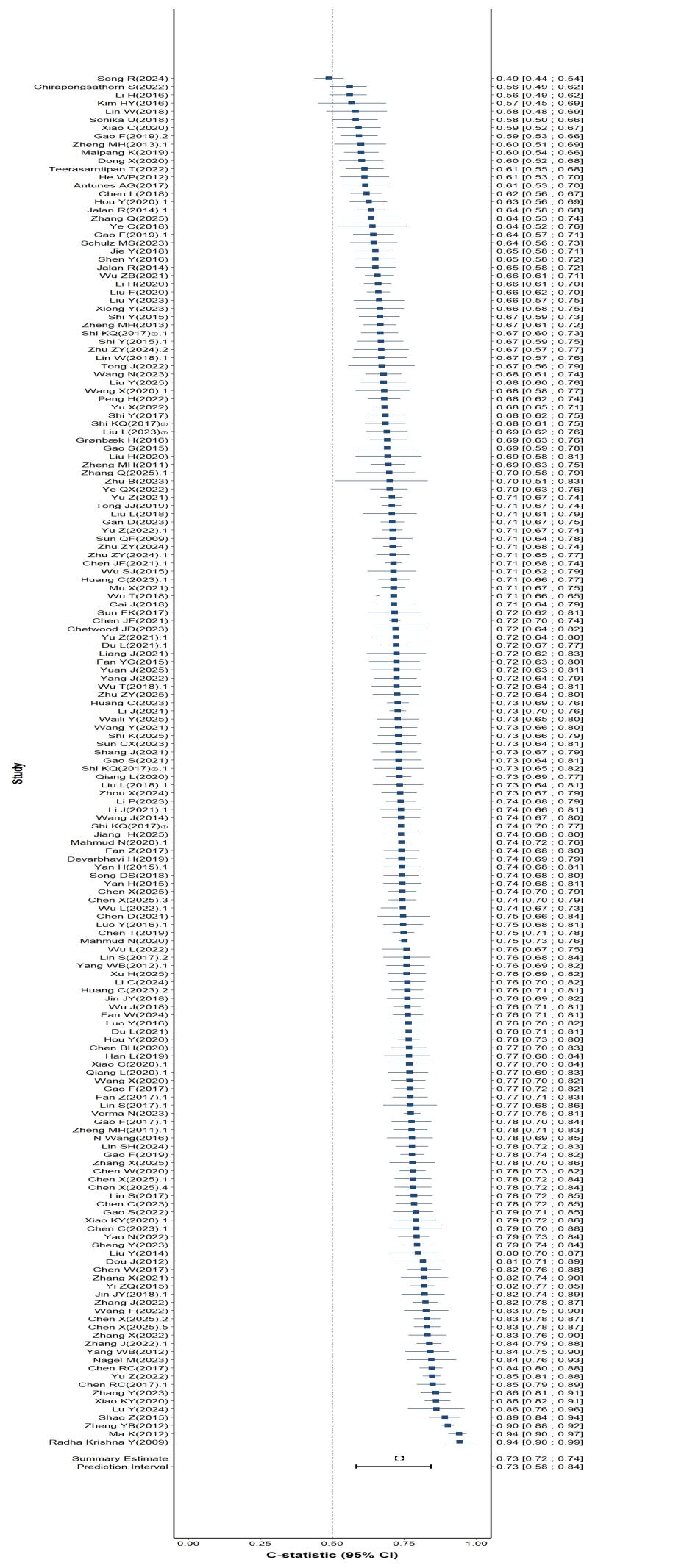


Note: Q =927.53 (df =166, p <0.0001); I²=88.76%; τ²= 0.1131.

Figure 3. Forest plot of the MELD score for 3-month mortality.


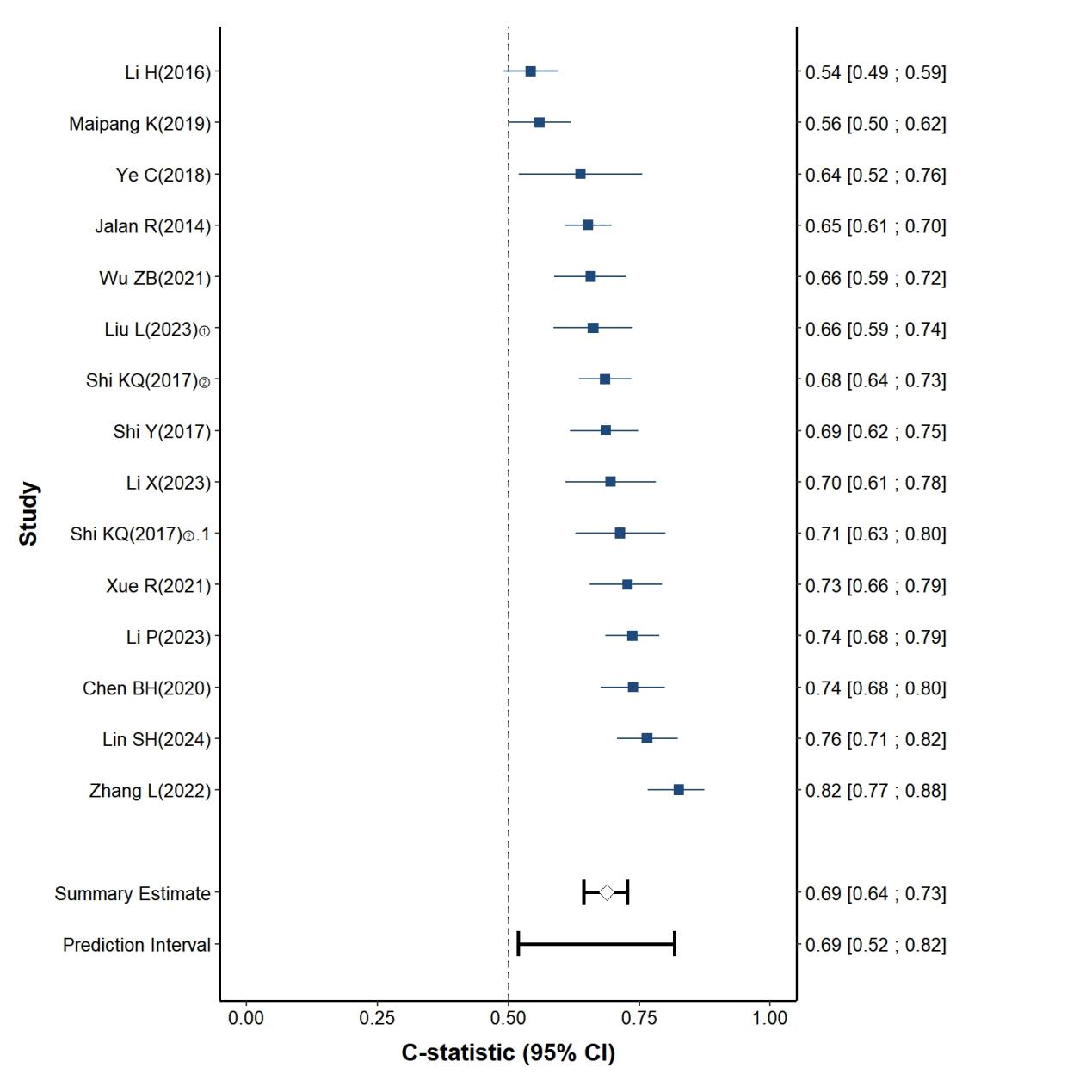


Note: Q =80.48 (df =14, p <0.0001); I²= 81.51%; τ² = 0.1002.

Figure 4. Forest plot of the MELD score for 6-month mortality.


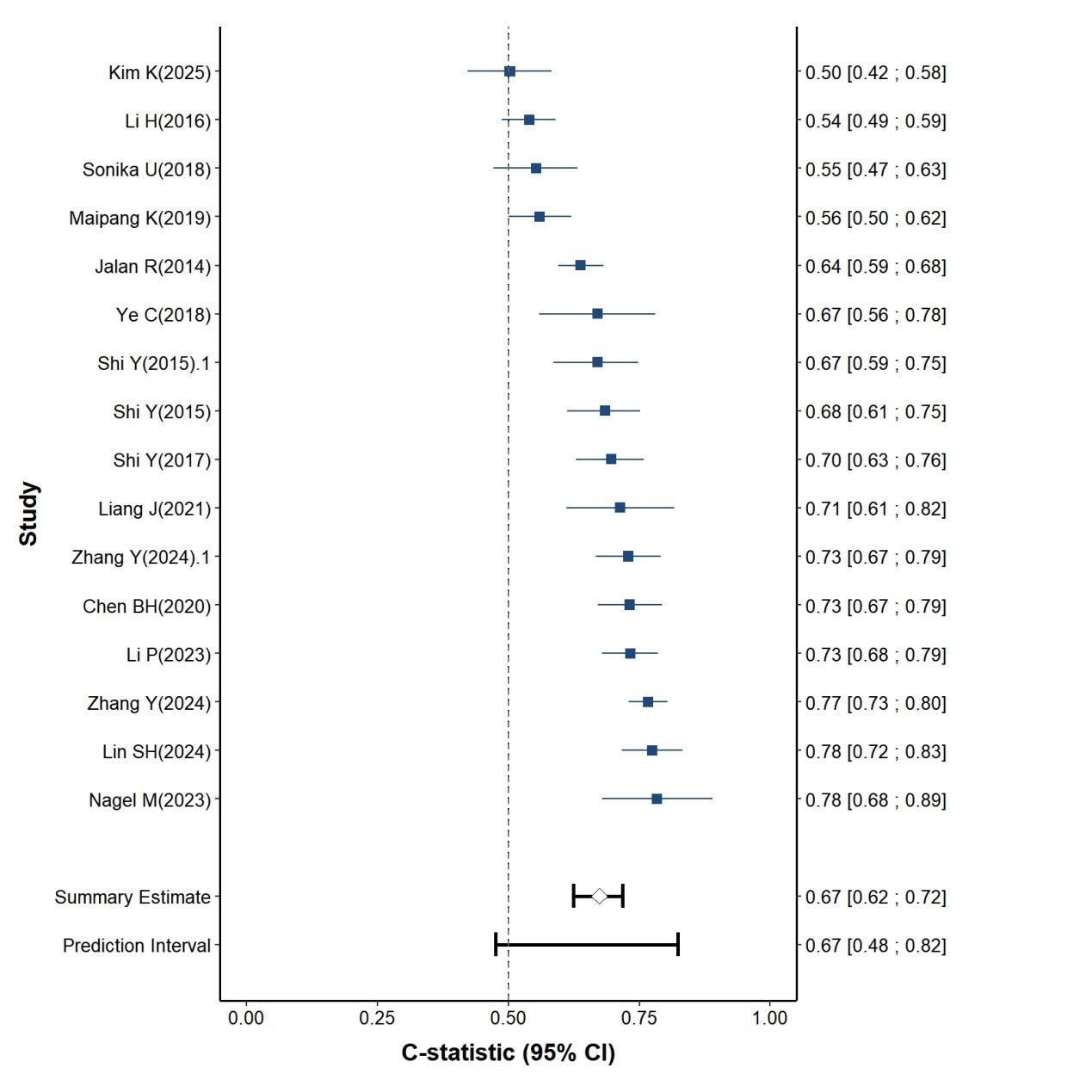


Note: Q =113.75 (df =15, p <0.0001); I²=85.70%; τ² = 0.1359 .

Figure 5. Forest plot of the MELD score for 1-year mortality.


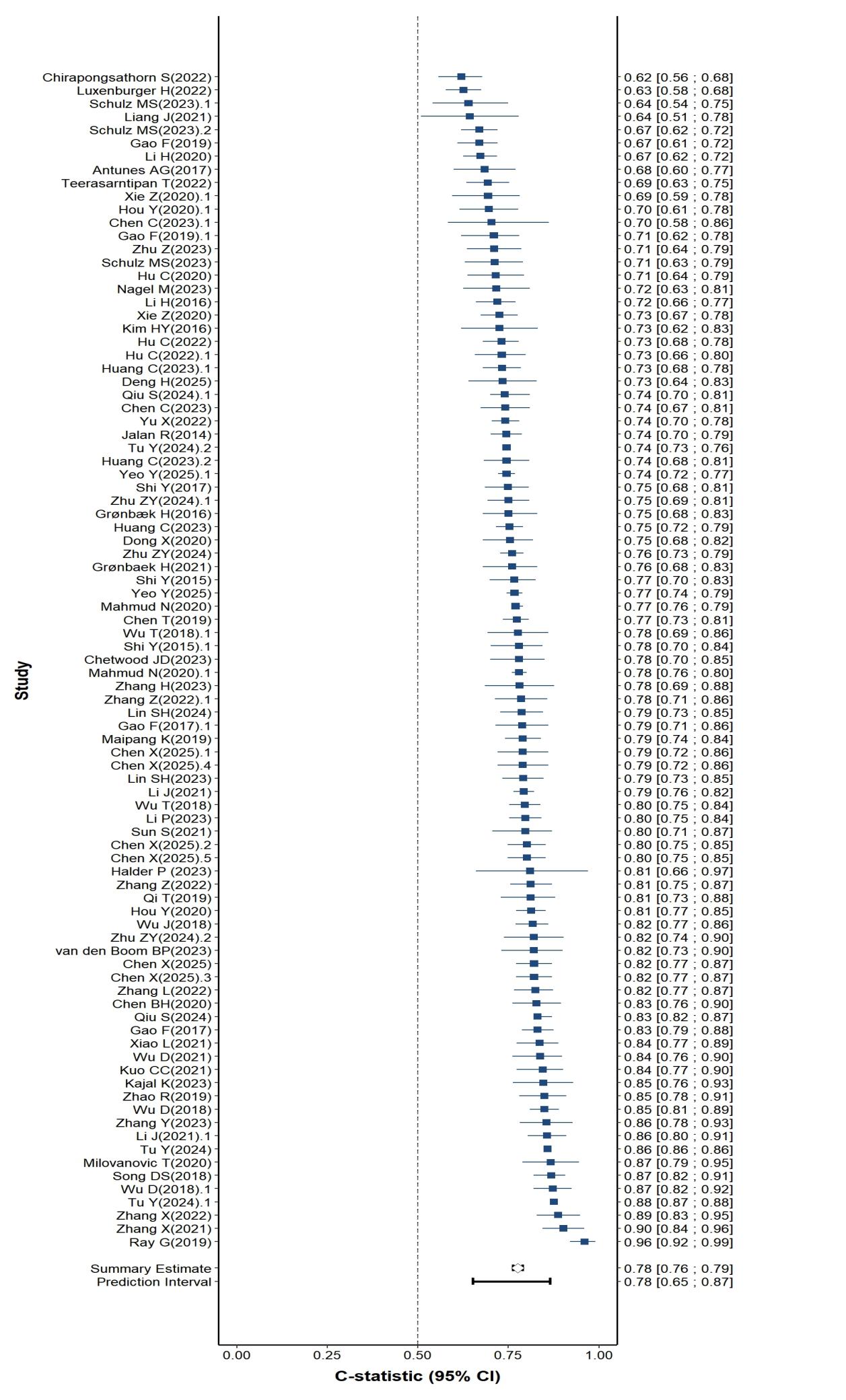


Note: Q =2140.98 (df =88, p <0.0001); I²=92.75%; τ² =0.0939.

Figure 6. Forest plot of the CLIF-C ACLF score for 1-month mortality.


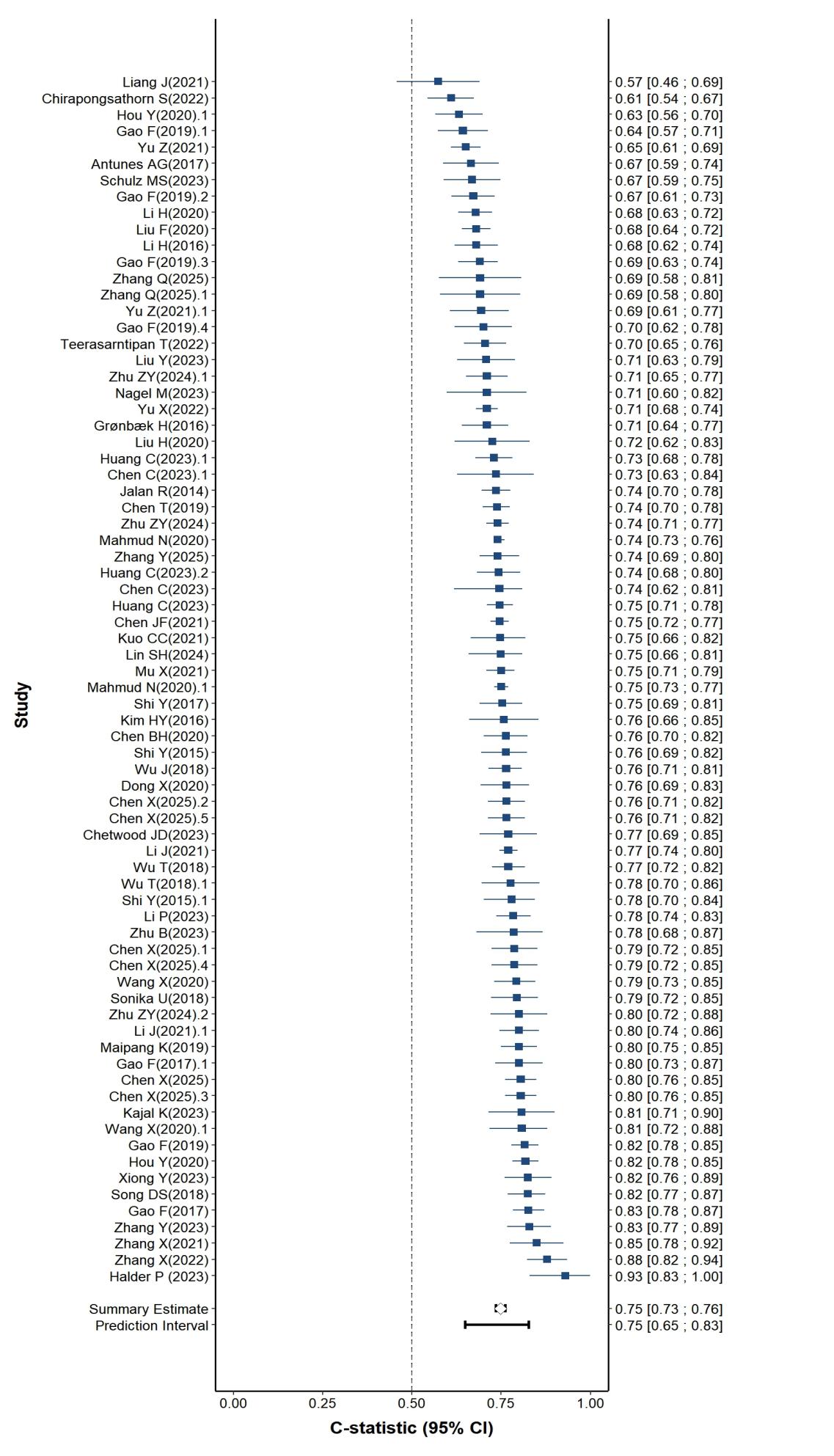


Note: Q =242.41 (df =73, p <0.0001); I²=75.48%; τ² =0.0556.

Figure 7. Forest plot of the CLIF-C ACLF score for 3-month mortality.


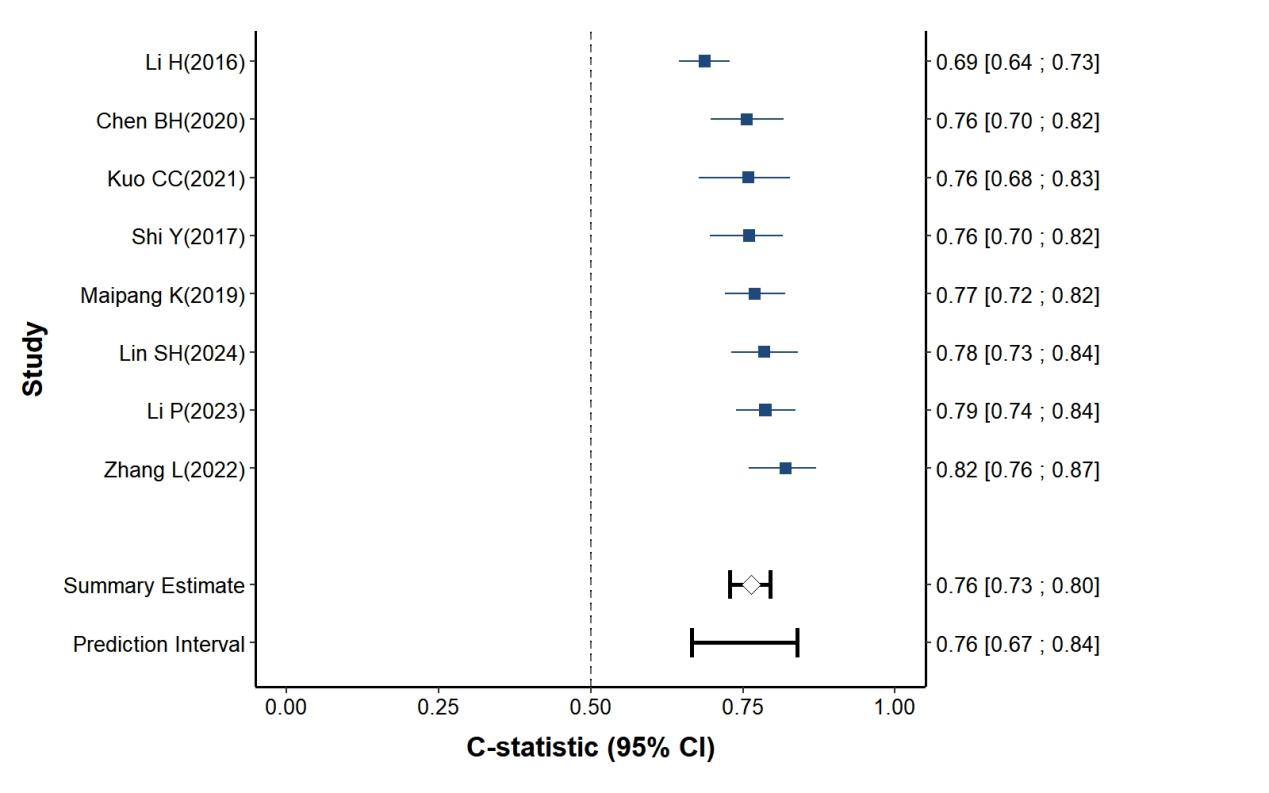


Note: Q =18.78 (df =7, p =0.0089); I²=57.59%; τ² = 0.0324.

Figure 8. Forest plot of the CLIF-C ACLF score for 6-month mortality.


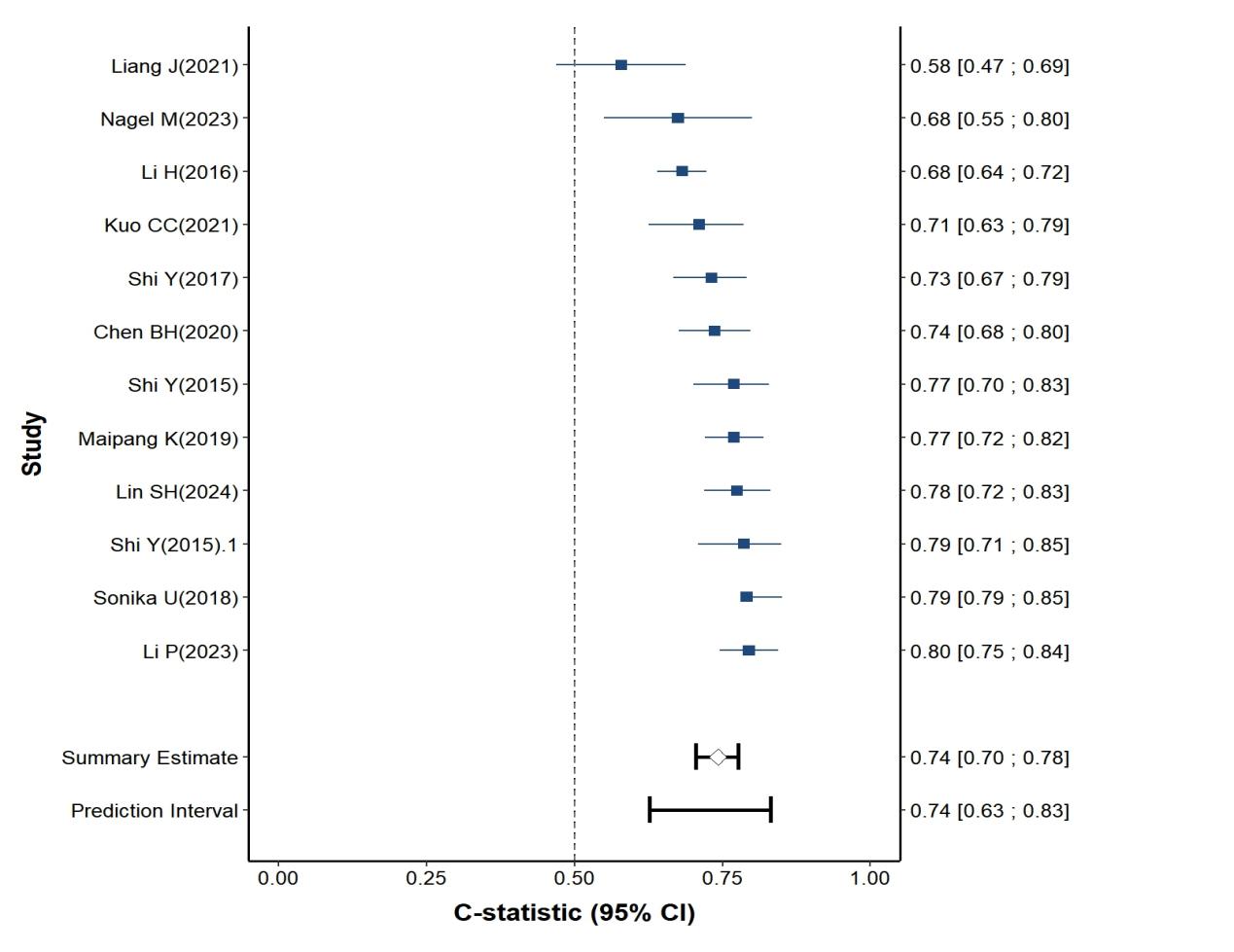


Note: Q =35.45 (df =11, p =0.0002); I²=67.21%; τ² =0.0512.

Figure 9. Forest plot of the CLIF-C ACLF score for 1-year mortality.


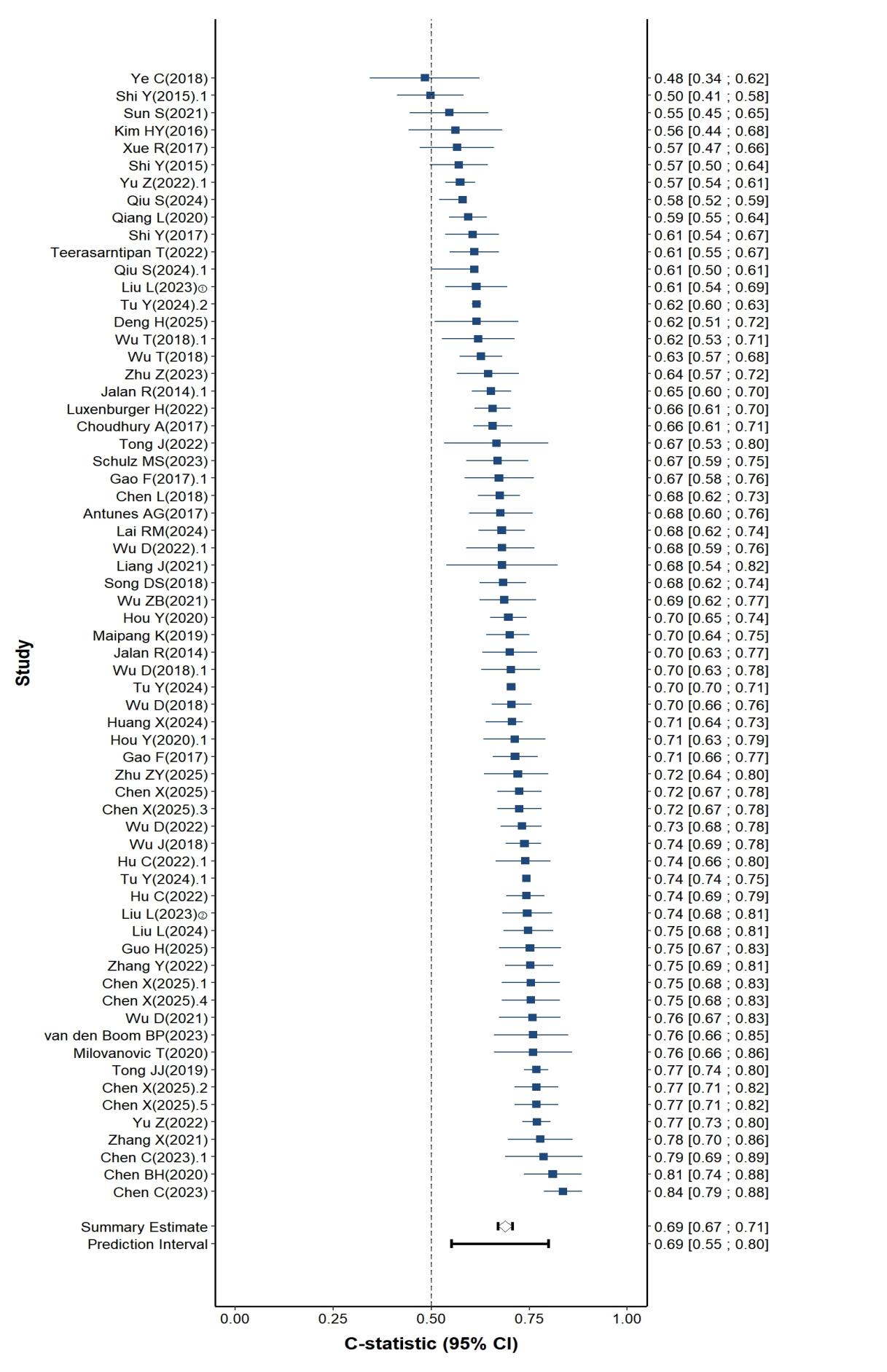


Note: Q =782.7 (df =64, p <0.0001); I²=94.96%; τ² =0.0844.

Figure 10. Forest plot of the CTP score for 1-month mortality.


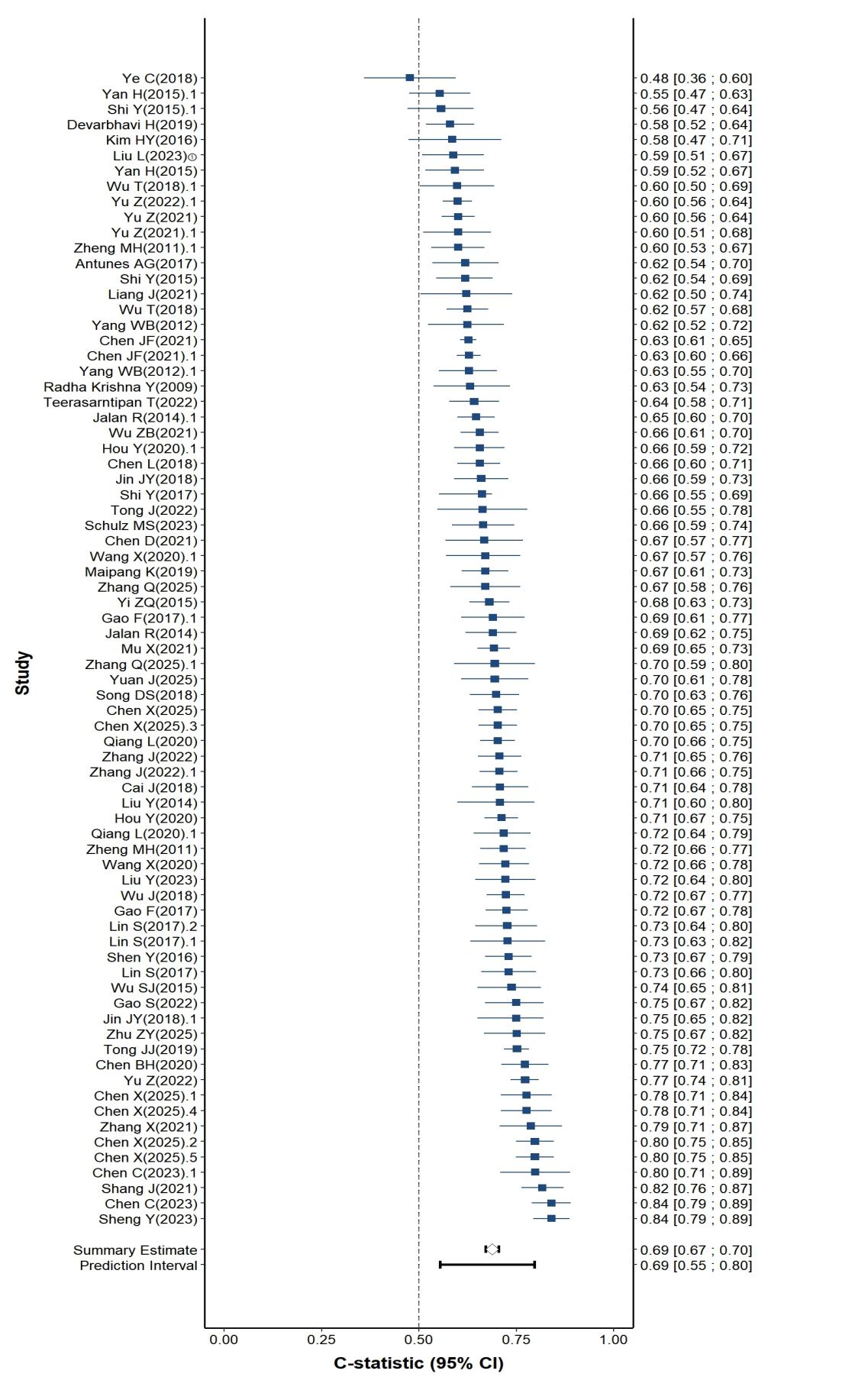


Note: Q =351.8 (df =74, p <0.0001); I²=80.53%; τ² =0.0820.

Figure 11. Forest plot of the CTP score for 3-month mortality.


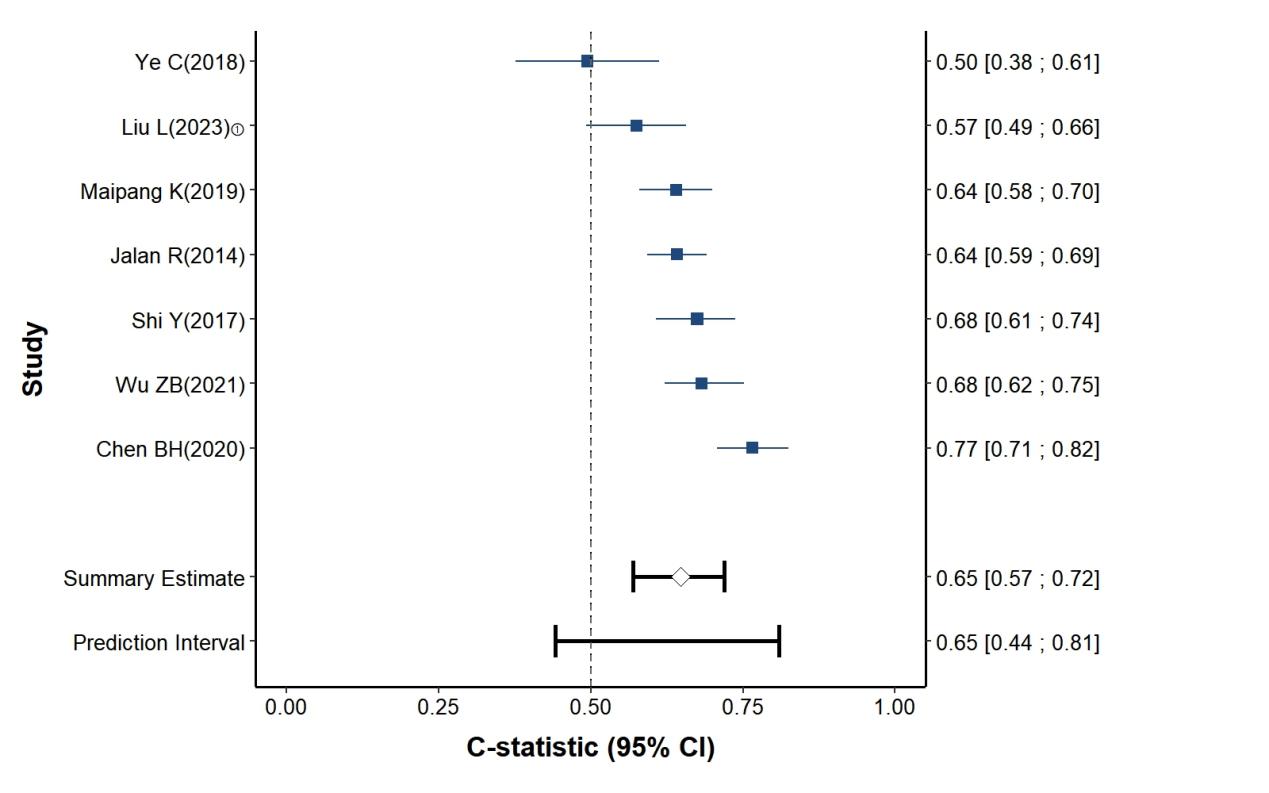


Note: Q =22.90 (df =6, p =0.0008); I²=79.02%; τ² = 0.0886.

Figure 12. Forest plot of the CTP score for 6-month mortality.


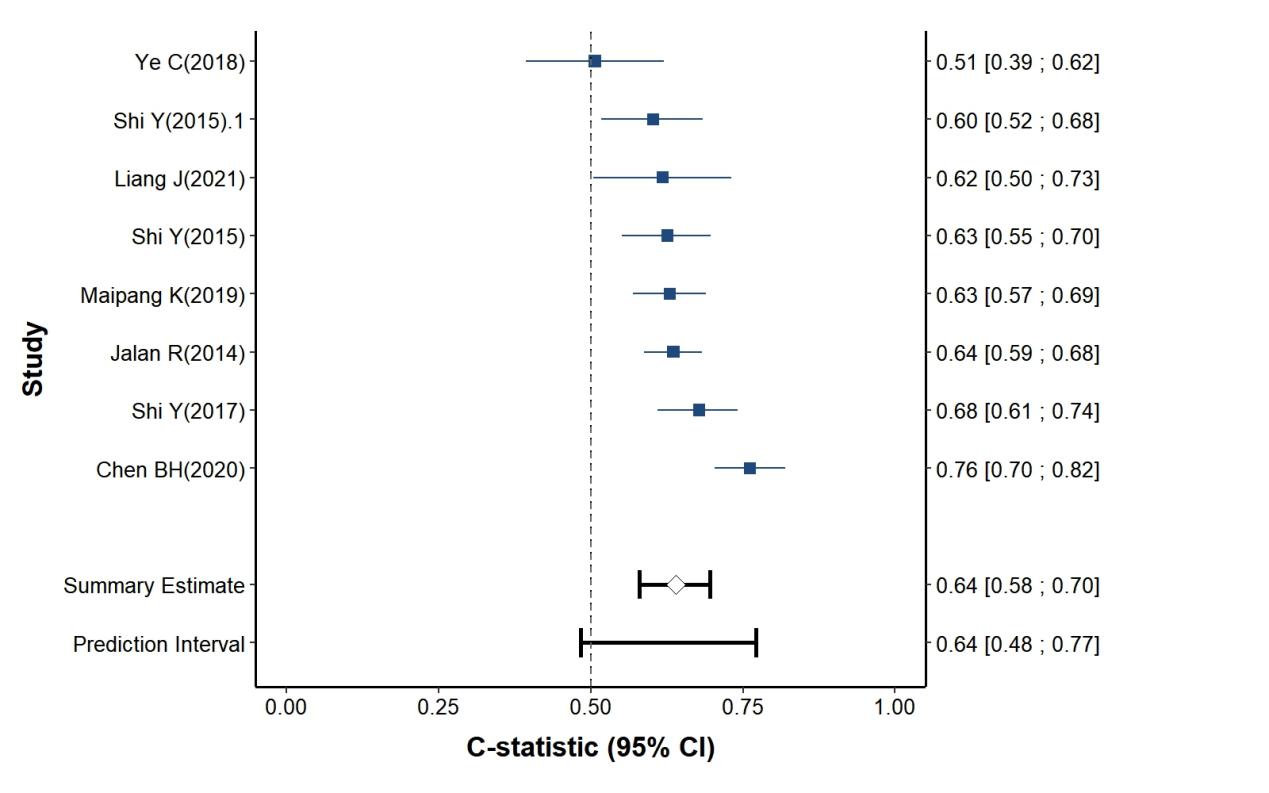


Note: Q =20.21 (df =7, p =0.0051); I²=69.31%; τ² = 0.0571.

Figure 13. Forest plot of the CTP score for 1-year mortality.


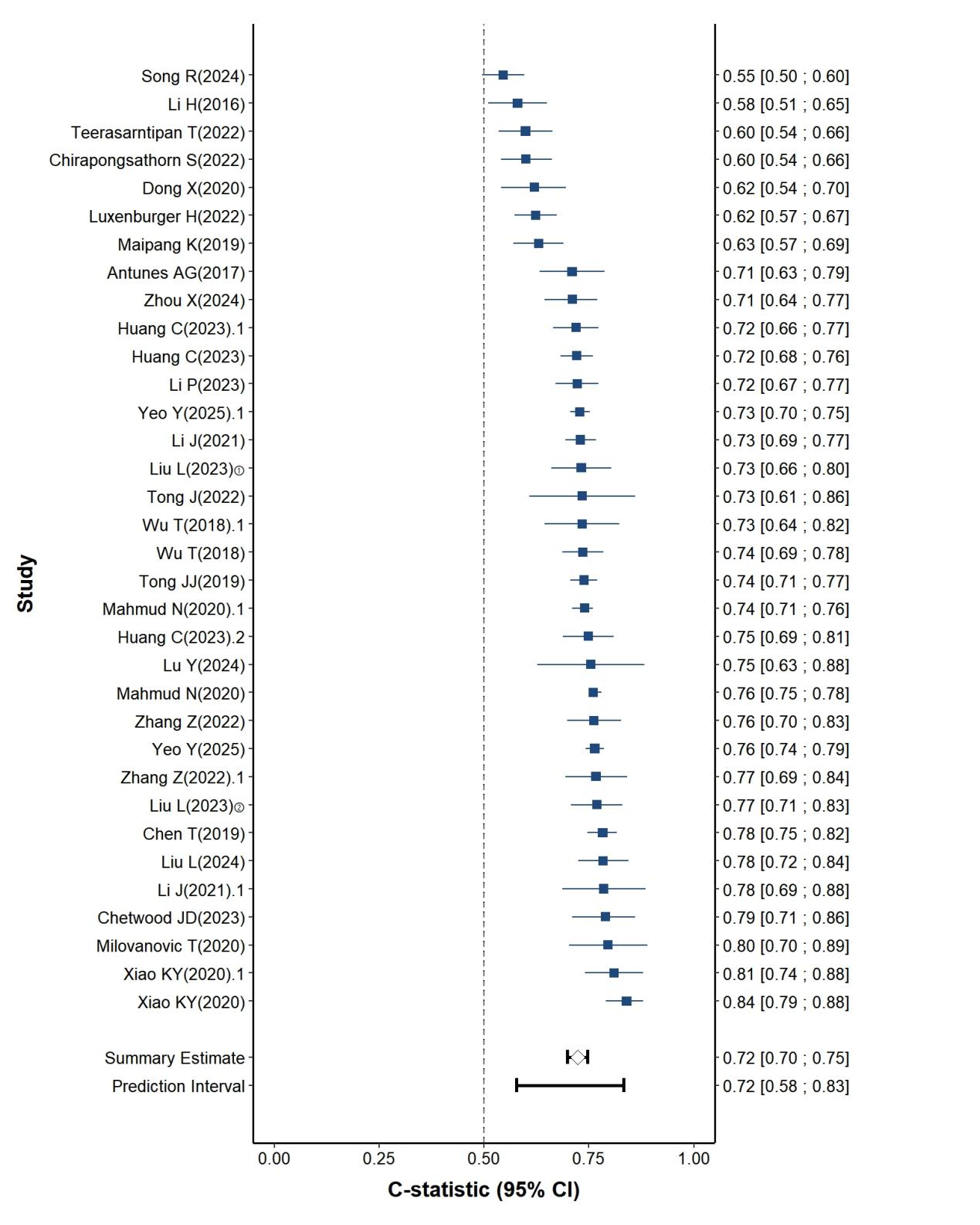


Note: Q = 223.37 (df =33, p <0.0001); I²=87.97%; τ² =0.0968.

Figure 14. Forest plot of the MELDNa score for 1-month mortality.


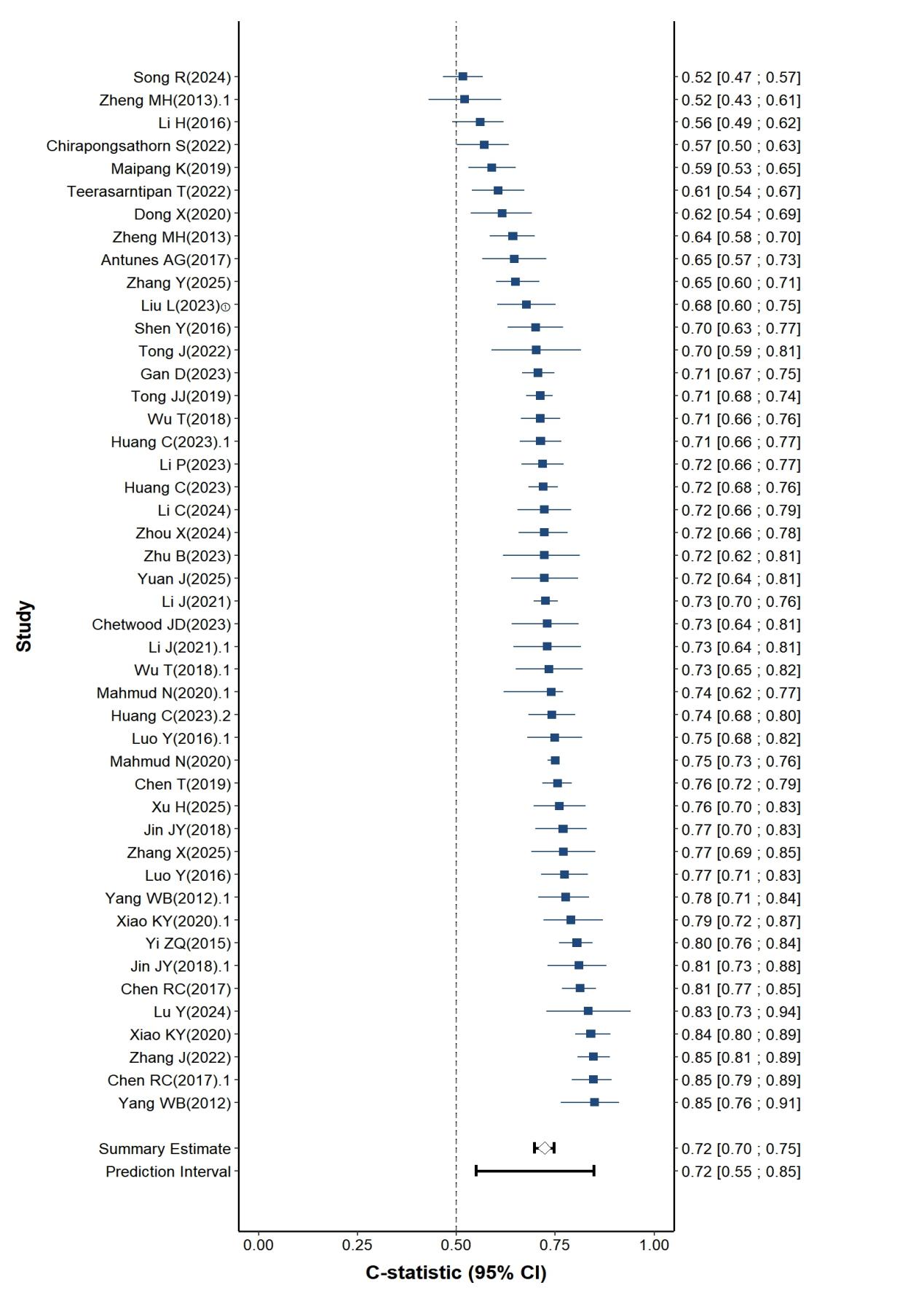


Note: Q =327.00 (df =45, p <0.0001); I² =88.27%; τ² =0.1384.

Figure 15. Forest plot of the MELDNa score for 3-month mortality.


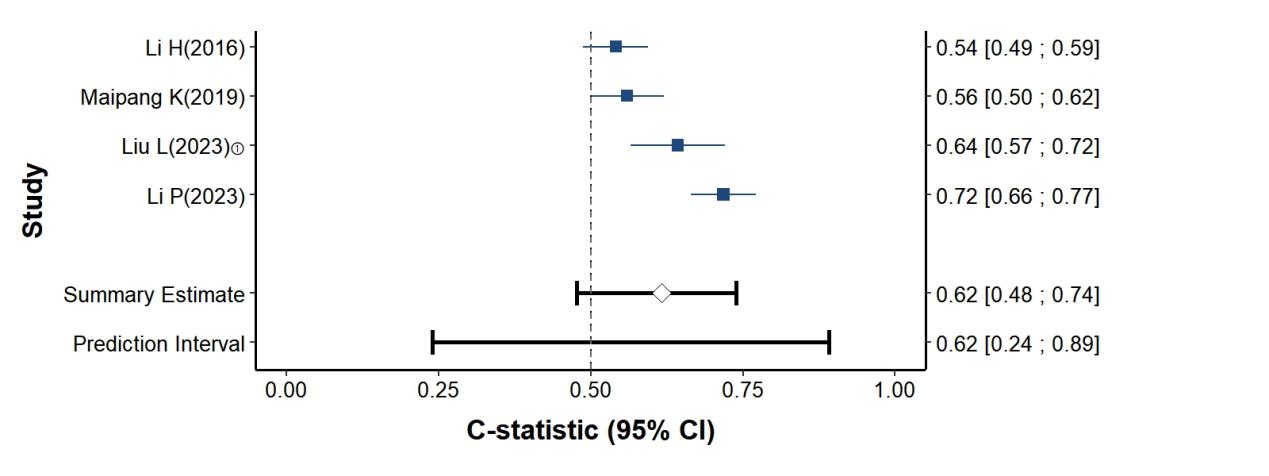


Note: Q =23.0 (df =3, p <0.0001); I²=86.34%; τ² =0.1109.

Figure 16. Forest plot of the MELDNa score for 6-month mortality.


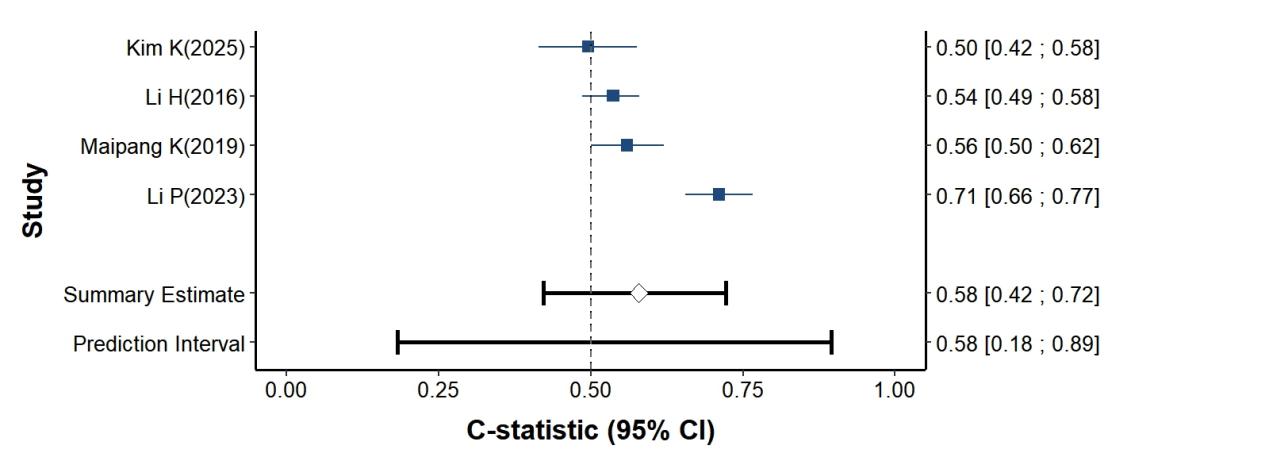


Note: Q =25.28 (df =3, p <0.0001); I²=89.54%; τ² =0.1391.

Figure 17. Forest plot of the MELDNa score for 1-year mortality.


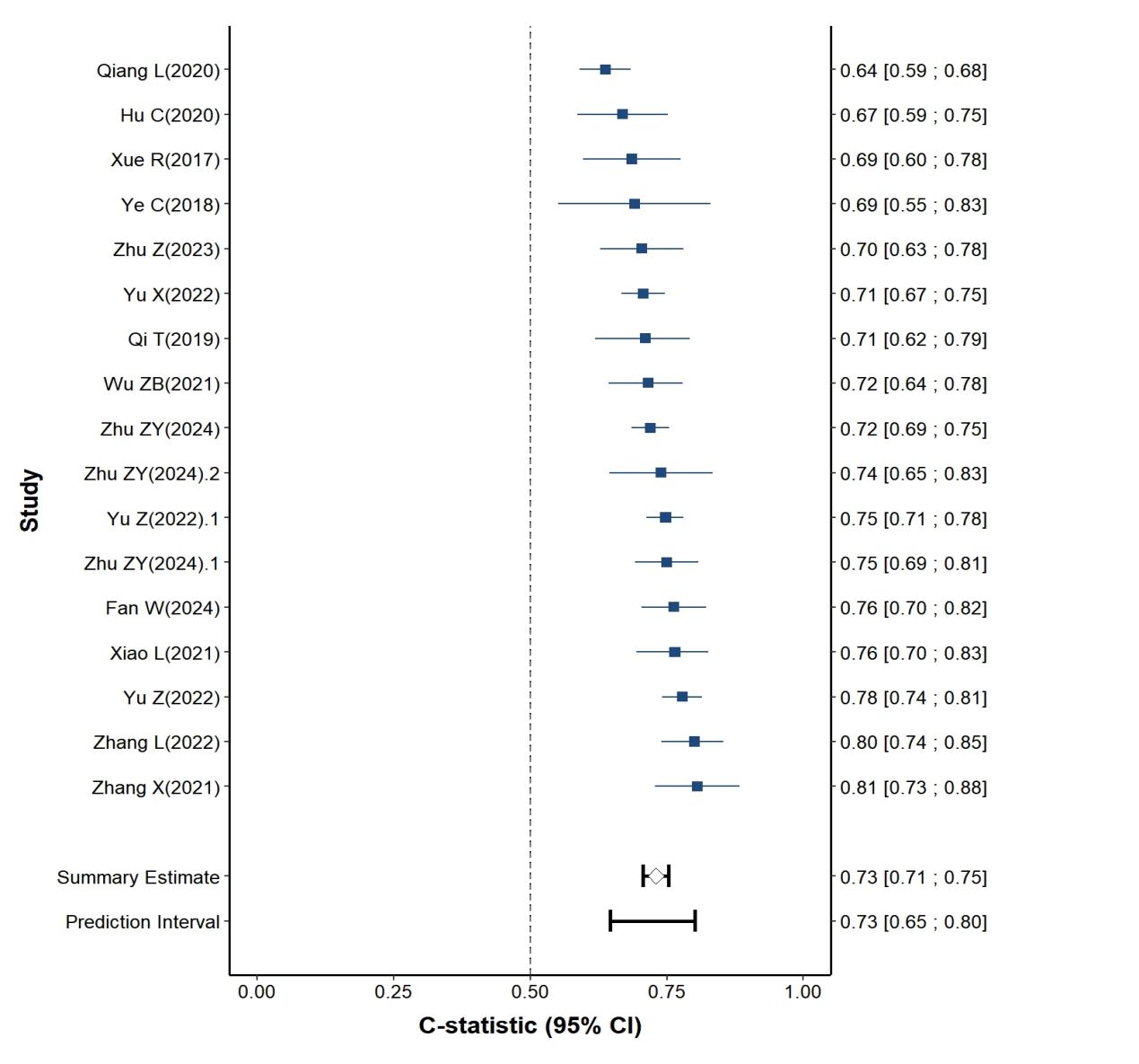


Note: Q =39.86 (df =16, p =0.0008); I²=89.54%; τ² =0.0312.

Figure 18. Forest plot of the MELD-Na score for 1-month mortality.


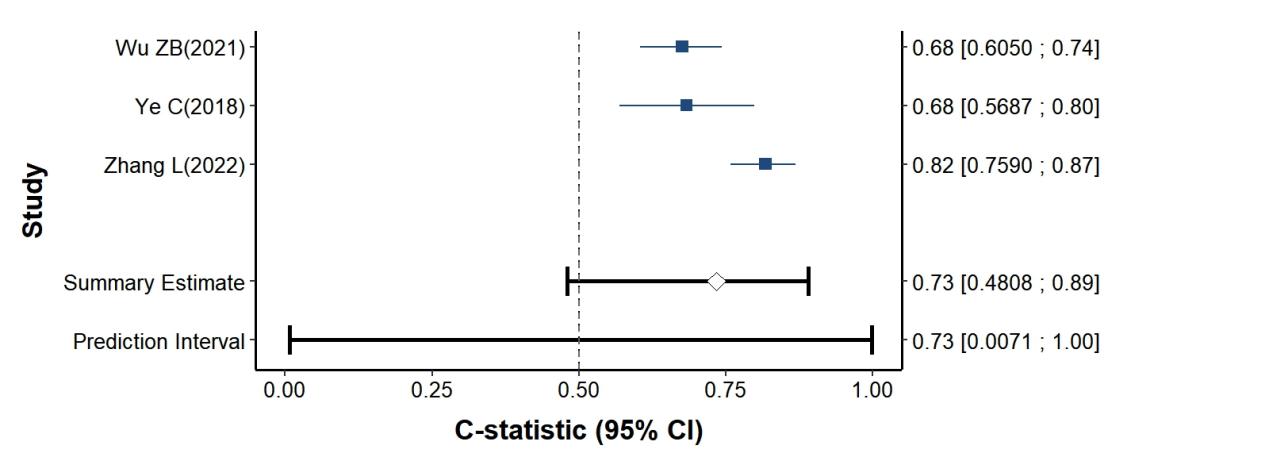


Note:Q =10.31(df =2, p =0.0058); I²=78.86%;τ² =0.1553.

Figure 18. Forest plot of the MELD-Na score for 6-month mortality.


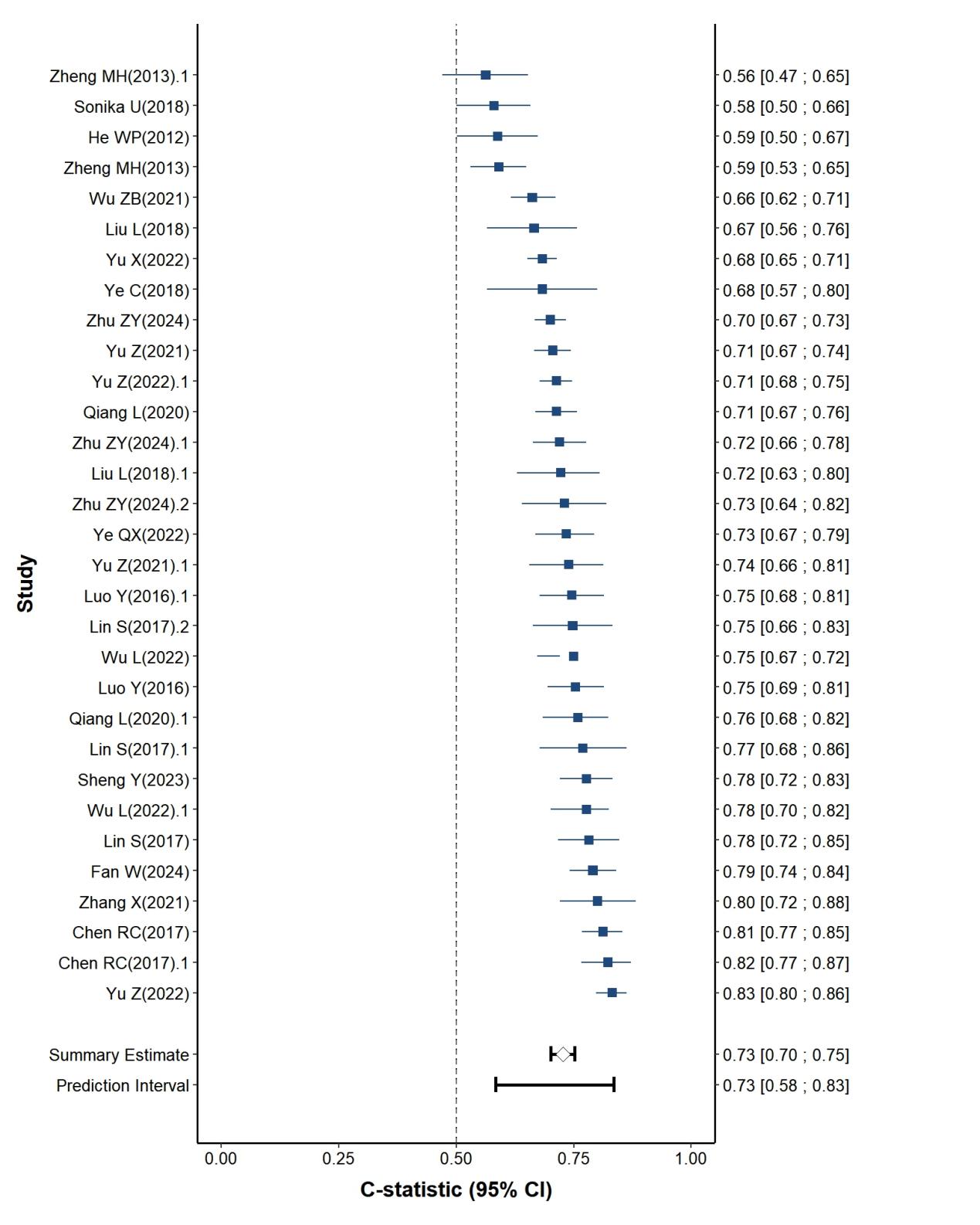


Note: Q =157.88 (df =30, p <0.0001); I²=84.03%; τ² =0.0943.

Figure 20. Forest plot of the MELD-Na score for 3-month mortality.


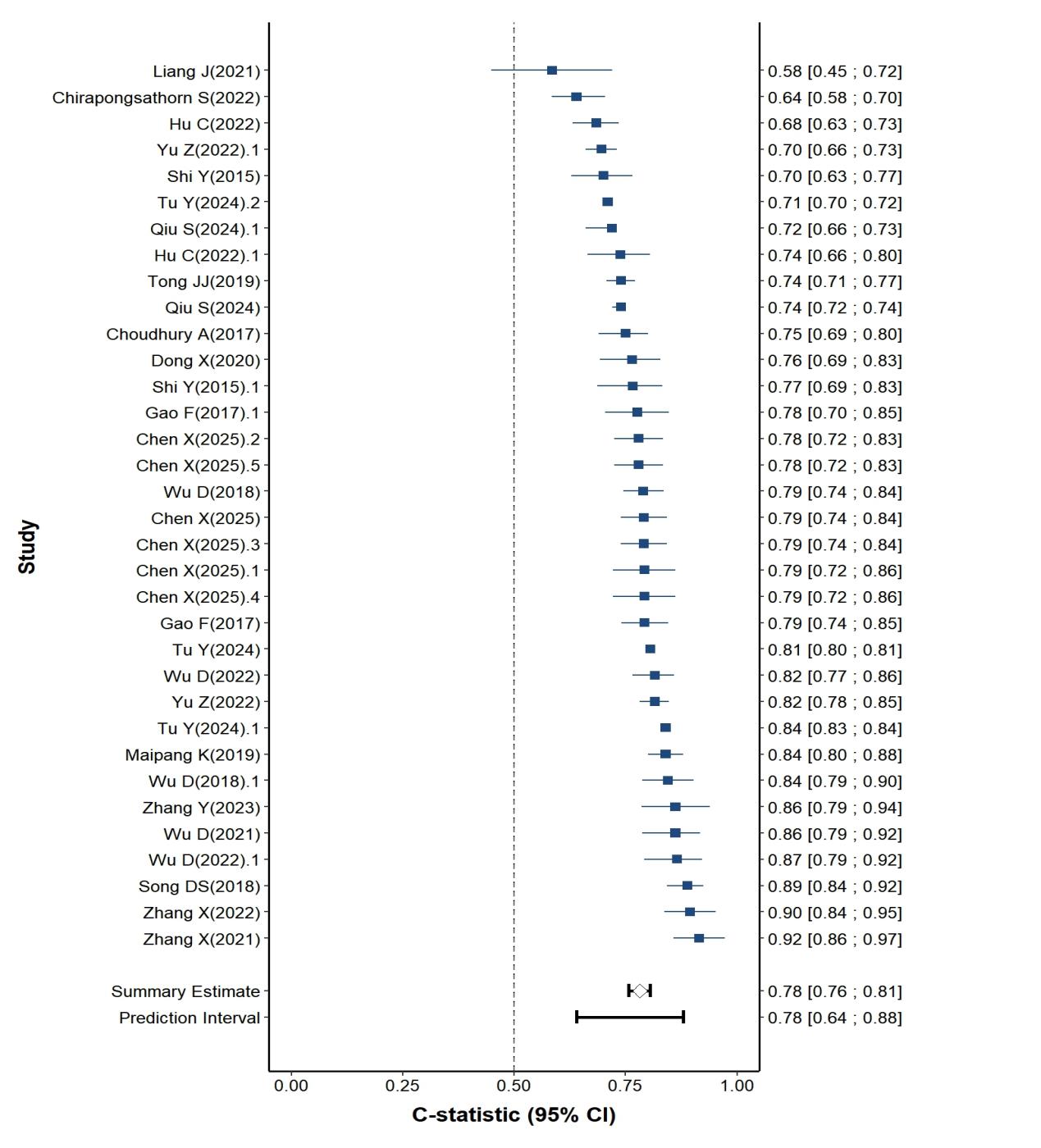


Note: Q =937.0 (df =33, p <0.0001); I²=97.45%; τ² =0.1144.

Figure 21. Forest plot of the CLIF-SOFA score for 1-month mortality.


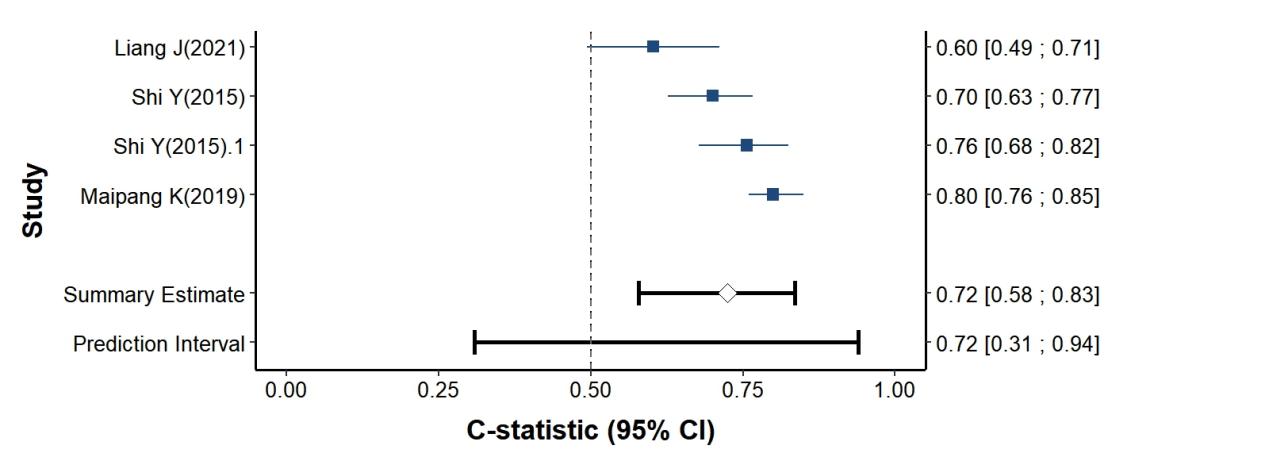


Note: Q =13.83 (df =3, p =0.0031); I²=78.86%; τ² =0.1284.

Figure 22. Forest plot of the CLIF-SOFA score for 1-year mortality.


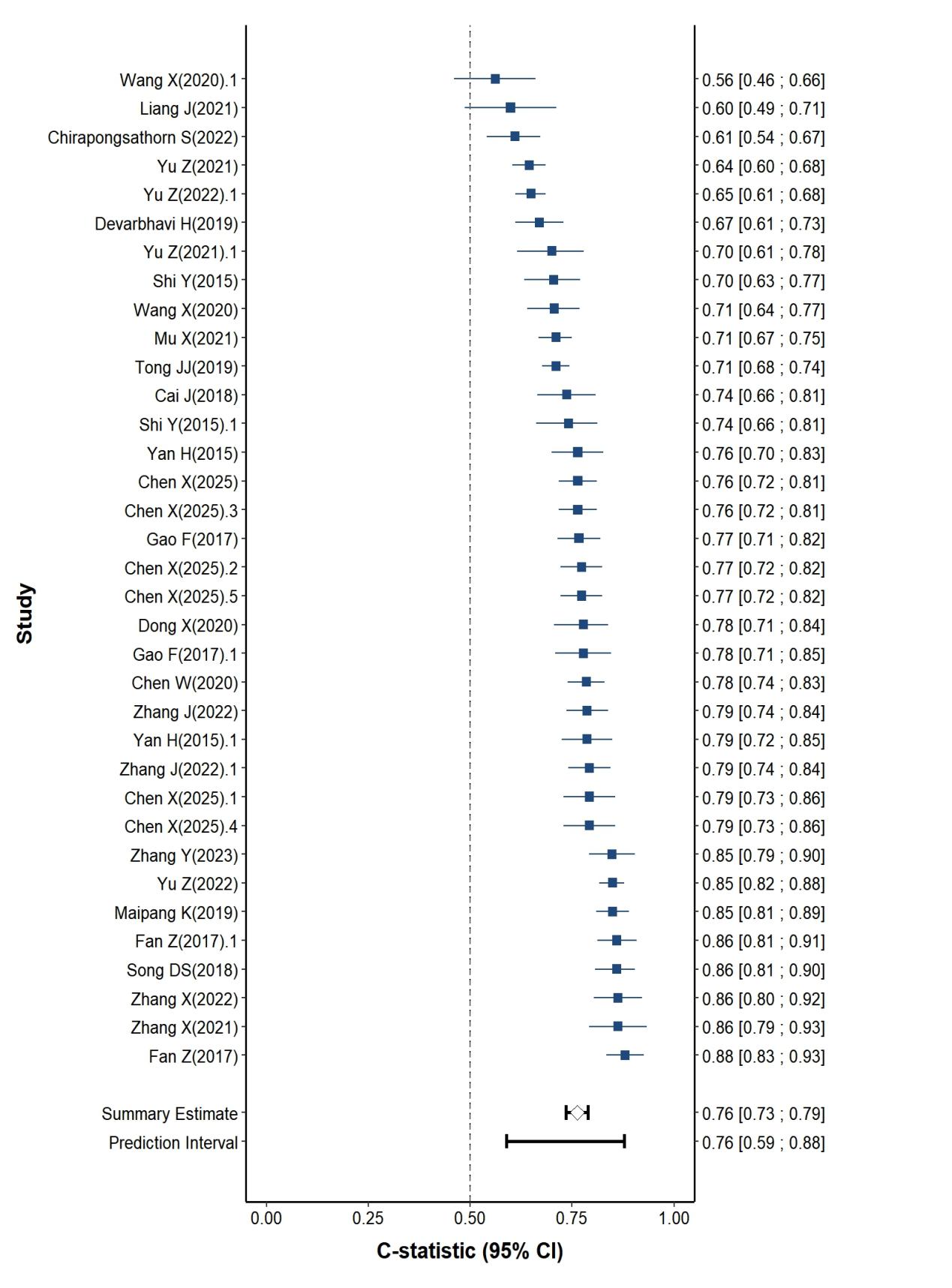


Note: Q =242.0 (df =34, p <0.0001); I²=86.84%; τ² =0.1526.

Figure 23. Forest plot of the CLIF-SOFA score for 3-month mortality.


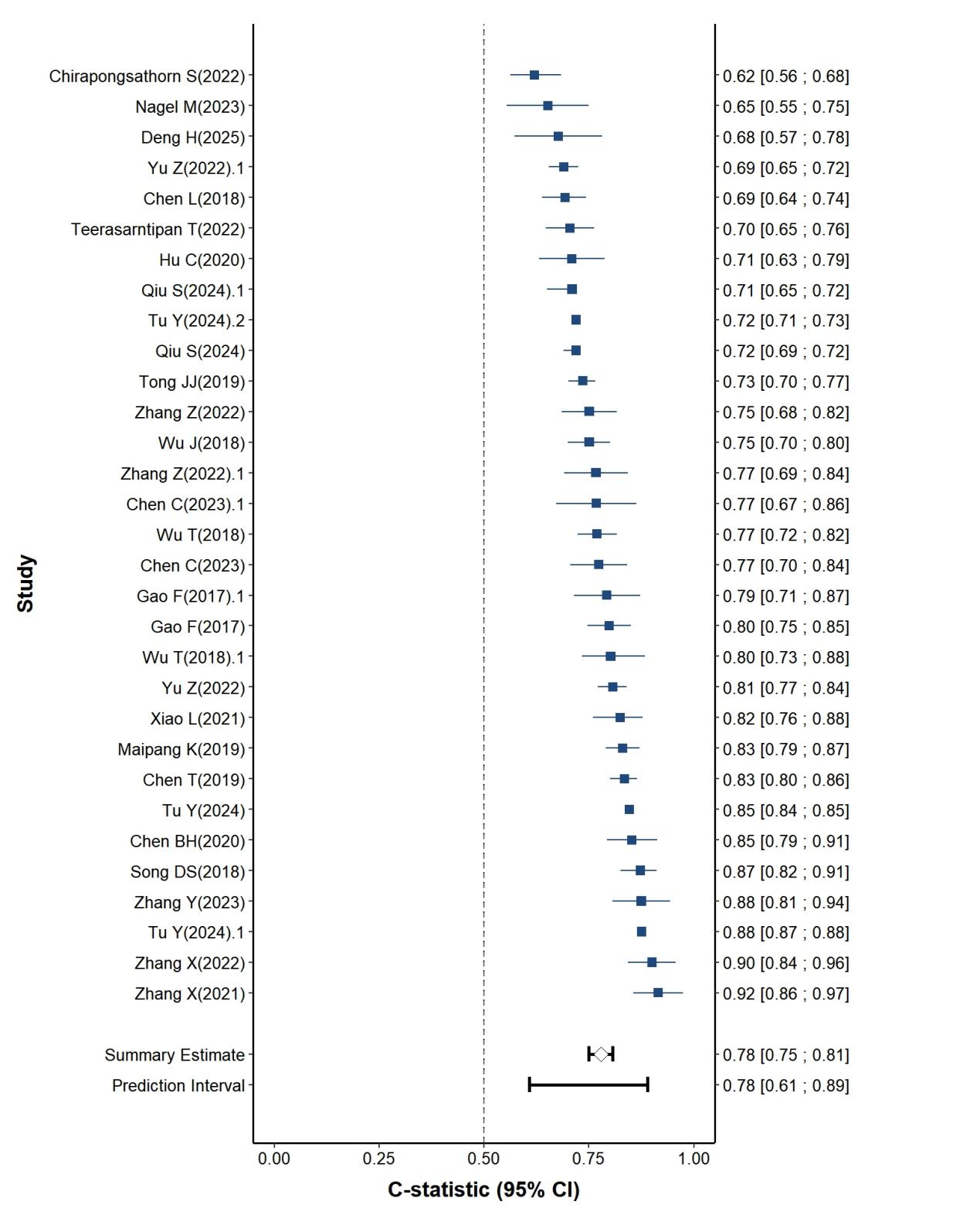


Note: Q =1701.2 (df =30, p <0.0001); I² =97.79%; τ² =0.1554.

Figure 24. Forest plot of the CLIF-C OFS score for 1-month mortality.


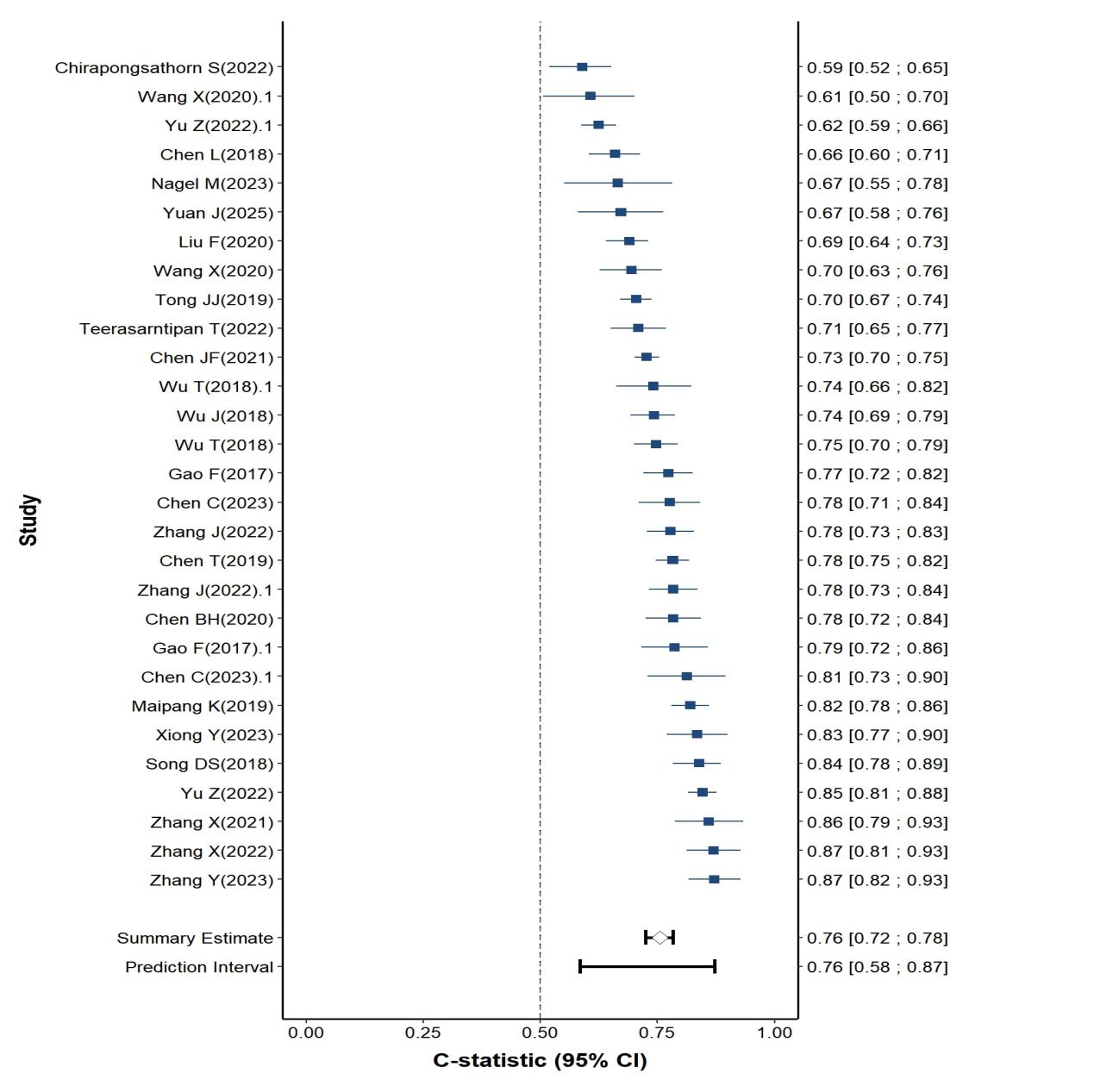


Note: Q =1701.2(df =30, p <0.0001); I² =97.79%; τ² =0.1554.

Figure 24. Forest plot of the CLIF-C OFS score for 1-month mortality.

Note: Q =198.04 (df =28, p <0.0001); I² =87.57%; τ² = 0.1411.

Figure 25. Forest plot of the CLIF-C OFS score for 3-month mortality.


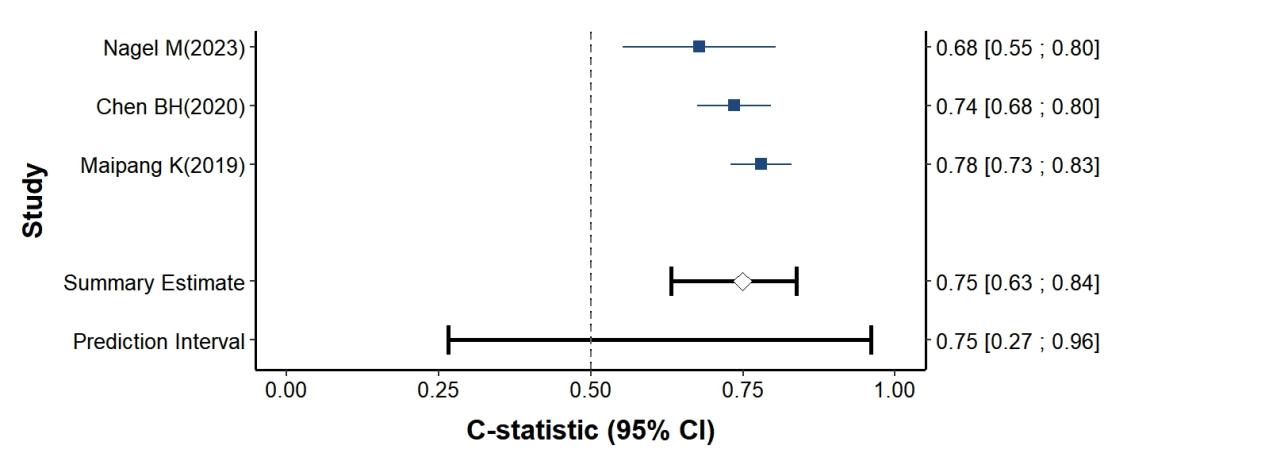


Note: Q =2.7 (df =2, p=0.2519); I²=23.44%; τ² =0.0111.

Figure 26. Forest plot of the CLIF-C OFS score for 1-year mortality.


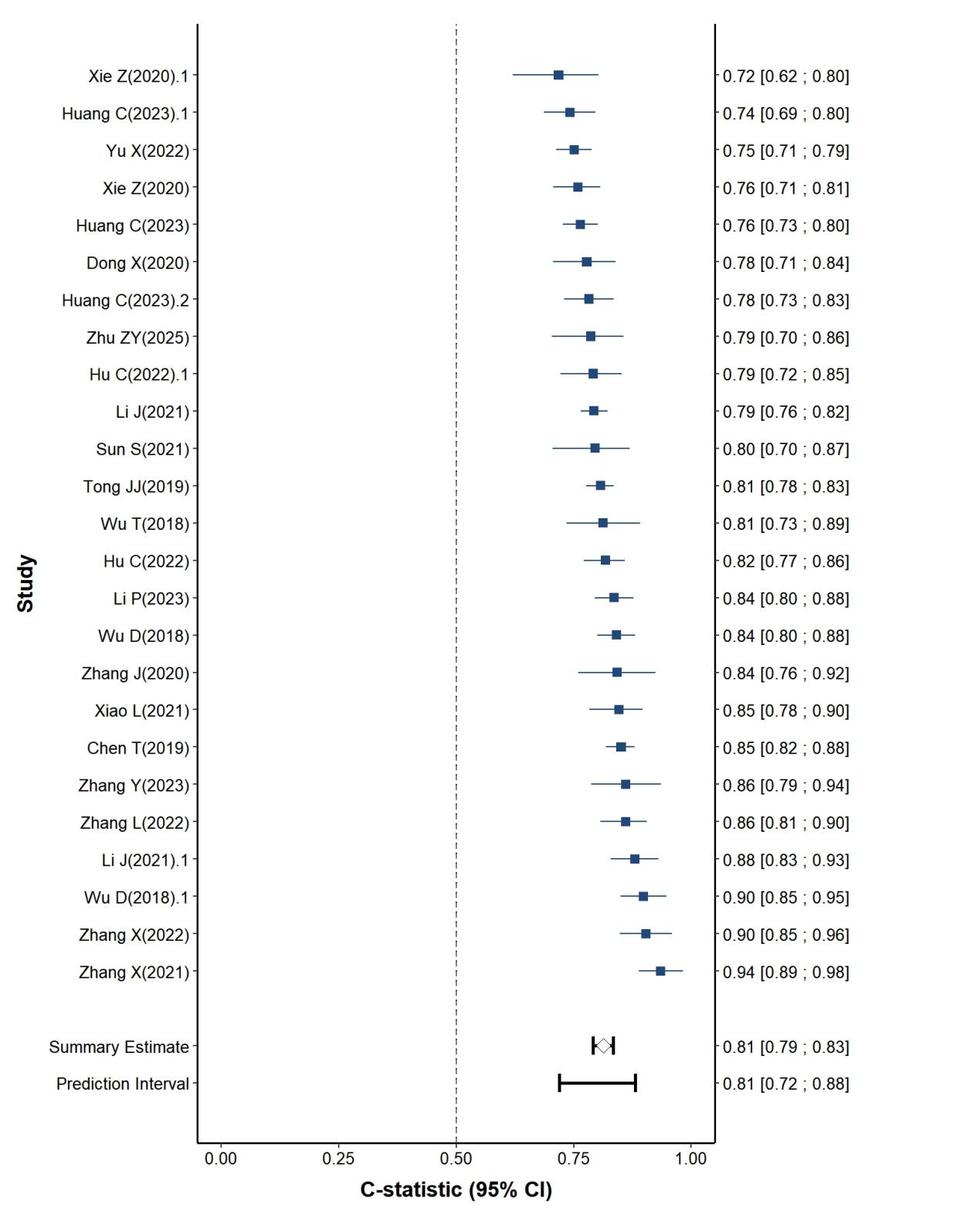


Note: Q =72.39 (df =24, p＜0.0001); I²=69.54%; τ² =0.0616.

Figure 27. Forest plot of the COSSH ACLF score for 1-month mortality.


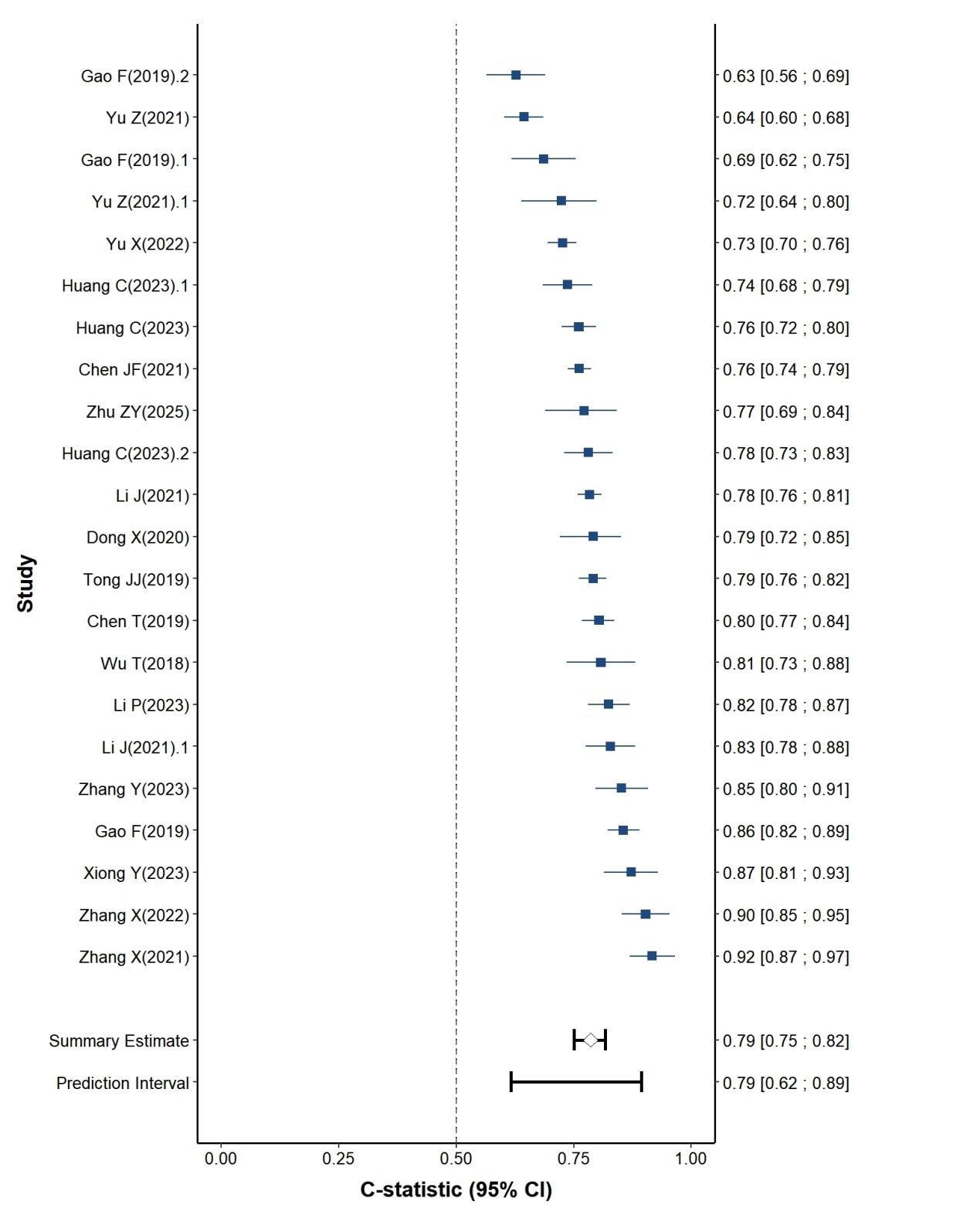


Note: Q =150.55 (df =21, p＜0.0001); I²=89.95%; τ² =0.1484.

Figure 28. Forest plot of the COSSH ACLF score for 3-month mortality.


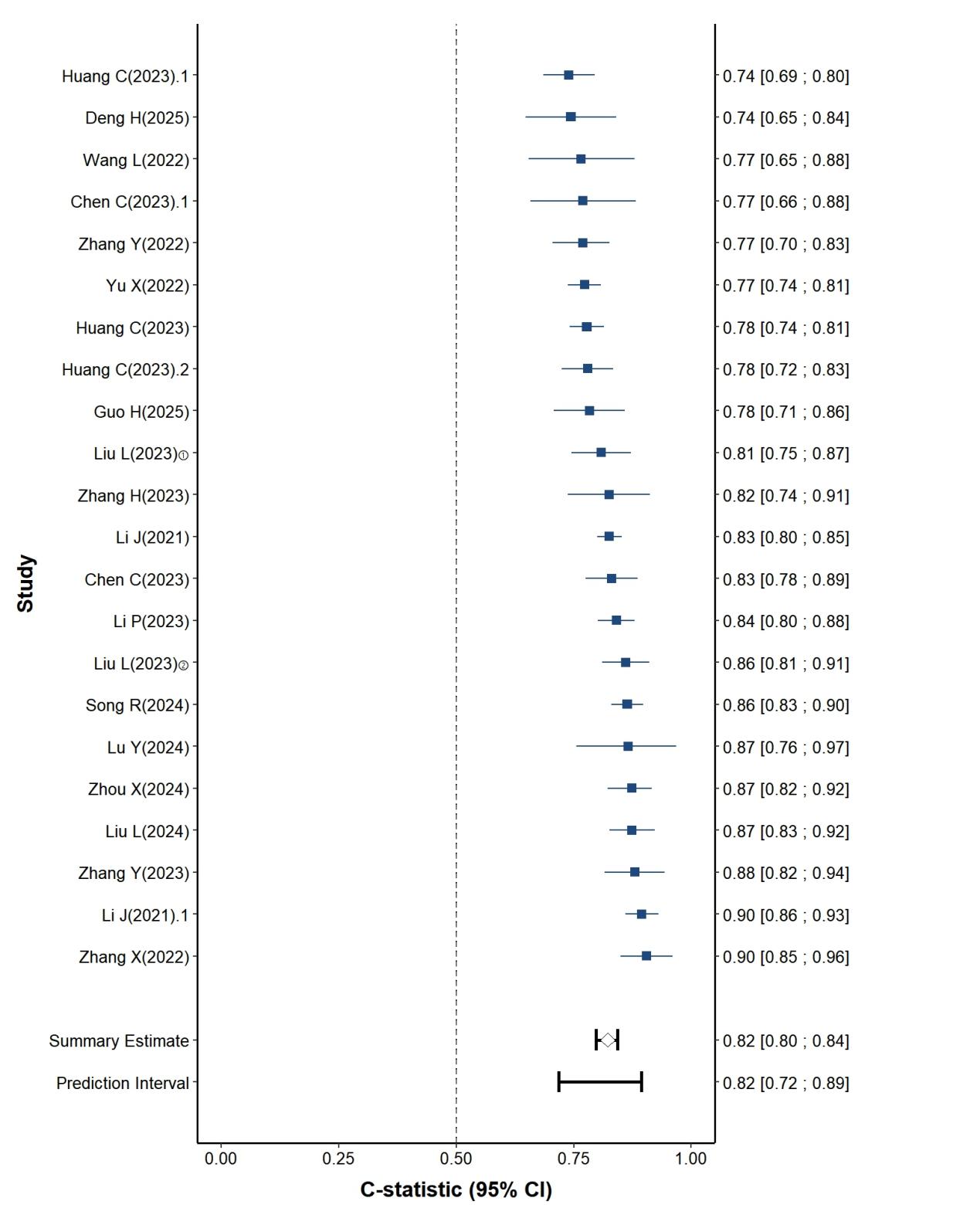


Note: Q =64.74 (df =21, p＜0.0001); I²=69.94%; τ² =0.0759.

Figure 29. Forest plot of the COSSH ACLF Ⅱ score for 1-month mortality.


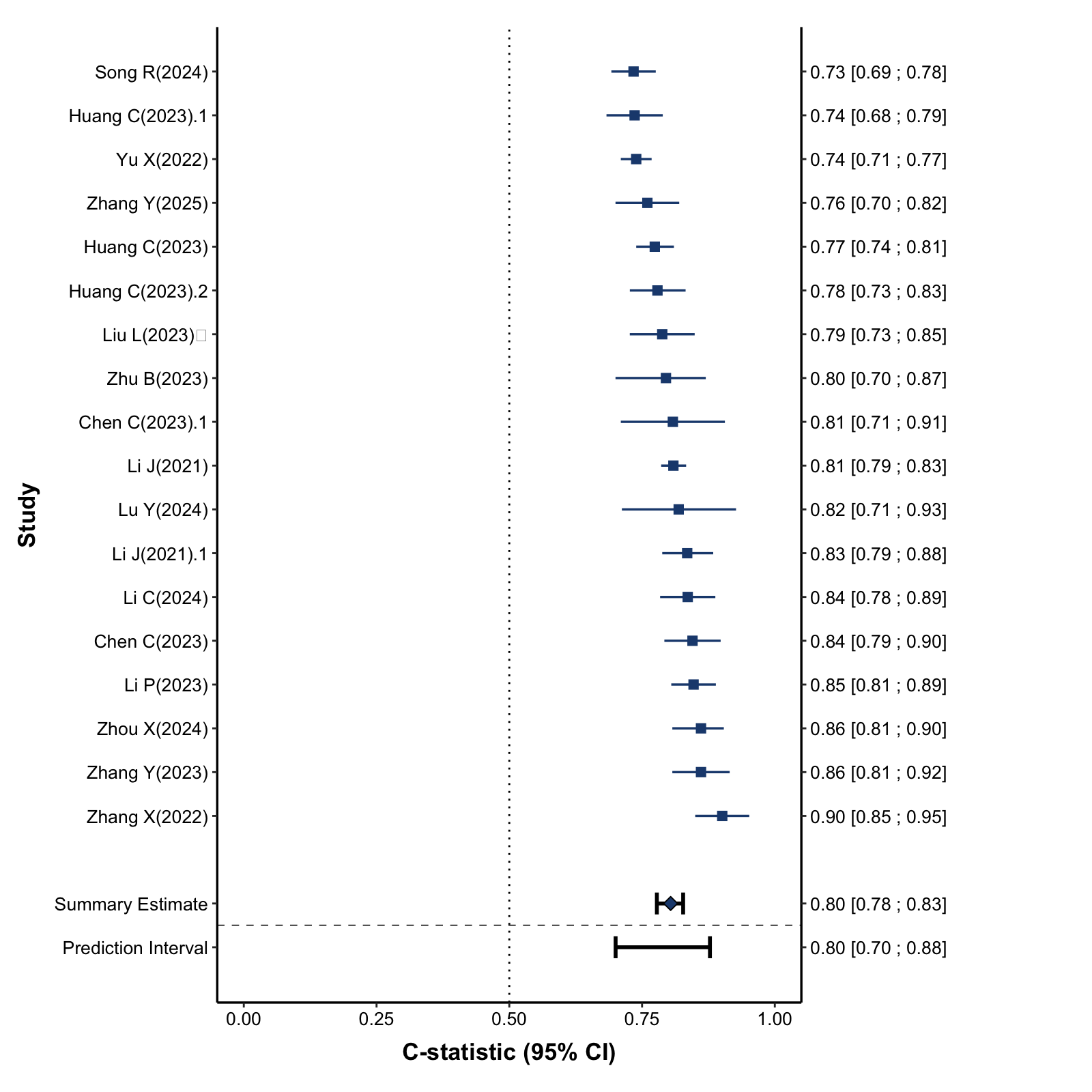


Note: Q =61.31 (df =17, p <0.0001); I²= 74.17%; τ² =0.0648.

Figure 30. Forest plot of the COSSH ACLF Ⅱ score for 3-month mortality.


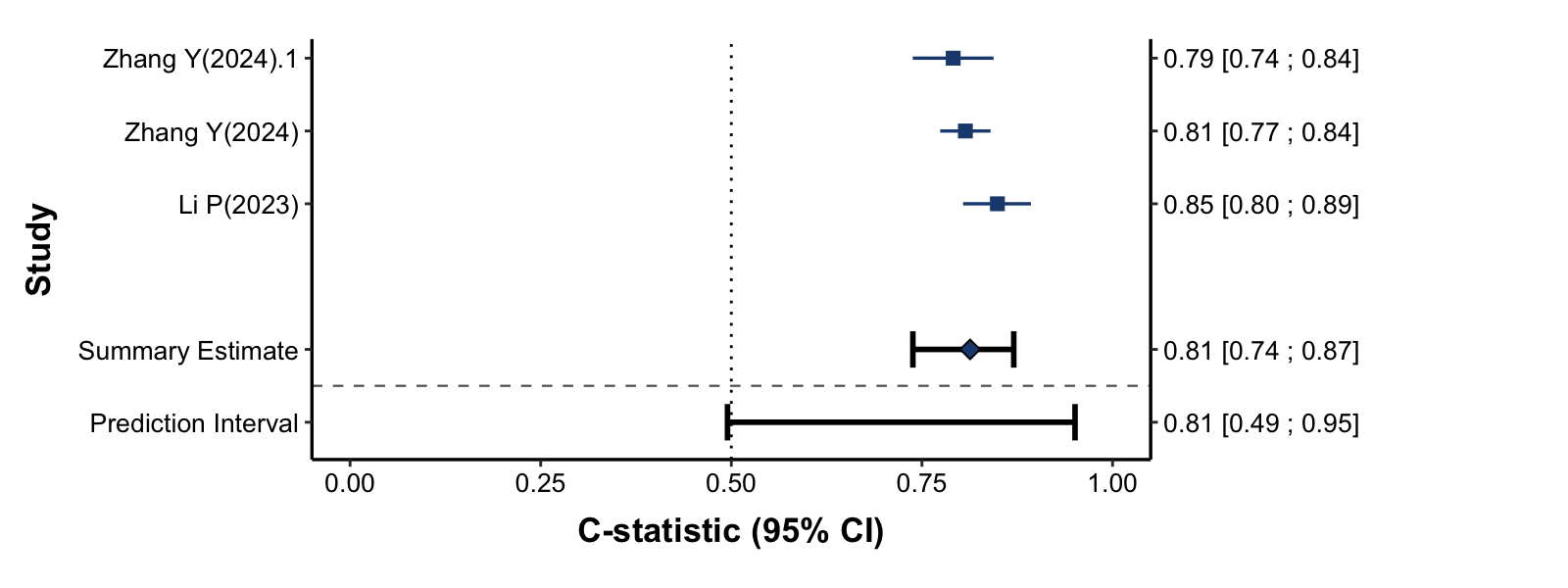


Note: Q =2.83 (df =2, p =0.2424); I²= 13.71%; τ² =0.0036.

Figure 31. Forest plot of the COSSH ACLF Ⅱ score for 1-year mortality.


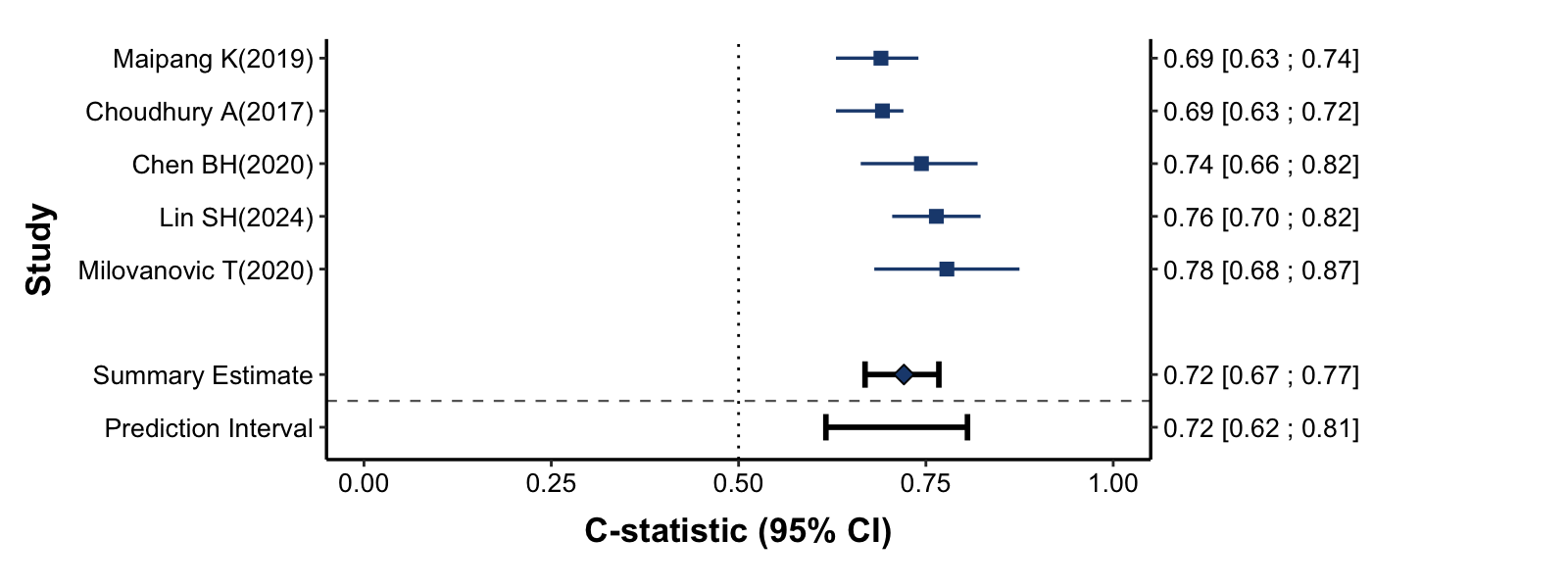


Note: Q =5.88 (df =4, p=0.2082); I²=35.59%; τ² =0.0143.

Figure 32. Forest plot of the APACHEⅡ score for 1-month mortality.


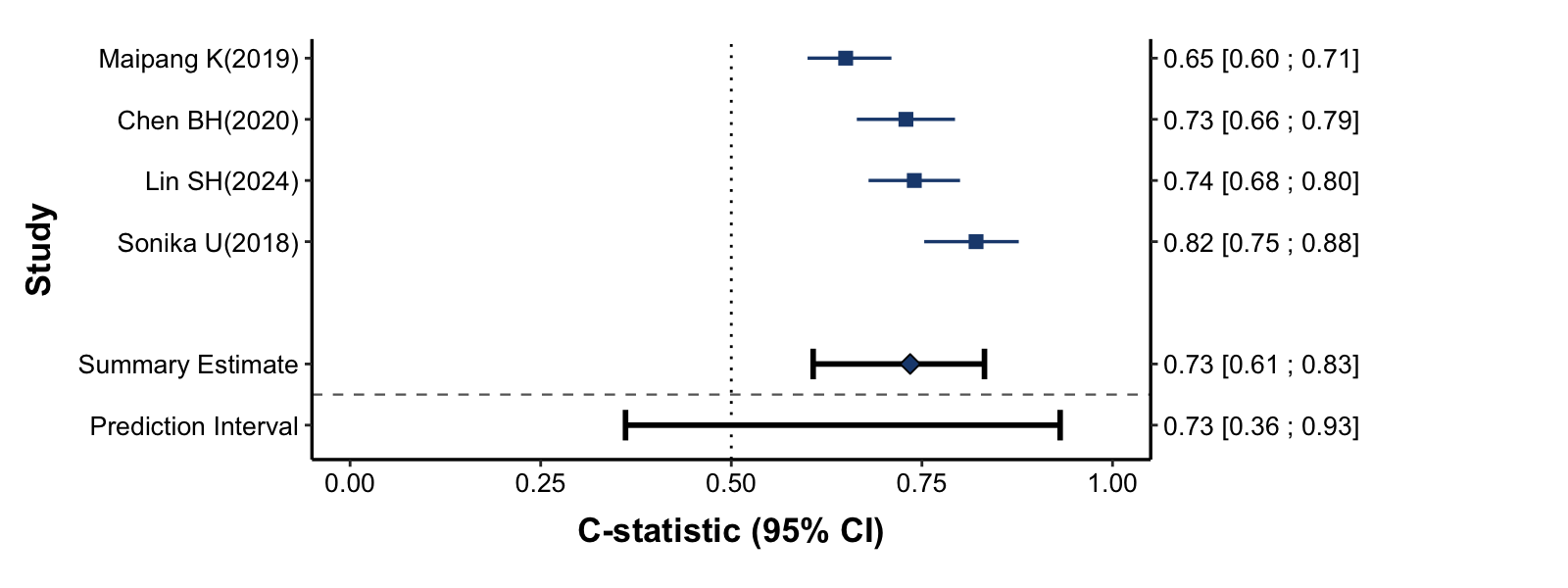


Note: Q =14.32 (df =3, p =0.0025); I²=79.49%; τ² =0.1028.

Figure 33. Forest plot of the APACHEⅡ score for 3-month mortality.


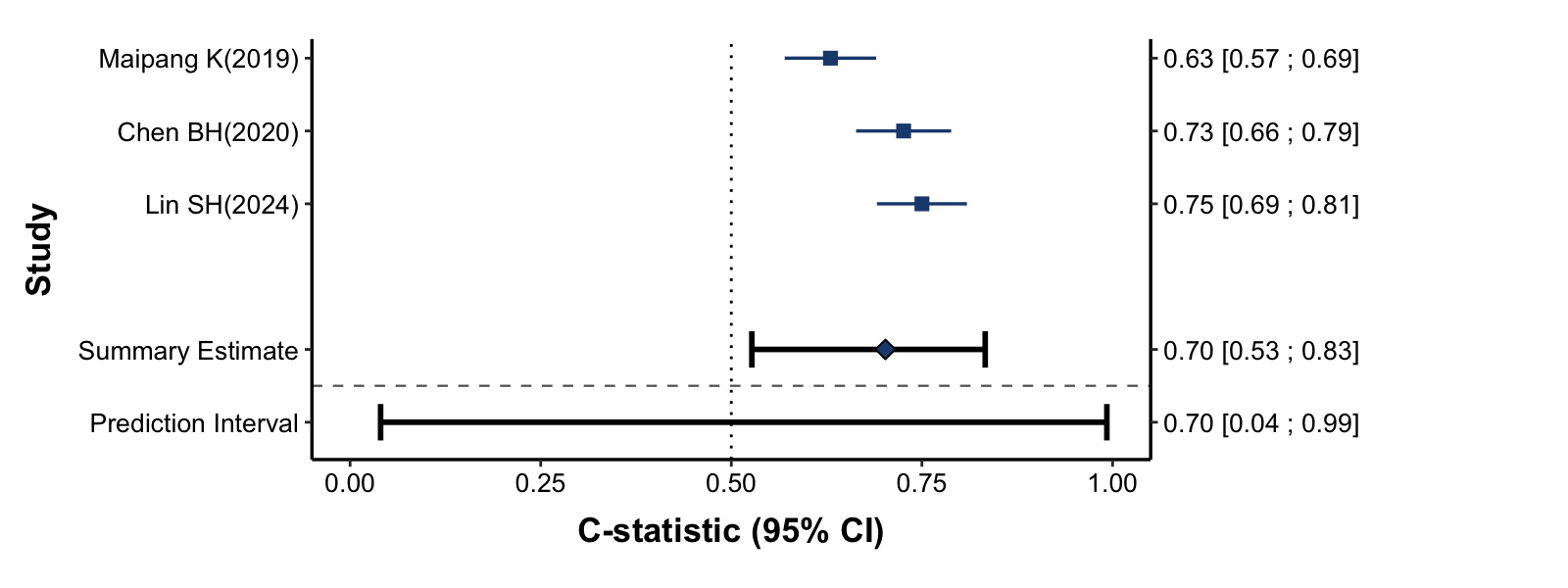


Note: Q =8.57 (df =2, p=0.0138); I²=75.35%; τ² =0.0704.

Figure 34. Forest plot of the APACHEⅡ score for 6-month mortality.


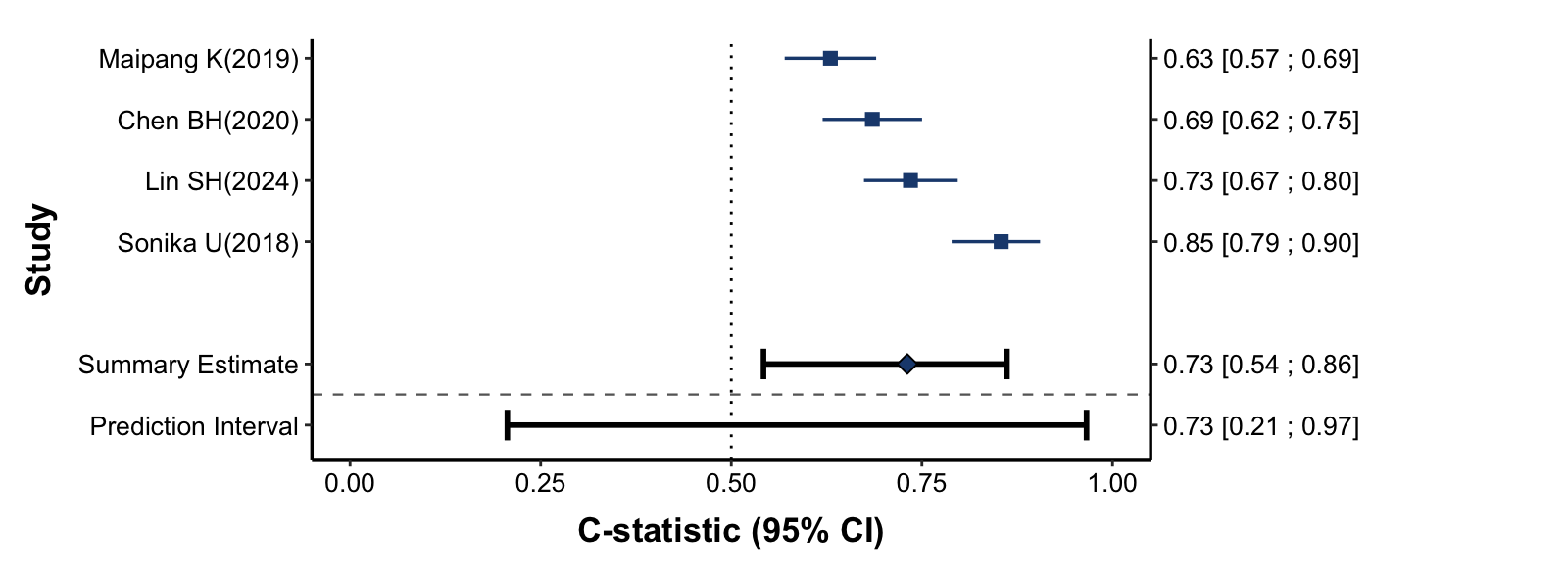


Note: Q =21.82 (df =3, p<0.0001); I²=89.36%; τ² =0.2294.

Figure 35. Forest plot of the APACHEⅡ score for 1-year mortality.


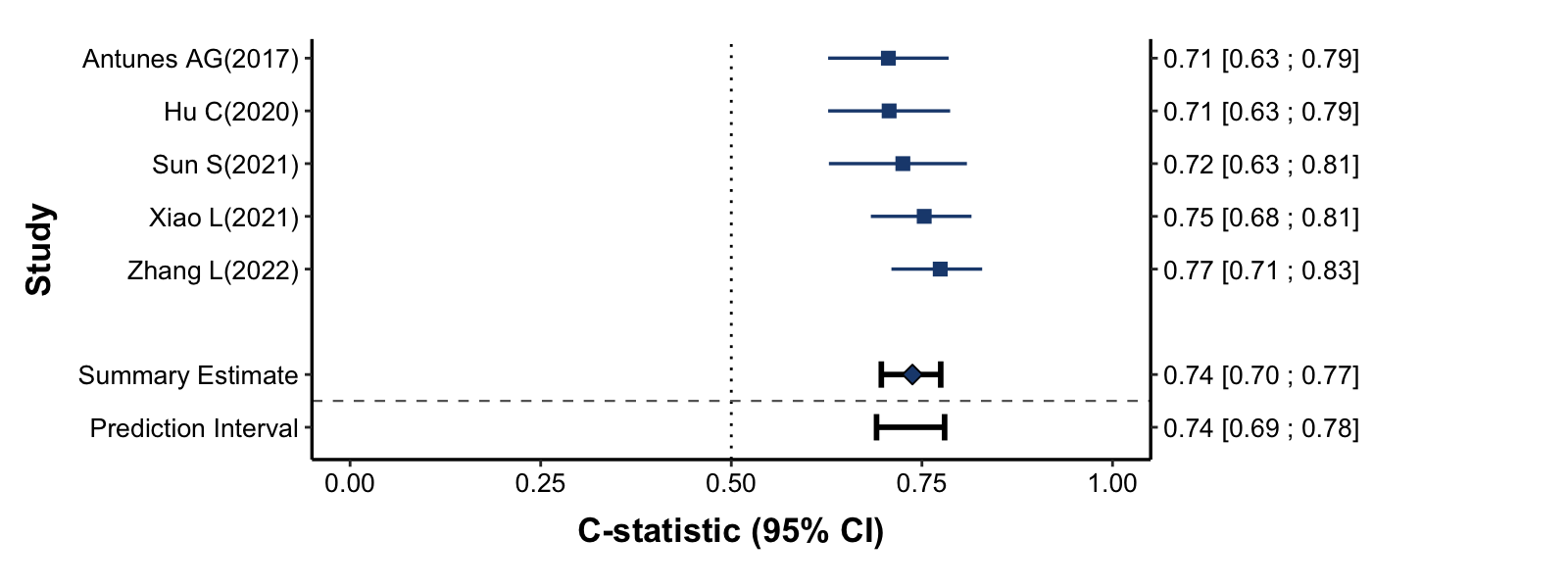


Note: Q =2.77(df =4, p=0.5974); I²=0%; τ² =0.

Figure 36. Forest plot of the iMELD score for 1-month mortality.


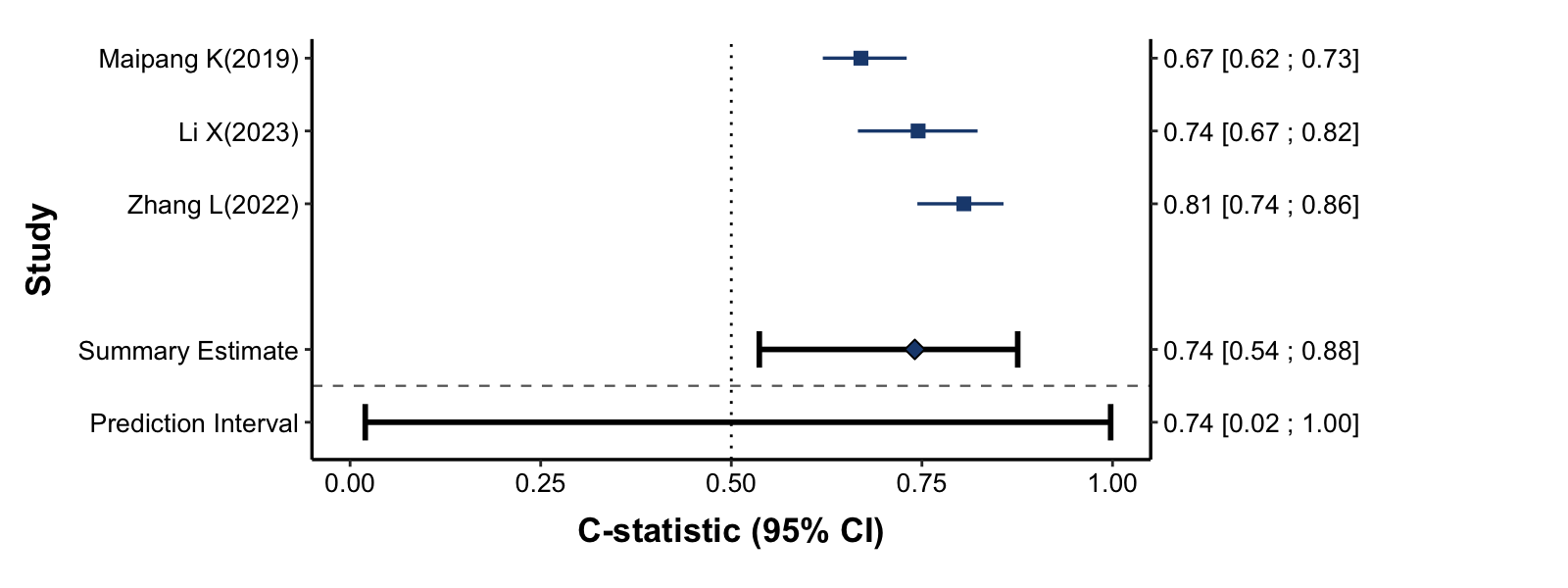


Note: Q =10.23 (df =2, p=0.0060); I²= 78.13%; τ² =0.1076.

Figure 37. Forest plot of the iMELD score for 6-month mortality.


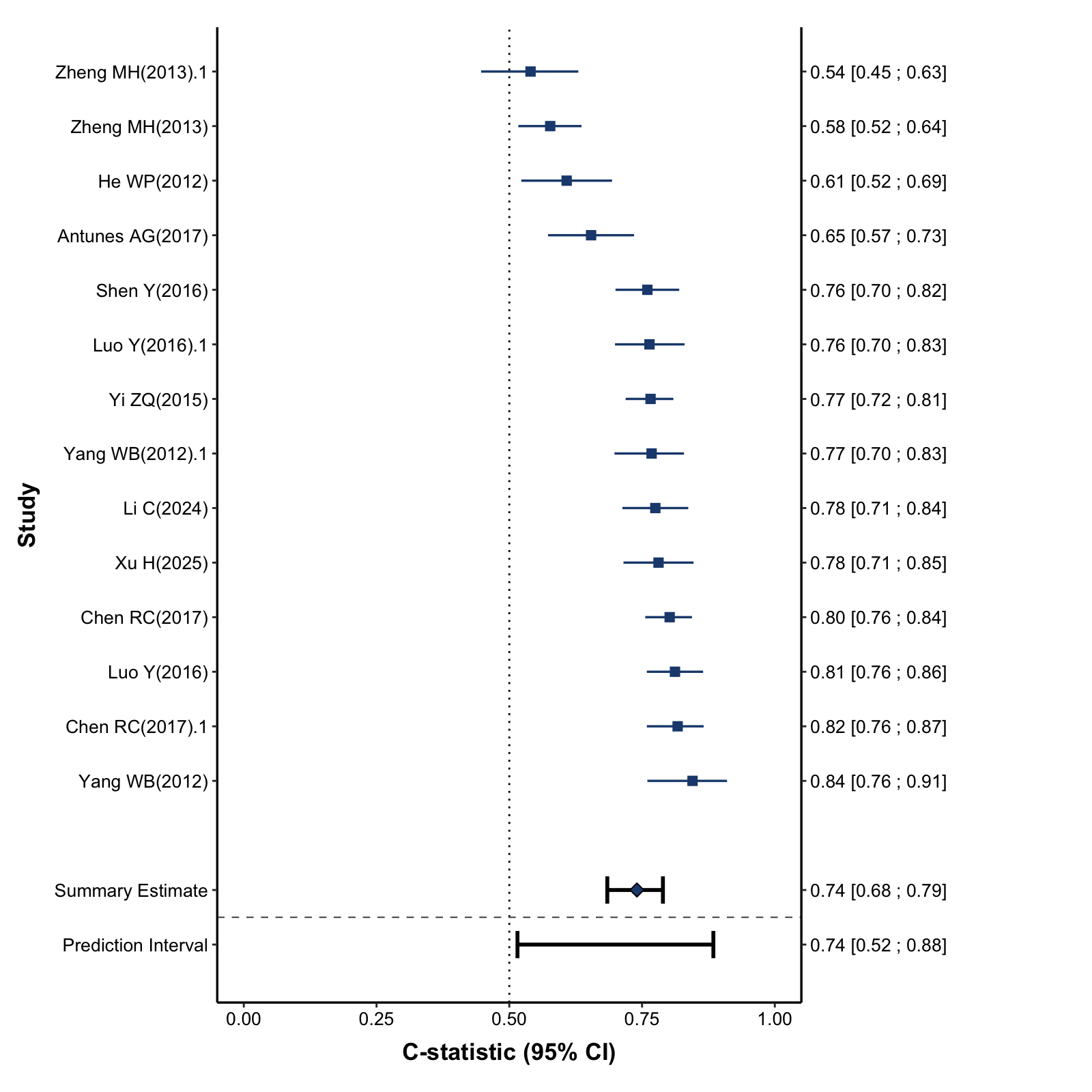


Note: Q =98.45 (df =13, p<0.0001); I²=86.36%; τ² =0.1889.

Figure 38. Forest plot of the iMELD score for 3-month mortality.


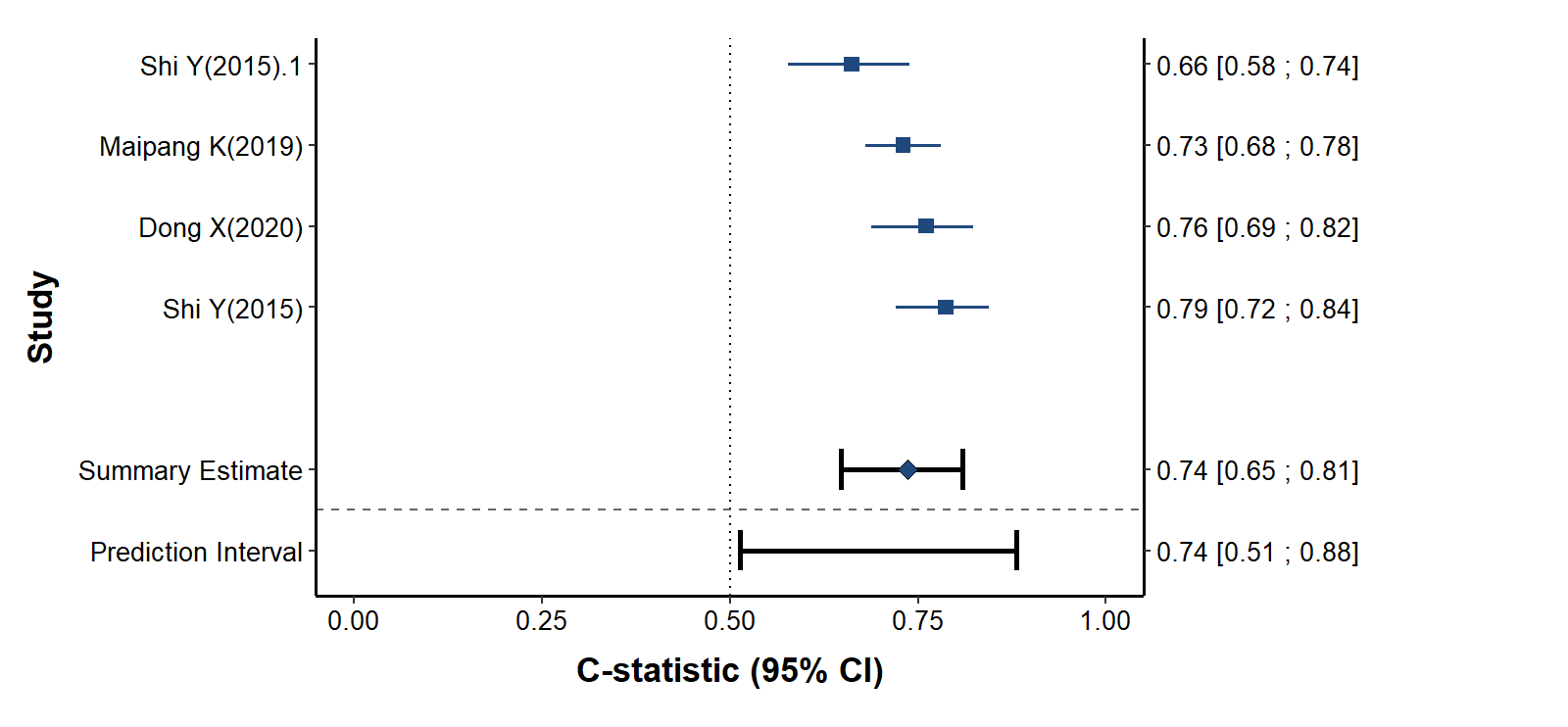


Note: Q =6.33 (df =3, p =0.097); I²=53.46%; τ²=0.0339.

Figure 39. Forest plot of the i-MELD score for 1-month mortality.


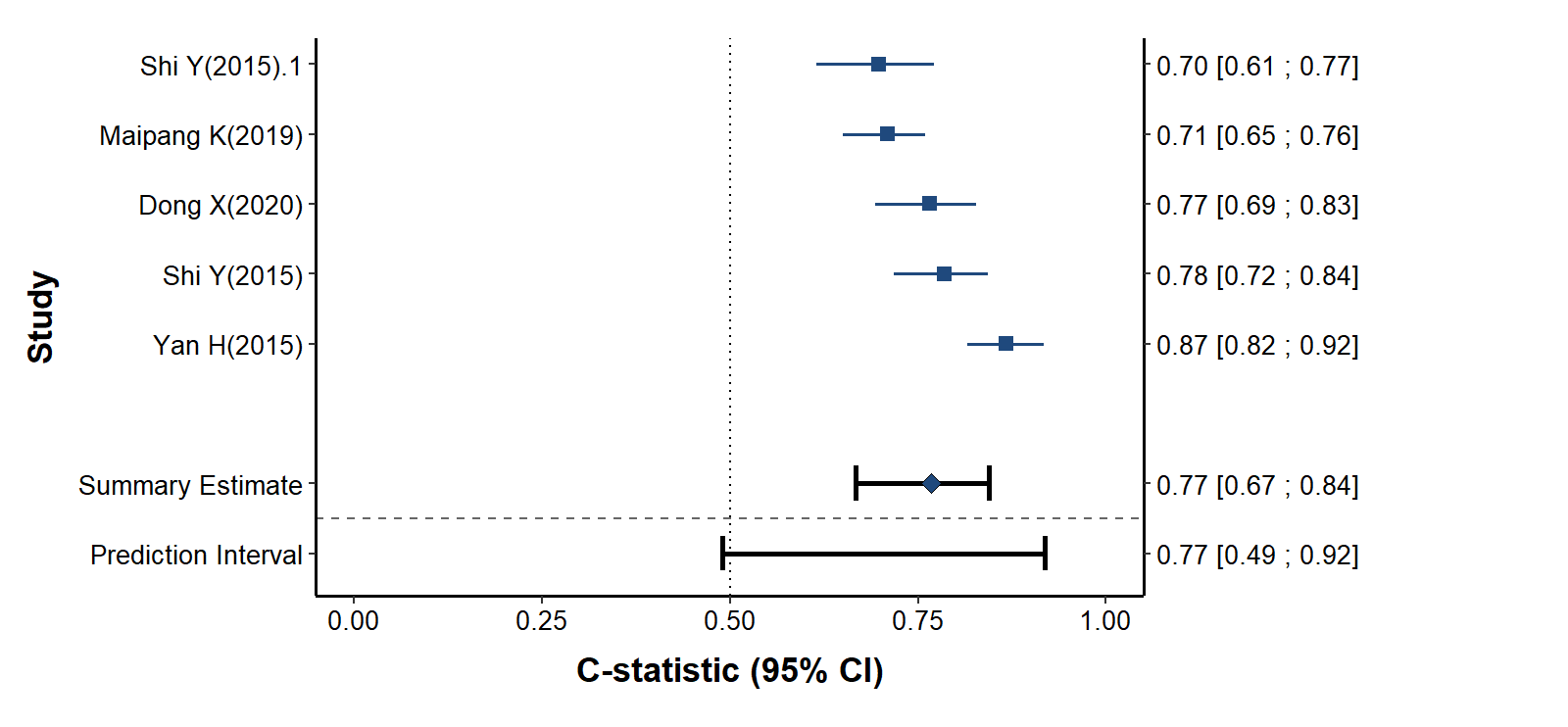


Note: Q =16.06 (df =4, p =0.0029); I²=77.61%; τ² =0.1175.

Figure 40. Forest plot of the i-MELD score for 3-month mortality.


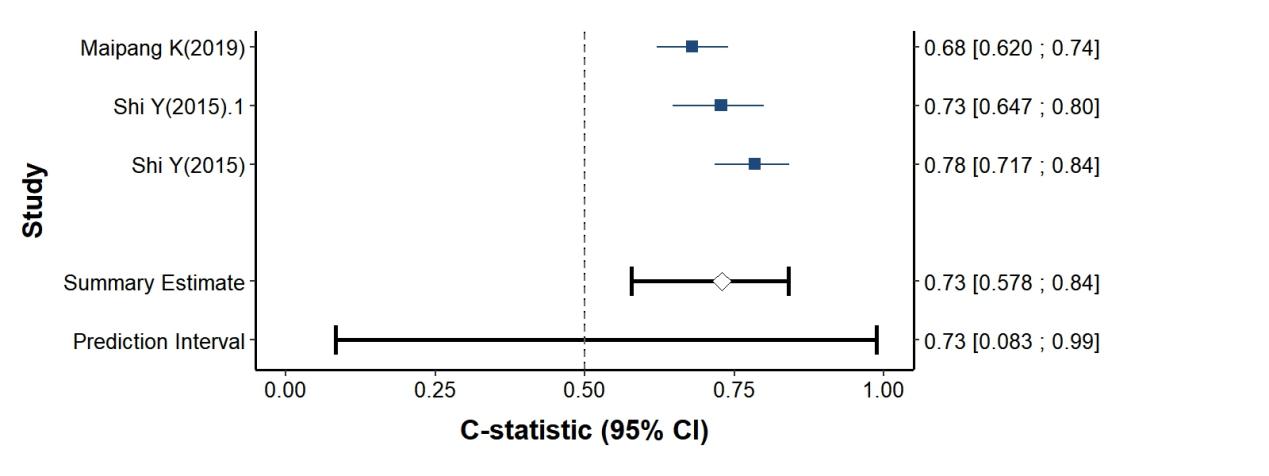


Note: Q =5.13 (df =2, p=0.0767); I²=60.30%; τ²=0.0465.

Figure 41. Forest plot of the i-MELD score for 1-year mortality.


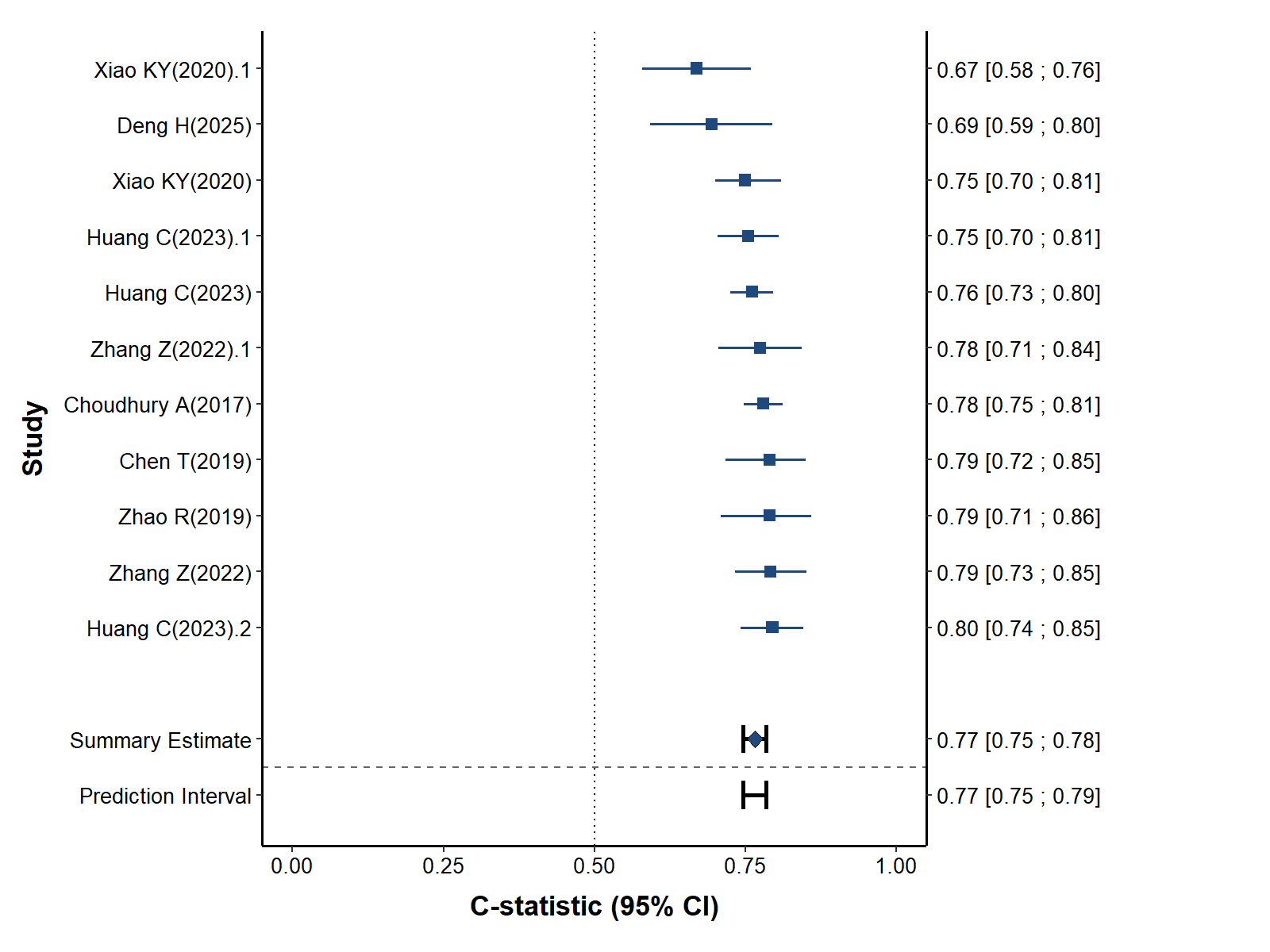


Note: Q =11.11 (df =10, p=0.3493); I²=0.01%; τ² =0.

Figure 42. Forest plot of the AARC score for 1-month mortality.


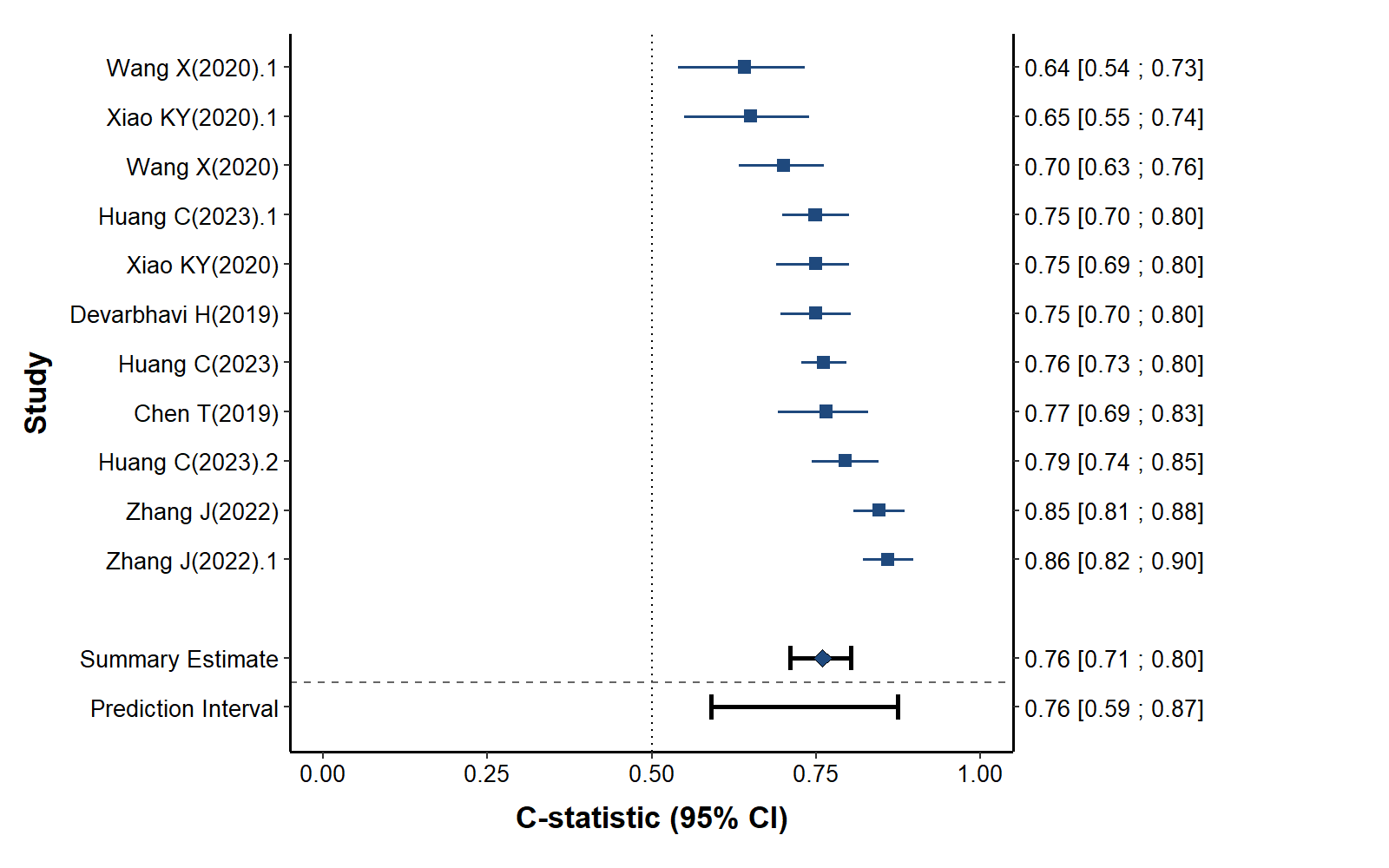


Note: Q =46.37 (df =10, p<0.0001); I²=81.96%; τ²=0.1082.

Figure 43. Forest plot of the AARC score for 3-month mortality.


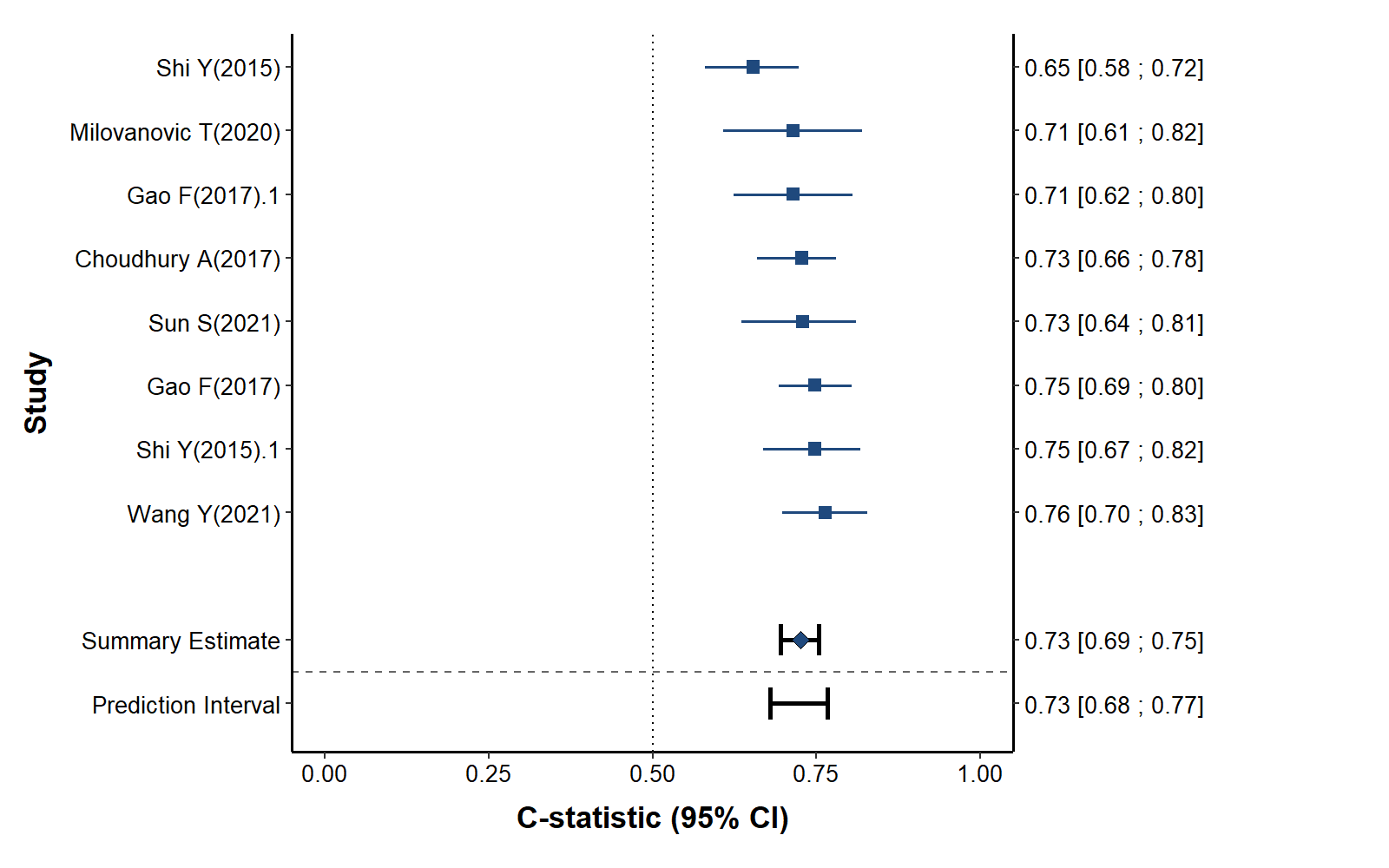


Note: Q =6.39 (df =7, p=0.4952); I²=10.29%; τ² =0.0041.

Figure 44. Forest plot of the SOFA score for 1-month mortality.


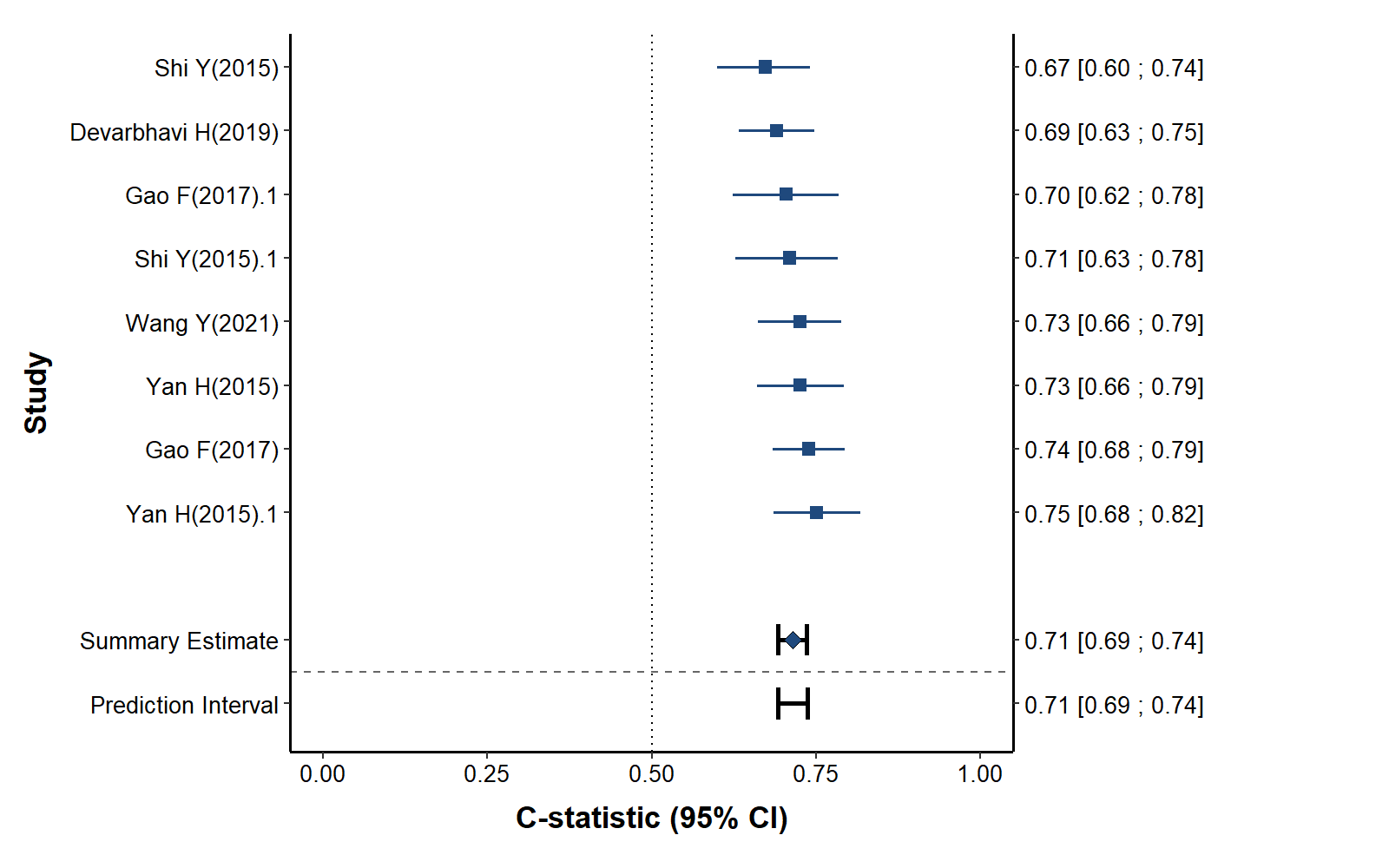


Note: Q =4.14 (df =7, p=0.7635); I²=0%; τ²=0.

Figure 45. Forest plot of the SOFA score for 3-month mortality.


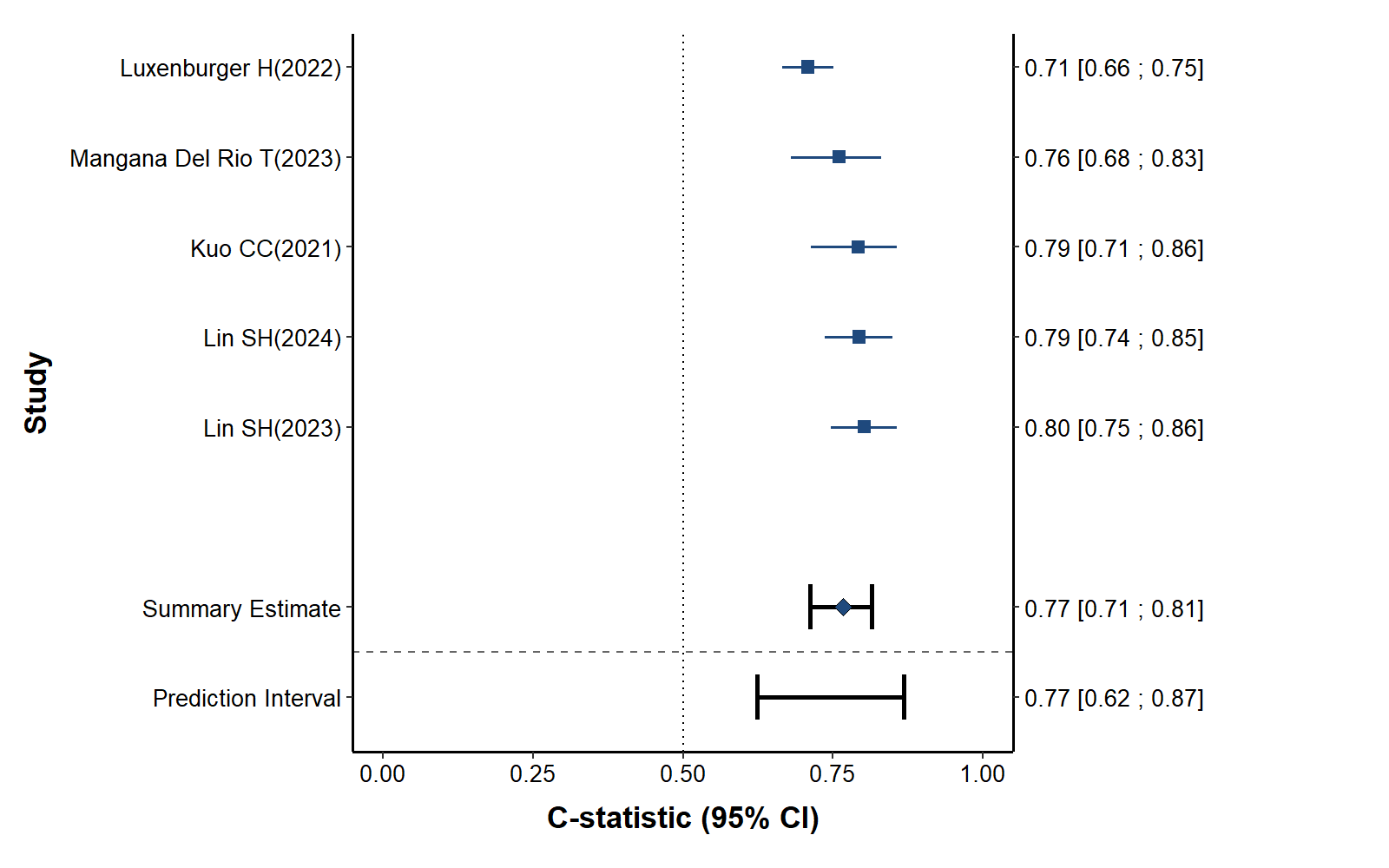


Note: Q =9.77 (df =4, p=0.0444); I²=55.45%; τ²=0.0362.

Figure 46. Forest plot of the CLIF-C-Lactate score for 1-month mortality.


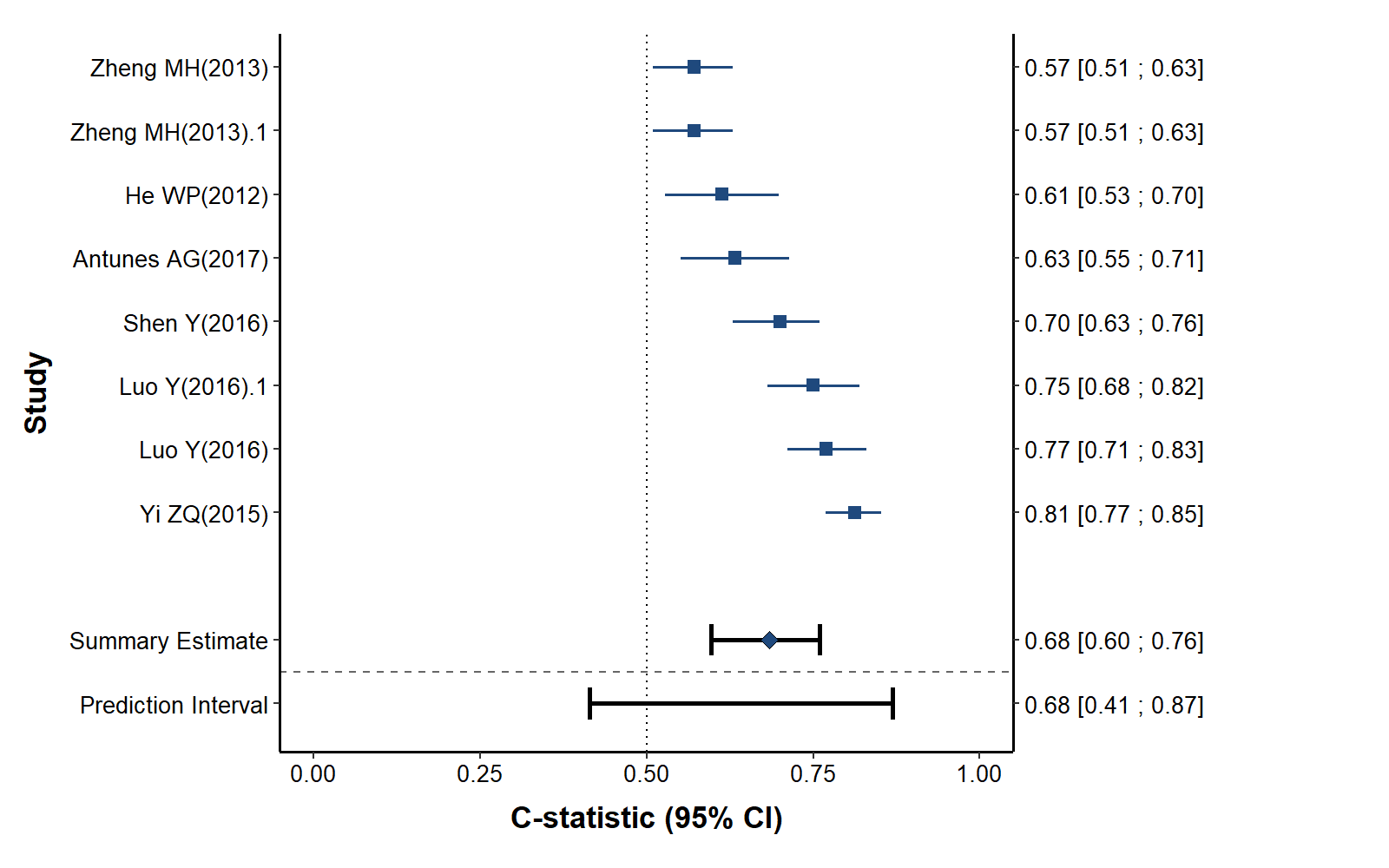


Note: Q=68.34 (df =7, p<0.0001); I²=88.44%; τ²=0.1833.

Figure 47. Forest plot of the MESO score for 3-month mortality.


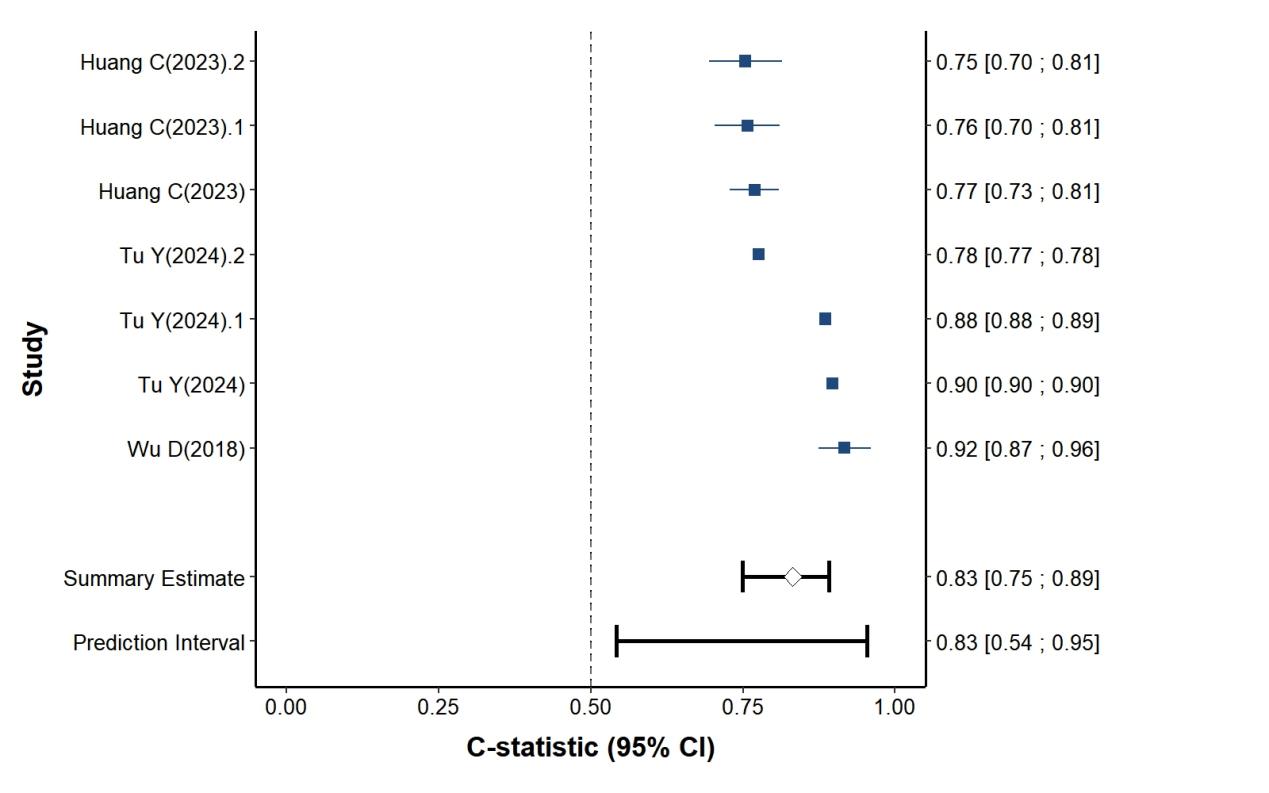


Note: Q =1064.49 (df =6, p<0.0001); I²=99.58%; τ²=0.2678.

Figure48. Forest plot of the HINT score for 1-month mortality.


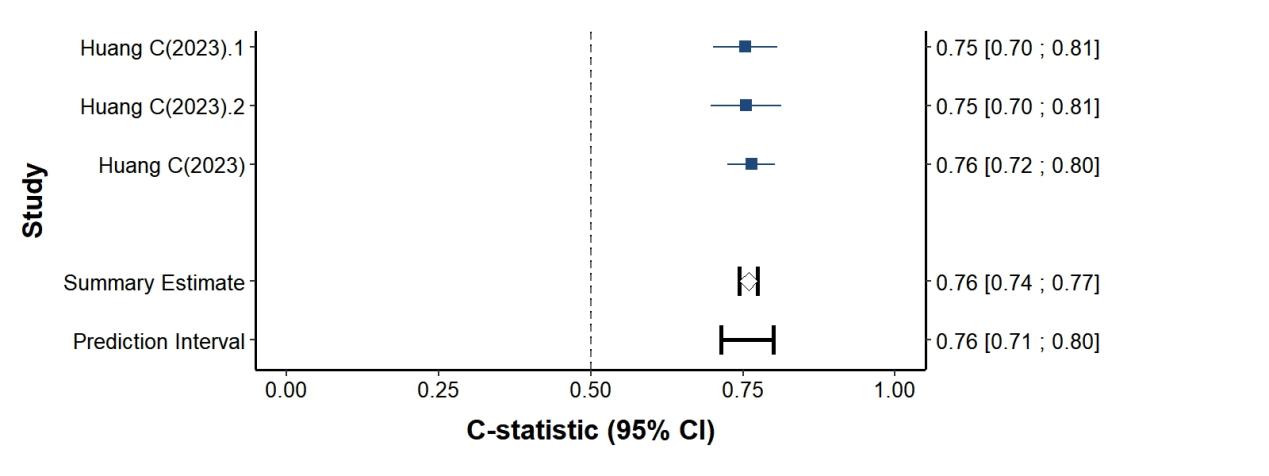


Note: Q =0.11c(df =2, p=0.9451); I²=0%;cτ² =0.

Figure 49. Forest plot of the HINT score for 3-month mortality.


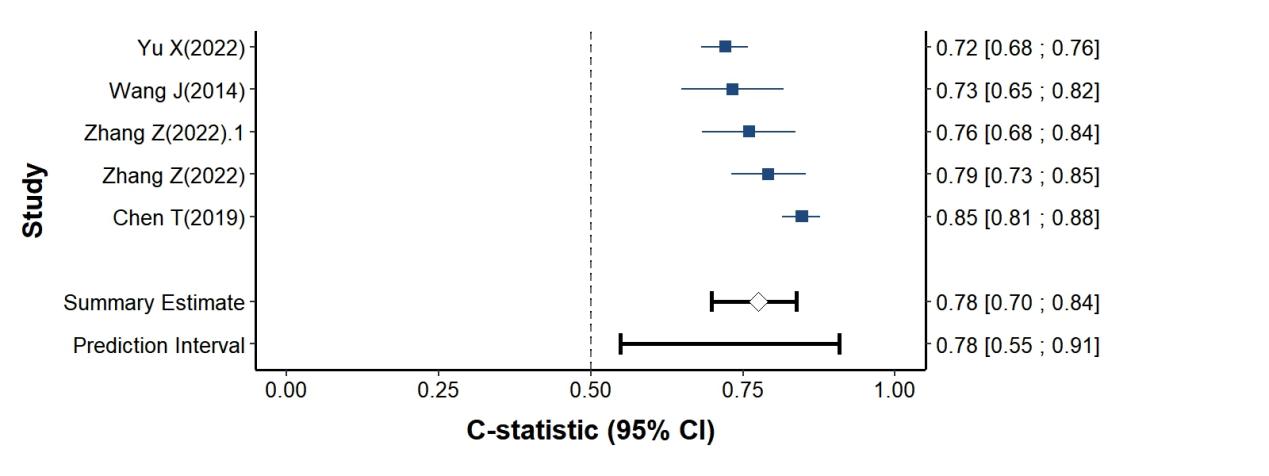


Note: Q = 24.50 (df =4, p<0.0001); I²=78.18%; τ²=0.0870.

Figure 50. Forest plot of the TPPM score for 1-month mortality.


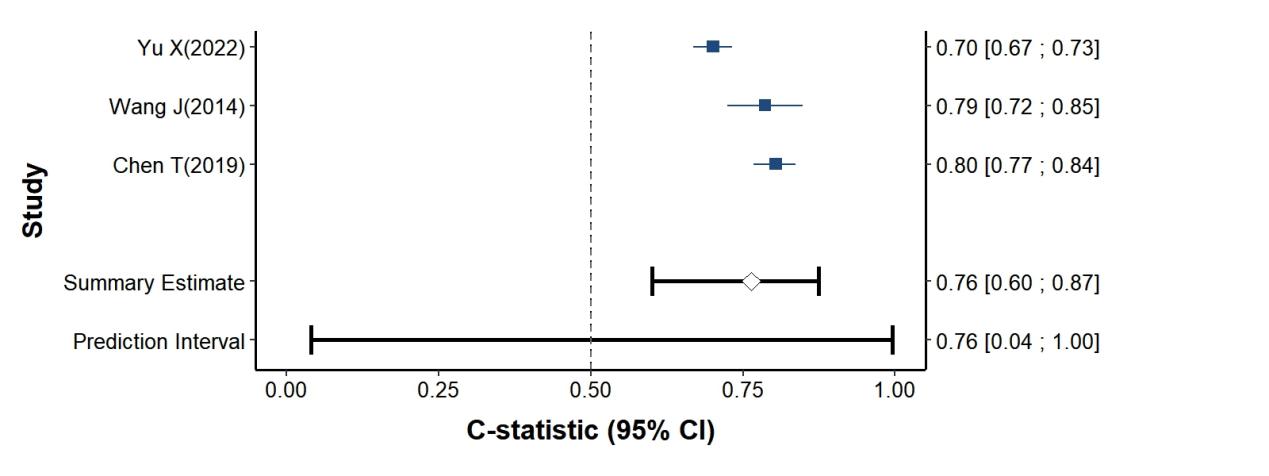


Note: Q = 18.75 (df =2, p<0.0001); I²=86.20%; τ²=0.0852.

Figure 51. Forest plot of the TPPM score for 3-month mortality.


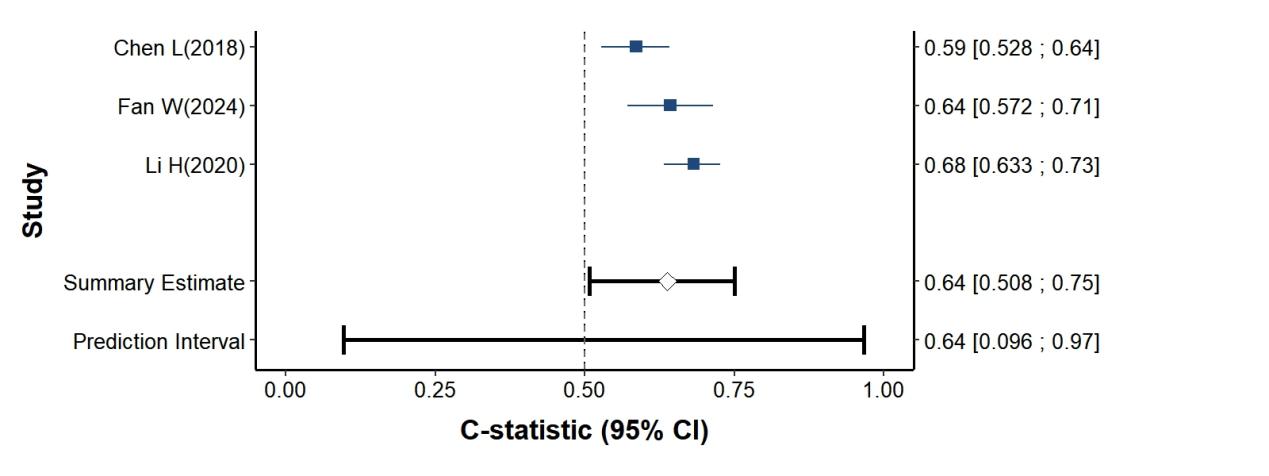


Note: Q =6.46 (df =2, p=0.0396); I²=66.9%; τ²=0.0334.

Figure 52. Forest plot of the ALBI score for 1-month mortality.


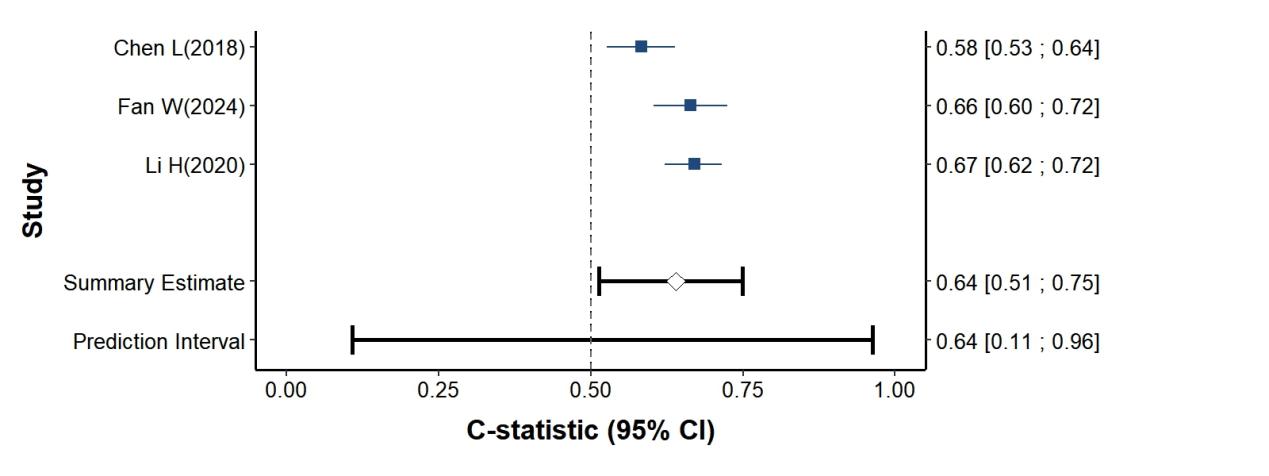


Note: Q =6.13 (df =2, p=0.0466); I² =66.77%; τ² = 0.0298.

Figure 53. Forest plot of the ALBI score for 3-month mortality.


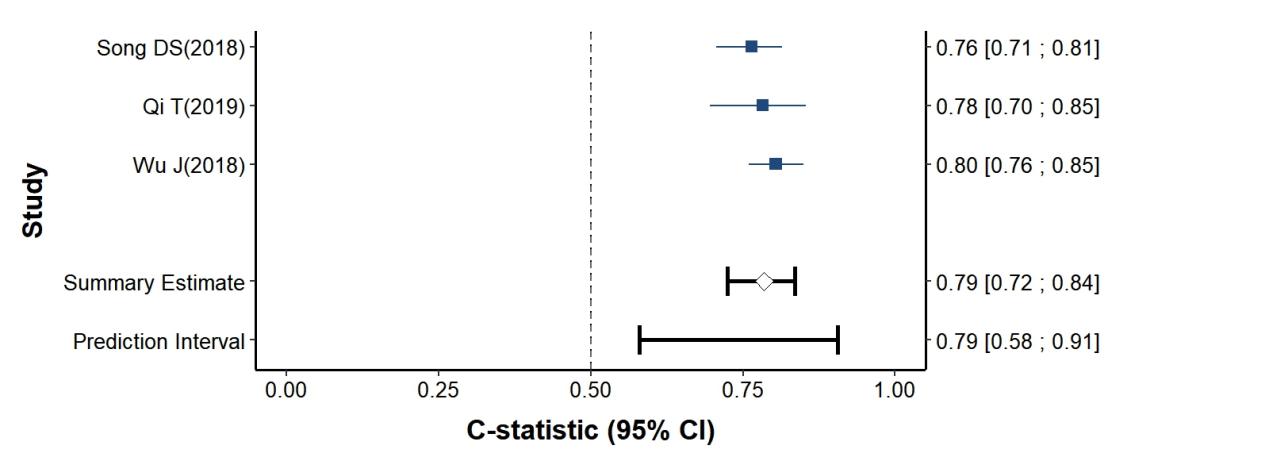


Note: Q =1.22 (df =2, p=0.5429); I² =0%; τ² =0.

Figure 54. Forest plot of the CLIF-C AD score for 1-month mortality.


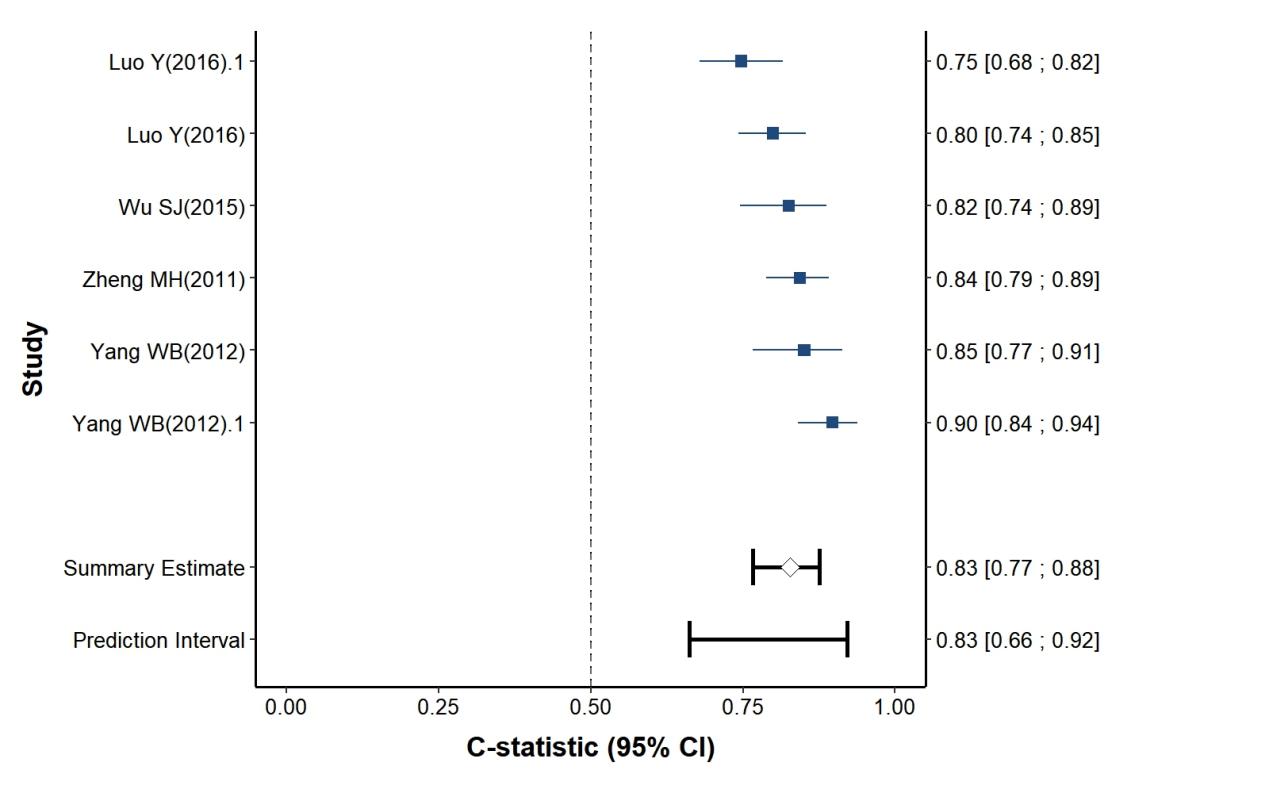


Note: Q =13.01 (df =5, p=0.0233); I²=62.06%; τ²=0.0812.

Figure 55. Forest plot of the LRM score for 3-month mortality.


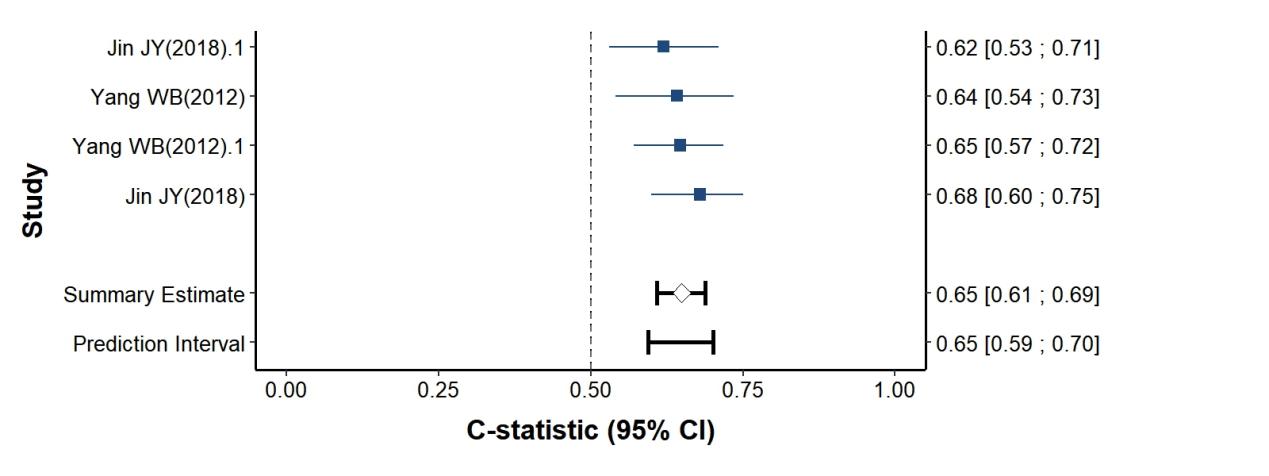


Note: Q =1.04 (df =3, p=0.7914); I² =0%; τ² =0.

Figure 56. Forest plot of the KCH score for 3-month mortality.


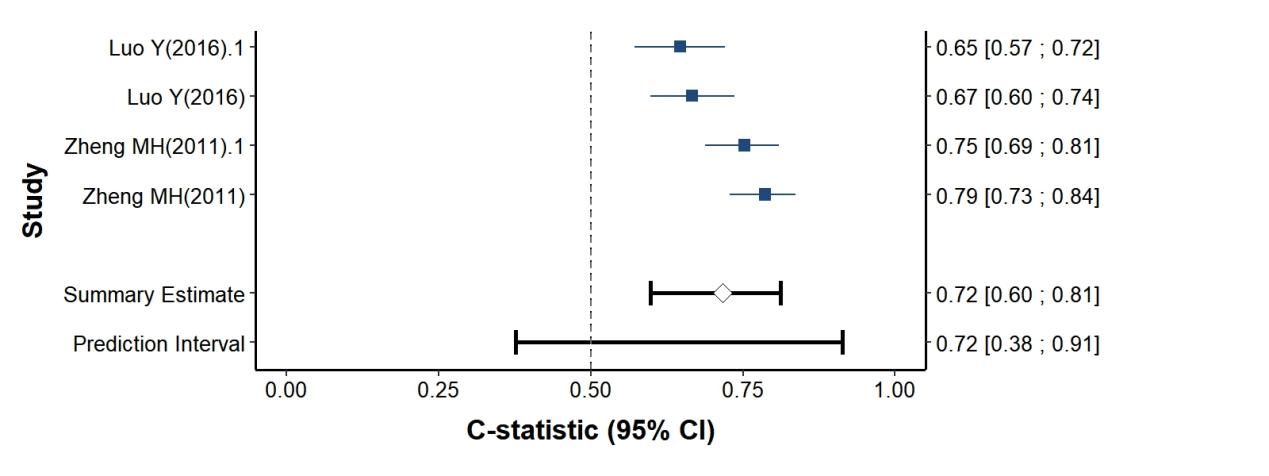


Note: Q =12.28 (df =3, p=0.0065); I²=75.56%; τ² =0.0836.

Figure 57. Forest plot of the SUN's model for 3-month mortality.


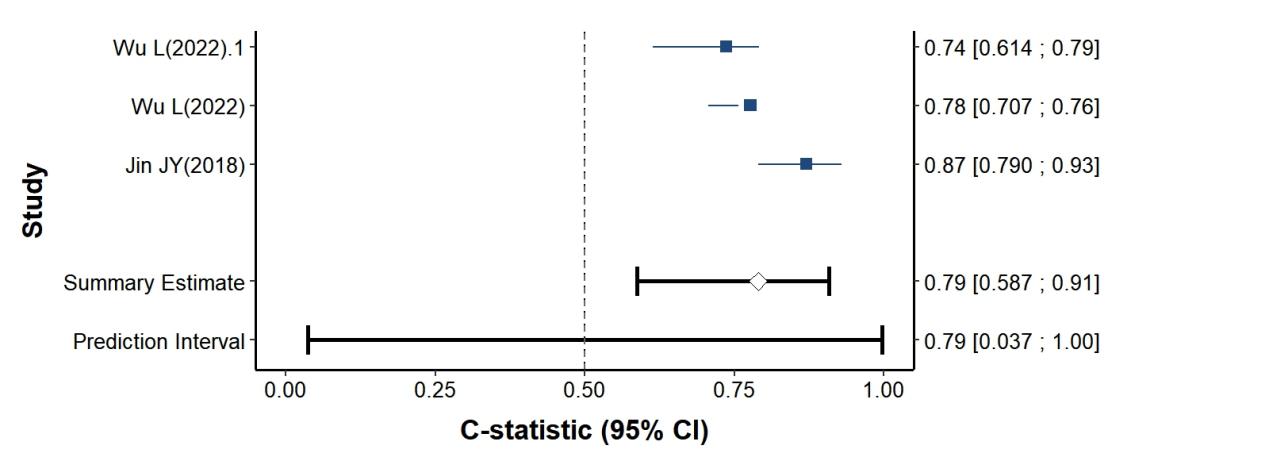


Note: Q =5.05 (df =2, p=0.0799); I²=68.33%; τ²=0.0793.

Figure 58 Forest plot of the MELD-SWE score for 3-month mortality
