# Supplementary material for: Prediction models for mortality in patients with acute on chronic liver failure: systematic review and critical appraisal
Source: Front Med (Lausanne). 2026 Jun 16;13:1829188. doi: 10.3389/fmed.2026.1829188 (PMC13314772; doi:10.3389/fmed.2026.1829188)
Supplement: Supplementary file 6 [file Table_6.DOCX]

PUBMED

#1 "Acute-On-Chronic Liver Failure"[Mesh] OR ACLF[Title/Abstract] OR "Failure, Acute-On-Chronic Liver"[Title/Abstract] OR "Liver Failure, Acute-On-Chronic"[Title/Abstract] OR "Acute-On-Chronic Liver Failure"[Title/Abstract]

#2  "Liver Failure"[Mesh] OR "liver failure"[Title/Abstract] OR "hepatic failure"[Title/Abstract]

#3 "acute-on-chronic"[Title/Abstract]

#4 #2 AND #3

#5 #1 OR #4

#6 "Mortality"[Mesh] OR mortality[Title/Abstract] OR death[Title/Abstract] OR survival[Title/Abstract]

#7 "risk assessment"[Mesh] OR "Risk Factors"[Mesh] OR "Area Under Curve"[Mesh] OR "ROC Curve"[Mesh] OR "Sensitivity and Specificity"[Mesh] OR predict*[Title/Abstract] OR progn*[Title/Abstract] OR "risk prediction"[Title/Abstract] OR "risk score"[Title/Abstract] OR "risk calculation"[Title/Abstract] OR "risk evaluation"[Title/Abstract] OR "risk index"[Title/Abstract] OR "risk assessment"[Title/Abstract] OR "risk factors"[Title/Abstract] OR model[Title/Abstract] OR "c-statistic"[Title/Abstract] OR discrimination[Title/Abstract] OR calibration[Title/Abstract] OR specificity[Title/Abstract]OR sensitivity[Title/Abstract] OR AUC[Title/Abstract] OR ROC[Title/Abstract] OR AUROC[Title/Abstract] OR "area under the curve"[Title/Abstract] OR "area under the receiver operator characteristic curve"[Title/Abstract]

#8 #5 AND #6 AND #7

Web of science

**#1 TS=(****"acute-on-chronic") AND TS=(****"liver failure" OR "hepatic failure")**

#2 TS=(ACLF OR **"**Failure, Acute-On-Chronic Liver**"** OR **"**Liver Failure, Acute-On-Chronic**"** OR **"**Acute-On-Chronic Liver Failure**"**)

#3 #1 OR #2

#4 **TS=(****mortality OR death OR survival)**

#5 **TS=****(**predict* OR progn* OR "risk prediction" OR "risk score" OR "risk calculation" OR "risk evaluation" OR "risk index" OR "risk assessment" OR "risk factors" OR model OR "c-statistic" OR discrimination OR calibration OR specificity OR sensitivity OR AUC OR ROC OR AUROC OR "area under the curve" OR "area under the receiver operator characteristic curve")

**#6 #3 AND #4 AND #5**

Embase

#1 'acute on chronic liver failure'/exp OR ACLF:ti,ab,kw OR 'Liver Failure, Acute-On-Chronic':ti,ab,kw OR 'Failure, Acute-On-Chronic Liver':ti,ab,kw OR 'Acute-On-Chronic Liver Failure':ti,ab,kw

#2 'liver failure'/exp OR 'liver failure':ti,ab,kw OR 'hepatic failure':ti,ab,kw

#3 'acute-on-chronic':ti,ab,kw

#4 #2 AND #3

#5 #1 OR #4

#6 'mortality'/exp OR mortality:ti,ab,kw OR death:ti,ab,kw OR survival:ti,ab,kw

#7 'risk assessment'/exp OR 'risk factor'/exp OR 'area under the curve'/exp OR 'receiver operating characteristic'/exp OR predict*:ab,ti OR progn*:ab,ti OR 'risk prediction':ab,ti OR 'risk score':ab,ti OR 'risk calculation':ab,ti OR 'risk evaluation':ab,ti OR 'risk index':ab,ti OR 'risk assessment':ab,ti OR 'risk factors':ab,ti OR model:ab,ti OR 'c-statistic':ab,ti OR discrimination:ab,ti OR calibration:ab,ti OR specificity:ab,ti OR sensitivity:ab,ti OR auc:ab,ti OR roc:ab,ti OR auroc:ab,ti OR 'area under the curve':ab,ti OR 'area under the receiver operator characteristic curve':ab,ti

#8 #5 AND #6 AND #7

Cochrane library

#1 MeSH descriptor: [Acute-On-Chronic Liver Failure] explode all trees OR (ACLF OR "Liver Failure, Acute-On-Chronic" OR "Failure, Acute-On-Chronic Liver" OR "Acute-On-Chronic Liver Failure"):ti,ab,kw

#2 MeSH descriptor: [Liver Failure] explode all trees OR ("liver failure" OR "hepatic failure"):ti,ab,kw

#3 ("acute-on-chronic"):ti,ab,kw

#4 #2 AND #3

#5 #1 OR #4

#6 MeSH descriptor: [Mortality] explode all trees OR (mortality OR death OR survival):ti,ab,kw

#7 MeSH descriptor: [Risk Assessment] explode all trees OR MeSH descriptor: [Risk Factors] explode all trees OR MeSH descriptor: [Area Under Curve] explode all trees OR MeSH descriptor: [ROC Curve] explode all trees OR MeSH descriptor: [Sensitivity and Specificity] explode all trees OR (predict* OR progn* OR "risk prediction" OR "risk score" OR "risk calculation" OR "risk evaluation" OR "risk assessment" OR "risk index" OR "risk factors" OR model OR "c-statistic" OR discrimination OR calibration OR AUC OR AUROC OR "area under the curve" OR "area under the receiver operator characteristic curve" OR ROC):ti,ab,kw

#8 #5 AND #6 AND #7
